# A cryptic syngameon within *Betula* shrubs revealed: Implications for conservation in changing subarctic environments

**DOI:** 10.1111/eva.13689

**Published:** 2024-04-17

**Authors:** Lyne Touchette, Julie Godbout, Manuel Lamothe, Ilga Porth, Nathalie Isabel

**Affiliations:** ^1^ Department of Wood and Forest Sciences Université Laval Quebec Quebec Canada; ^2^ Natural Resources Canada, Canadian Forest Service Laurentian Forestry Centre Quebec Quebec Canada; ^3^ Centre for Forest Research Université Laval Quebec Quebec Canada; ^4^ Ministère des Ressources naturelles et des Forêts, Direction de la recherche forestière Québec Québec Canada

**Keywords:** *Betula* spp., biogeography, genetic diversity, hybridization, ploidy, shrub, SNPs, syngameon

## Abstract

Arctic and subarctic ecosystems are rapidly transforming due to global warming, emphasizing the need to understand the genetic diversity and adaptive strategies of northern plant species for effective conservation. This study focuses on *Betula glandulosa*, a native North American tundra shrub known as dwarf birch, which demonstrates an apparent capacity to adapt to changing climate conditions. To address the taxonomic challenges associated with shrub birches and logistical complexities of sampling in the northernmost areas where species' ranges overlap, we adopted a multicriteria approach. Incorporating molecular data, ploidy level assessment and leaf morphology, we aimed to distinguish *B. glandulosa* individuals from other shrub birch species sampled. Our results revealed three distinct species and their hybrids within the 537 collected samples, suggesting the existence of a shrub birch syngameon, a reproductive network of interconnected species. Additionally, we identified two discrete genetic clusters within the core species, *B. glandulosa*, that likely correspond to two different glacial lineages. A comparison between the nuclear and chloroplast SNP data emphasizes a long history of gene exchange between different birch species and genetic clusters. Furthermore, our results highlight the significance of incorporating interfertile congeneric species in conservation strategies and underscores the need for a holistic approach to conservation in the context of climate change, considering the complex dynamics of species interactions. While further research will be needed to describe this shrub birches syngameon and its constituents, this study is a first step in recognizing its existence and disseminating awareness among ecologists and conservation practitioners. This biological phenomenon, which offers evolutionary flexibility and resilience beyond what its constituent species can achieve individually, may have significant ecological implications.

## 1 INTRODUCTION

The Arctic is warming at a faster pace than the rest of the planet (Post et al., 2019). To date, most climate models predict that circumpolar regions, that is, arctic and subarctic regions, will be the most impacted by climate change (CC; Pörtner et al., 2022; Serreze et al., 2000). Plant species of these regions are known to be responsive to global warming that directly influences their phenology (timing of budburst, flowering, etc.; Bjorkman et al., 2020; Myers‐Smith et al., 2015, 2020; Prevéy et al., 2017, 2019). CC could alter plant species diversity, composition, abundance and species range (Elmendorf et al., 2012; Le Moullec & Bender, 2022; Myers‐Smith et al., 2011). More specifically, temperature increases seem to contribute to widespread greening of tundra ecosystems (García Criado et al., 2020) and the expansion of the shrub layer, also known as shrubification (McManus et al., 2012; Myers‐Smith et al., 2011; Sturm et al., 2001; Tape et al., 2006). However, tundra greening is not homogeneous across the whole territory and may vary depending on ecological and physical factors (Berner et al., 2020; Hollister et al., 2005; Mekonnen et al., 2021; Myers‐Smith et al., 2020). Across the circum‐Arctic region, widespread *Betula* shrubs are increasing in abundance and are recognized as key indicators of CC impacts on northern ecosystems (Buchwal et al., 2022; Büntgen et al., 2015; Myers‐Smith et al., 2011).

The taxonomy of the genus *Betula* presents several challenges (Ashburner & McAllister, 2013). Since the 19th century, *Betula* taxonomy has mostly relied on morphological criteria, which has contributed to the debate surrounding the phylogenetic complexity within this genus (Ashburner & McAllister, 2013; De Jong, 1993; Regel, 1865; Skvortsov, 2002; Vasil'ev, 1969; Winkler, 1904). To date, there is no scientific consensus on the number of species within this genus, ranging from 30 to 120 species (Ashburner & McAllister, 2013; Furlow, 1990; Koropachinskii, 2013). In addition to the high intraspecific morphological variation, molecular data have also revealed other adaptive mechanisms that come into play in birches, such as polyploidy, as well as interspecific hybridization and introgression that could partly explain the complexity of the genus *Betula* (Anamthawat‐Jónsson, 2019; Ashburner & McAllister, 2013; Bona et al., 2018; Ding et al., 2021; Tsuda et al., 2017; Zohren et al., 2016). Moreover, Buck and Flores‐Rentería (2022) propose that birches can behave like a syngameon, a reproductive network of distinct interconnected species.

In North America, the dwarf birch (*Betula glandulosa* Michx.) is one of the species that has contributed the most to shrubification (Ropars et al., 2015; Ropars & Boudreau, 2012; Tape et al., 2006; Tremblay et al., 2012; Wolter et al., 2016). Unlike other northern plant species that are experiencing range contraction in response to CC (Alsos et al., 2012; Parmesan, 2006), dwarf birch instead appears to benefit from rising temperatures to expand its range. *Betula glandulosa* is a North American low Arctic tundra and alpine shrub species in the Betulaceae family (Ashburner & McAllister, 2013) and can persist in a wide variety of habitats, both dry and wet (Ashburner & McAllister, 2013; Furlow, 1997; Payette, 2015). It is also one of the most widespread shrub birches in North America (Ashburner & McAllister, 2013; Furlow, 1997). According to the latest taxonomic revision, this species is part of the *Apterocaryon* section, which includes other dwarf birches mainly located in the boreal, subarctic and Arctic regions of the northern hemisphere (Ashburner & McAllister, 2013). To date, there is still ambiguity regarding the taxonomy of *B. glandulosa* and its relationship with other birch species. In North America, Furlow (1997) and Ashburner and McAllister (2013) have identified over 10 different *Betula* species of trees and shrubs. Several of these species coexist in sympatry within specific regions of *B. glandulosa*'s distribution range and are known to hybridize with it. Notable examples include *B. pumila* L. throughout North America, *B. occidentalis* Hook. and *B. nana* L. in the western part of its range, and *B. cordifolia* Regel in the eastern part of its range (Ashburner & McAllister, 2013; Dugle, 1966; Furlow, 1997). Trait plasticity in *B. glandulosa* and its great resemblance to other shrub birches make it difficult to identify (Ashburner & McAllister, 2013; Furlow, 1997; Payette, 2015). Based on morphological characteristics, some authors consider *B. glandulosa* and *B. nana* ssp. *exilis* (Sukaczev) Hultén as two distinct taxa (Cody, 2000; de Groot et al., 1997; Elven et al., 2011; Furlow, 1997; Hultén, 1968), while others suggest that they represent the same taxon (Ashburner & McAllister, 2013; Hultén & Fries, 1986; Moss & Packer, 1983; Porsild & Cody, 1980; Saarela et al., 2017). To date, the phylogenetic relationship between *B. glandulosa* and other birch species remains relatively unknown (Li et al., 2005; Tarieiev et al., 2021; Wang et al., 2016, 2021).

*Betula glandulosa* has frequently been the subject of environmental studies addressing shrubification in the Arctic (Ropars et al., 2015; Ropars & Boudreau, 2012; Tremblay et al., 2012). Additionally, *B. glandulosa* has been investigated for its leaf chemical composition and defences against herbivory (Bryant et al., 2014; Lindén et al., 2022; Séguin et al., 2021). Despite these efforts, there is a notable gap in research concerning the genetic aspects of this species. Given its ecological significance in northern ecosystems as a key indicator of CC, and recognizing the importance of monitoring the species, a more comprehensive exploration of its genetic diversity could not only yield insights into its phylogenetic status but also shed light on its adaptive strategies. Acknowledging the importance of extensive documentation of CC impacts on northern ecosystems, genomics has been identified as a promising tool for studying species biogeography, population structure, resilience and adaptation to CC in the Arctic (Colella et al., 2020; Wullschleger et al., 2015).

In the current climate context, this study is part of a larger goal to develop genomics‐based knowledge about the species that inhabit northern ecosystems (Colella et al., 2020). Hence, considering the importance of *B. glandulosa* in understanding the impact of CC in Arctic and subarctic regions, our initial focus was to investigate the biogeography of this species within North America and characterize its genetic diversity. However, owing to its morphological resemblance to other shrub birch species, as well as the extensive sympatry observed in their natural ranges, we were aware that the taxonomic validation of dwarf birch could pose challenges. Therefore, the primary objective of our study was to identify congeneric shrub birches (other species) among the sampled individuals through a multicriteria approach, based on morphology and molecular data (genetic diversity and ploidy level assessment), to distinguish and delineate species (Bacon et al., 2012; Balao et al., 2020; Lissambou et al., 2019; Newton et al., 2020; Su et al., 2015; Wang et al., 2022; Zhang et al., 2023). We then focus on the biogeography of *B. glandulosa* and describe its genetic structure.

## 2 MATERIALS AND METHODS

### 2.1 Samples and DNA extraction

We gathered samples encompassing 80 populations from 537 putative *B. glandulosa* individuals, 527 of which were from Canada and Alaska, and the remainder from Greenland and Scandinavia. Samples from Greenland and Scandinavia were designated as European samples throughout this study. Most of the material collection was conducted in natural stands of limited access that necessitated sometimes the transportation by helicopter. The samples were provided by university researchers or collaborators from Canadian governmental organizations (e.g., national parks or natural resources departments; see Acknowledgements section). A total of 29 teams participated in the sampling in 2011, 2017 and 2018. Leaves and twigs from 1 to 17 individuals were sampled in each population; a minimum distance of 7–10 m between individuals was ensured in order to limit the risk of sampling clones. The sampling covered most of *B. glandulosa'*s North American range, except for Ontario and the northwestern United States (corresponding to the southernmost dwarf birch populations), for which no samples were available. We extracted DNA from dried leaves or buds using the Nucleospin 96 Plant II Kit (Macherey‐Nagel, Bethlem, PA, USA) following the manufacturer's instructions with one modification: 90 min instead of 30 min for the lysis step using buffer PL1 at 65°C. DNA from 537 samples was purified using SPRI magnetic beads (1× ratio; Beckman Coulter, Indianapolis, IN, USA) and quantified using a NanoDrop 8000 spectrophotometer (Thermo Fisher Scientific, Wilmington, DE, USA) prior to amplification and sequencing.

### 2.2 DNA sequencing and SNP marker development

SNP marker development was conducted using the Fluidigm ‘Targeted DNA sequencing’ method (San Francisco, CA, USA) following the manufacturer's instructions. A total of 479 primer pairs were designed to amplify 300–500‐bp genomic regions. The annotated *B. pendula* Roth genome assembly v.1.2 (Salojärvi et al., 2017), representing 89% of the 440‐Mb genome assembled into 14 chromosomal linkage groups, was used to select the target genomic regions for which primers were designed. Sequences from six *Betula* species (*B. nana*, *B. platyphylla* Sukaczev, *B. populifolia* Marsh., *B. occidentalis*, *B. lenta* L. and *B. pubescens* Ehrh.; Salojärvi et al., 2017), as well as sequences from the *B. nana* assembly (Wang et al., 2013), were aligned to the *B. pendula* assembly using the CLC Genomic Workbench v7.5.1 (Qiagen, Germantown, MD, USA). The use of sequence data from other species was employed to differentiate regions rich in polymorphisms from less variable regions and to optimize the selection of regions for primer design for the ‘Targeted DNA sequencing’ method. This also allowed for a balanced selection of conserved and polymorphic regions. The final selection included 302 gene regions with described functions, 150 with unknown function and 26 chloroplast regions (Table S1). Library construction, sequencing on a MiSeq system with the Reagent Kit V3 (Illumina, San Diego, CA, USA) and sequence demultiplexing were performed at the Plateforme Génome Transcriptome (INRAE, Bordeaux, France).

An initial cleaning and trimming step was performed using an in‐house script on the raw sequencing data by eliminating sequences having an average Phred score lower than 25 (averaged over a sliding window of 5 bp) and by eliminating reads having a size lower than 50 bp. Next, in order to distinguish nuclear from organellar sequences, we aligned the sequences with a single fasta file containing mitochondrial (NCBI LT855379.1) and nuclear sequences from the genome assembly of *B. pendula* (v.1.2; Salojärvi et al., 2017), and chloroplast sequences from the *B. nana* chloroplast assembly (NCBI KX703002.1; Hu et al., 2017) using bwa (Li & Durbin, 2009). For nuclear variants, genotype likelihood was determined using bcftools mpileup (subpackage of SAMtools; Li et al., 2009), keeping only SNPs with a minimal probability of 0.01 (‐*p* 0.01) and total depth of 5000 sequences (INFO/DP > 5000). The genotype calls were made with vcftools v0.1.16 (Danecek et al., 2011), replacing genotypes with <5 sequences depth by missing values (‐minDP 5). Organellar variants were called with VarScan v2.3.9 (Koboldt et al., 2012) using the ‘‐min‐var‐freq 0.35’ and ‘‐min‐freq‐for‐hom 0.5’ as parameters. Variants were only detected within nuclear and chloroplast genomes, and only bi‐allelic SNPs were kept.

### 2.3 Ploidy level determination

At the onset of the study, all collected individuals were initially presumed to be *B. glandulosa* and thus diploids. However, *B. glandulosa* shares most of its range with *B. pumila*, a tetraploid species that has similar morphological characteristics (Ashburner & McAllister, 2013). Ploidy level can therefore be used as an additional tool in a multicriteria approach to distinguish species. We assessed the ploidy level of each individual indirectly by looking at the density profiles of the allelic ratios at heterozygous sites according to the principle used by Zohren et al. (2016). To refine these profiles, and because we expected a high level of complexity within our dataset, the curves were determined from a subset of SNPs. This filtration procedure allowed to avoid SNPs with unspecific alignments of sequences that would make the heterozygote SNPs deviate from their true ratio at 0.5 (for diploid individuals). The use of this subset aimed to eliminate non‐informative secondary peaks, ensuring minimal ambiguity in assigning profiles of individuals to a specific ploidy.

We first divided the entire SNPs dataset into a subset by filtering them using only individuals with a clear diploid profile (*n* = 246). We then filtered the SNPs for those with an average coverage of 24×, presenting a distribution mode between 0.45 and 0.55 (after exclusion of the ratios below 0.05 and over 0.95), and with two‐thirds of the AUC (area under the curve) between 0.4 and 0.6. Ratio values calculated with <15 sequences were considered as missing. The resulting reduced dataset of 267 SNPs was used to generate density profiles of allelic ratios and to visually determine the ploidy level. A single peak near a ratio of 0.5 would represent the diploid individuals, two peaks near 0.33 and 0.66 would represent the triploids, while three peaks near 0.25, 0.5 and 0.75 would represent the tetraploids, and four peaks near 0.2, 0.4, 0.6 and 0.8 would represent some pentaploids. The remaining profiles were classified as unknown, as they presented multiple peaks that could not be assigned to any of the previously mentioned patterns.

### 2.4 Data preparation and filtering

The 479 primer pairs generated alignments to 457 distinct genomic regions. Two nucleic marker datasets were created for the subsequent genetic data analyses. The first SNP dataset, hereafter named the ‘initial diploid’ dataset (Table S2), was coded as diploid, assuming that all individuals were putatively *B. glandulosa*. This dataset was used for multivariate analyses, genetic diversity analyses and STRUCTURE (Table 1). The second dataset (termed ‘diploid‐tetraploid’) was coded as diploid or tetraploid after the ploidy level was deduced for each individual (see Section 2.3 and Table 1). The missing data limit per individual for each dataset was determined to maximize the number of samples and obtain balanced datasets. In addition, a third dataset of chloroplast markers was used to derive the haplotype connectivity among samples (see Section 2.7).

**TABLE 1:** Summary of datasets used in the different analyses.

Datasets	Number of loci	Number of genotypes	Analyses
Initial diploid nuclear SNPs	5033	469	DAPC, STRUCTURE
			Genetic diversity (*H*_O_, *H*_E_, *F*_IS_, *F*_ST_)
Diploid‐tetraploid nuclear SNPs	3481	453	STRUCTURE
Haploid chloroplast SNPs	41	452	Haplotype diversity

#### 2.4.1 Initial diploid dataset

To generate this dataset, we used the diploid genotype data initially containing all 537 individuals. We removed individuals with more than 40% missing data and retained polymorphic loci (MAF ≥0.01) with <30% missing data. To detect the presence of clones, the *mlg* function from the poppr R package was used to calculate the genetic distance between individuals (Kamvar et al., 2014). Individuals were assigned to clones based on a threshold of 350 for the genetic distance between individuals. We retained 469 individuals with unique genotypes (genets) and 5033 polymorphic SNPs after these filters.

#### 2.4.2 Diploid‐tetraploid dataset

The second dataset was created using a different input file containing all 537 individuals. Following the method presented in Zohren et al. (2016), all estimated tetraploid and non‐diploid ploidy levels were coded as tetraploid individuals (A/A/A/A). Diploid individuals were coded as diploid, while individuals with an ambiguous (see below) ploidy level were instead coded as tetraploid. From this dataset, individuals with more than 50% of missing data were removed and we retained the polymorphic loci (MAF ≥0.01) with <30% of missing data. The same approach as the one used for the initial diploid dataset was employed to detect the presence of clones, except that individuals were assigned to clones based on a threshold of 300 for the genetic distance between individuals. We retained 453 individuals and 3481 polymorphic SNPs after data filtering.

### 2.5 Population genetic structure

Considering the lack of knowledge regarding the sampled species (clonality, interspecific hybridization, etc.), and given that some assumptions might not be fulfilled (linkage disequilibrium and panmictic populations), we inferred population structure using two different methods: discriminant analysis of principal components (DAPC), a model‐free method (Jombart et al., 2010) and STRUCTURE (Pritchard et al., 2000).

From the initial diploid dataset, we performed a DAPC (Jombart et al., 2010) using the adegenet R package (Jombart, 2008). The initial step of the DAPC consists of a principal component analysis (PCA) to transform the genetic data into uncorrelated PC variables that are subsequently used in a discriminant analysis. This maximizes variation between genetic groups and minimizes variation within genetic groups. The DAPC does not account for Hardy–Weinberg equilibrium or linkage equilibrium constraints. We first conducted a DAPC without giving any prior information for clustering. We used the function *find. clusters* to assess the number of groups. This function allows the identification of clusters by using *k*‐means clustering and the optimal number of genetic groups was estimated using the Bayesian information criterion (BIC; lowest value; Jombart et al., 2010). The maximum number of clusters was fixed at 70. Fifty PCs were retained based on the alpha‐score obtained with the function *optim.a.score*.

Using the same initial diploid dataset, we also ran the Bayesian program STRUCTURE v. 2.3.4 (Pritchard et al., 2000). The admixture model was employed without using any prior population information and with the default parameters, except for the Alpha parameter. To take into account unbalanced sampling to which STRUCTURE is sensitive, we used the alternative ancestry prior (i.e., ‘Separate Alpha for each population’) with the initial Alpha value being 1/*K* (depending on the *K* value) as proposed by Wang (2017). An unbalanced sampling can result in a misestimate of the *K* number by STRUCTURE (Wang, 2017). For each run, a burn‐in period of 10,000 Markov Chain Monte Carlo (MCMC) iterations was followed by 100,000 MCMC iterations. Ten repeats were performed for each *K* value ranging from *K* = 1–10. The optimal number of clusters was determined by using the ∆*K* method outlined by Evanno et al. (2005) and the STRUCTURE Harvester program (Earl & vonHoldt, 2012).

We also performed a STRUCTURE analysis with the mixed‐ploidy dataset (individuals coded as either diploid or tetraploid) using the same procedure as described by Zohren et al. (2016), where the diploid samples were coded as tetraploid samples (four lines per individual) with the two last lines consisting of missing data (‐9) in the input STRUCTURE file. The parameters used were the same as those presented above for the initial diploid dataset.

### 2.6 Nuclear genomic diversity

The function *basic.stats* of the hierfstat R package (Goudet, 2005) was used to calculate observed heterozygosity (*H*_o_), expected heterozygosity (*H*_e_) and the inbreeding coefficient (*F*_IS_) for each population. We used the STRUCTURE results obtained with the diploid‐tetraploid dataset for *K* = 4 to assign each individual (*Q*‐value ≥0.75) to a specific genetic group, hereafter named ‘genetic lineage’. We separated the Alaskan from the European lineage even if these two lineages were combined into one genetic cluster by STRUCTURE to respect their geographical separation. Pairwise *F*_ST_ was estimated between each DAPC cluster and between each STRUCTURE genetic lineage (as defined by the STRUCTURE genetic clusters) using the Weir and Cockerham (1984) method implemented in the *genet.dist* function. Using the initial diploid dataset, the observed heterozygosity per individual was extracted from the vcf file using the *het* function from vcftools (Danecek et al., 2011).

### 2.7 Chloroplast haplotype diversity

We identified 85 bi‐allelic SNPs throughout the chloroplast genome. After filtering for missing data and information redundancy, 41 uniquely informative SNPs were retained for the analysis and to determine the haplotypes. For the 469 individuals in the initial diploid dataset, 20 different cpDNA sequences were obtained by concatenating the retained SNPs. There were 17 individuals without haplotype information due to missing data, leaving a total of 452 individuals with haplotype assignment.

### 2.8 Morphology

When leaf samples were still available after the genomics analyses, we used leaf morphology a posteriori, notably leaf shape (teeth, size and blade shape), as an additional criterion to discriminate species based on *Betula* leaf characteristics described in Furlow (1997), Ashburner and McAllister (2013) and Payette (2015). Table 2 highlights the morphological criteria used to distinguish species. As our genomic data could only provide us with indirect evidence (ploidy and membership of a genetic group), the goal was to confirm the presumed taxonomic identity of our samples previously identified through genomics analyses. A total of 309 individuals were observed.

**TABLE 2:** Morphological characteristics used to discriminate *Betula* shrub species within the sampling based on Furlow (1997) and Ashburner and McAllister (2013).

Species	Leaf blade	Leaf size (cm)	Leaf margins	Teeth shape	Number of veins
*Betula glandulosa*	Obovate—orbicular	0.5–3 × 1–2.5	Dentate—crenate	Obtuse to rounded	2–6
*Betula pumila*	Elliptic, obovate to almost orbicular	2.5–5 × 1–5	Crenate—dentate	Apex acute, obtuse to rounded	2–6
*Betula occidentalis*	Ovate—rhombic‐ovate	2–5.8 × 1–4.5	Sharply serrate or irregularly doubly serrate	Long and sharp	2–6
*Betula nana* ssp. *exilis*	Orbiculate—reniform	0.5–1.2 × 0.5–1.6	Deeply crenate	Apex rounded	2–6

## 3 RESULTS

### 3.1 Genetic structure and ploidy

**FIGURE 1:**
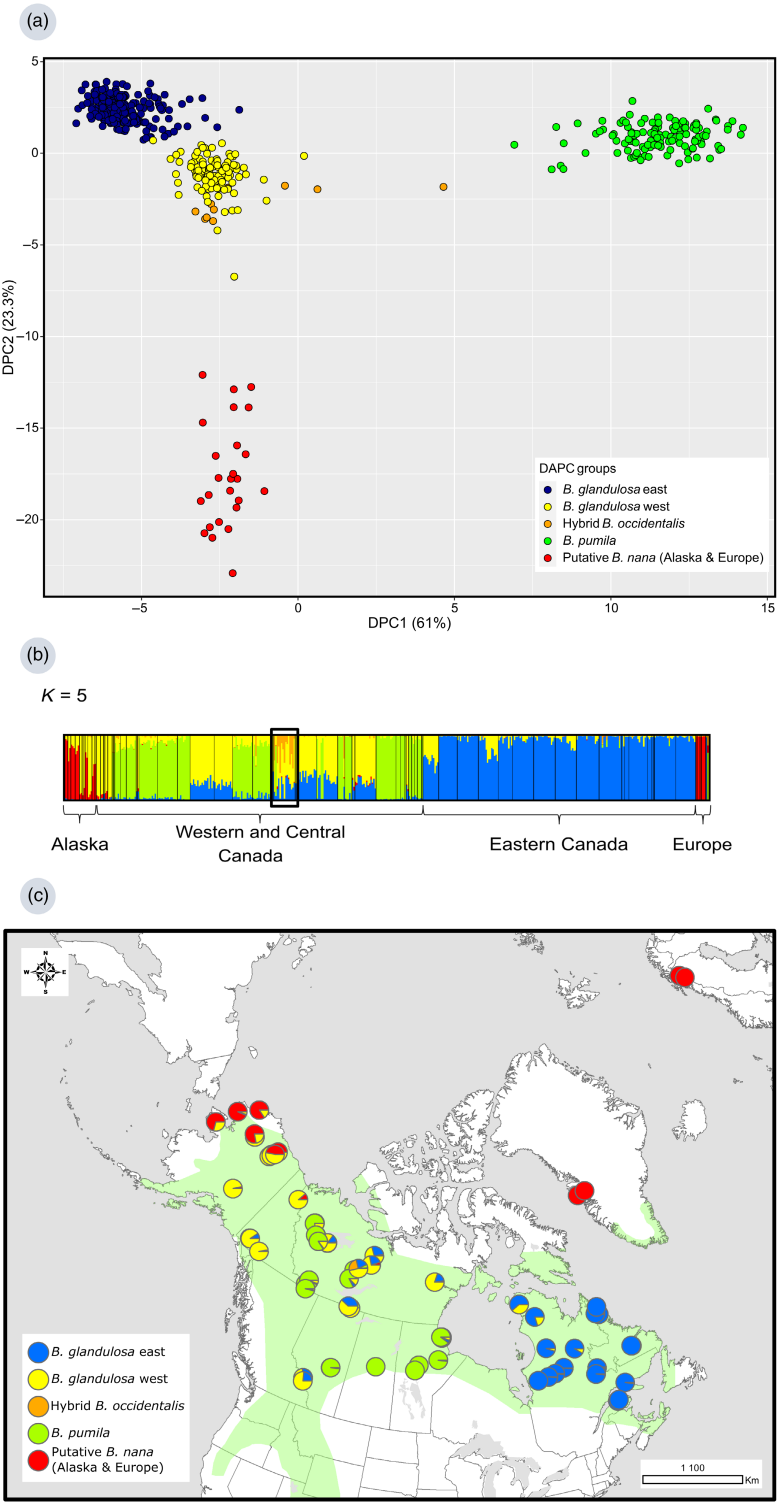
(a) Discriminant analysis of principal components (DAPC) scatterplot for the initial diploid dataset. (b) STRUCTURE barplots for the diploid‐tetraploid dataset for *K* = 5. Individuals are represented following a longitudinal gradient: on the far left, individuals from Alaska and on the far right, individuals from eastern Canada and Europe. The black‐framed rectangle indicates a population of hybrid individuals detected only at *K* = 5. (c) STRUCTURE results for the *K* = 5 partition obtained with the diploid‐tetraploid dataset. The green area represents the natural distribution range of *Betula glandulosa* according to Furlow (1997).

A DAPC was performed on the initial diploid dataset which identified five distinct genetic clusters based on BIC values (Figure 1a). In fact, the lowest BIC values were observed for *K* = 4–6 (Figure S1A), but we retained *K* = 5 because it better reflected the geographical and morphological differences among individuals (Figure S1B). The first axis explained 61% of the genetic variance while the second axis 23.3% (Figure 1a). The first axis revealed the separation between ‘tetraploid’ and ‘non‐diploid’ individuals (collectively shown as the green cluster, and hereafter referred to as *B. pumila*; see Section 4) from all other *Betula* individuals (Figure S2), while the second axis distinguished the geographically separated Alaskan and European shrub birches (red cluster) from the Canadian individuals (yellow, blue, orange and green clusters; Figure S3). The blue and yellow groups, which represented respectively the eastern and western clusters of *B. glandulosa*, were relatively close genetically (Figure 1a). The number of individuals in each genetic cluster was highly variable (Table 3). The *B. glandulosa* east cluster, containing 198 individuals, was the most abundant group. The *B. glandulosa* west cluster contained 109 individuals. More than 25% of the sampled individuals fell within the green cluster of *B. pumila* (127 individuals). The hybrid *B. occidentalis* group (10 individuals) was closest to the *B. glandulosa* west group, and in fact, both groups overlapped.

**TABLE 3:** Number of individuals per genetic cluster identified with the discriminant analysis of principal components (DAPC) with the initial diploid dataset within the sample.

Genetic clusters (DAPC groups)	Number of individuals
*Betula glandulosa* east	198
*Betula glandulosa* west	109
Hybrid *Betula occidentalis*	10
*Betula pumila*	127
Putative *Betula nana* (Alaska—Europe)	25
Total	469

After these preliminary analyses, the distribution of allelic ratios at heterozygous sites was examined and at least two different ploidy levels within the sampling were confirmed (Table 4): the presence of diploid and tetraploid individuals (Figure S4). Geographically, tetraploids were mainly restricted within Western and Central Canada (Figure S5). Overall, we identified 291 diploids (62.0% of all individuals; Figure S4A,B,D,E), 83 tetraploid individuals (17.7%; Figure S4C,J,K,L), further, seven triploid specimens (1.5%; Figure S4F–I), and finally, also three pentaploids (0.6%; Figure S4M). Based on ambiguity in peak patterns, 85 individuals were classified as unknown (18.1%) since they presented multiple peaks that did not match with previously expected patterns (Figure S4N–R). However, when pooled together by species, their global profiles resembled the most common profile for that species. For example, together, the 43 *B. pumila* resembled a peak profile reminiscent of tetraploids (Figure S4P vs. S4C), while the 23 *B. glandulosa* resembled a diploid‐type profile (Figure S4N,O vs. S4A,B). Only the *B. nana* profiles seemed more complex to resolve. Altogether, 98% of individuals identified as *B. glandulosa* exhibited a diploid profile, while 93% of individuals identified as *B. pumila* showed a tetraploid profile (this percentage rises to 95% when including the 43 putative tetraploid *B. pumila* individuals).

**TABLE 4:** Number of individuals per ploidy level detected in the sample.

Ploidy level	Number of individuals
Diploid	291
Putative diploid	23
Tetraploid	83
Putative tetraploid	43
Others	10
Unknown	19
Total	469

*Note*: The category ‘Others’ refers to other ploidy level detected within the sampling and the category ‘Unknown’ refers to individuals with an unclear ploidy level.

Similar to the results obtained with the DAPC, STRUCTURE analyses on the diploid‐tetraploid dataset identified four to five different genetic clusters (Figure 1b, Figure S6). However, the Delta *K* statistic did not support four to five clusters, but rather identified *K* = 2 as the optimal number of clusters (Figure S7). In this case, the genetic clusters obtained at *K* = 2 illustrate the separation between the individuals from Alaska, Europe and Central‐Western Canada, and the second cluster regrouped the remaining *Betula* individuals from North America (Figure S6). We observed introgression from the eastern *B. glandulosa* group restricted to eastern Canada at *K* = 5 within the western *B. glandulosa* group (Figure 1b). Figure 1c illustrates the geographical distribution of each genetic cluster identified with STRUCTURE for *K* = 5 with the diploid‐tetraploid dataset. The *B. pumila* cluster was composed only of individuals identified as tetraploid or non‐diploid (Table S3). Unidirectional introgression from the western *B. glandulosa* cluster into the *B. pumila* cluster was also detected. Also, in Figure 1b, Alaskan individuals form a distinct group that appears to be admixed with western *B. glandulosa* individuals (putative hybrids). The individuals in the framed population (IZAAC, Figure 1b) did not belong to a single genetic group, as they appeared as trihybrids. At *K* = 5, a fifth genetic group was observed in this population. Similar results were found with the initial diploid dataset, and *K* = 2 was also identified as the optimal number of clusters according to the Delta *K* statistic (Figures S9 and S10).

### 3.2 Nuclear genetic diversity among genetic clusters and genetic lineages

In the next sections, we will use the term ‘genetic cluster’ to refer to the genetic groups obtained with the DAPC and the term ‘genetic lineage’ to refer to a specific genetic group in which individuals with intermediate group membership were removed (see Section 2.6). The observed heterozygosity (*H*_o_) was equal to or higher than the expected heterozygosity (*H*_e_) for all 74 populations of the initial diploid dataset (Figure S11). Consequently, the inbreeding coefficient was negative for all populations (Figure S12, Table S4). With respect to the heterozygosity within each genetic cluster, we found that heterozygosity was higher in the *B. pumila* group (Figure S13). Also, both groups of *B. glandulosa* (east; west) showed a similar level of heterozygosity while being the lowest among all groups (Figure S13). Heterozygosity within the Alaskan and European group was more variable than in the other four groups (Figure S13A), especially in the Alaskan lineage (Figure S13B).

**FIGURE 2:**
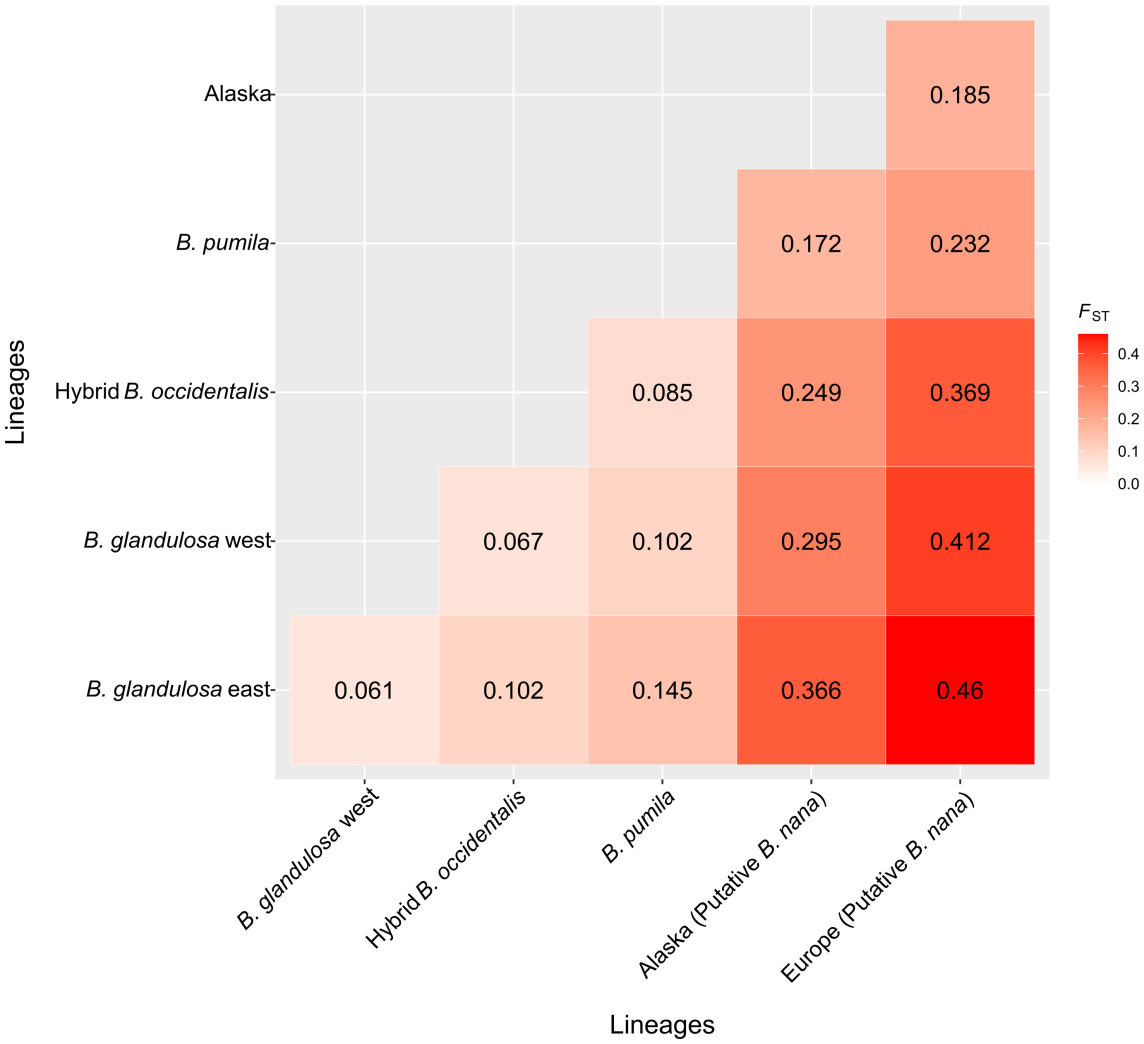
Pairwise *F*_ST_ between genetic lineages within the *Betula* sample.

Pairwise *F*_ST_ values estimated between genetic lineages are shown in Figure 2. The highest genetic divergence values were observed between the European lineage and the other North American birch lineages, especially with the *B. glandulosa* lineages (Figure 2, *B. glandulosa* east *F*_ST_ = 0.46 and *B. glandulosa* west *F*_ST_ = 0.41). Surprisingly, the European lineage diverged less from the Alaskan lineage (*F*_ST_ = 0.18) than from other North American lineages (0.23–0.46). Within North America, the lineage most divergent from the others was the Alaskan, with *F*_ST_ values ranging from 0.172 to 0.366 compared with the other groups. The group of potential hybrids with *B. occidentalis* was closest to the *B. glandulosa* western cluster. The pairwise *F*_ST_ values between the DAPC genetic clusters were similar to those between the genetic lineages (Figure S14).

### 3.3 Chloroplast DNA

**FIGURE 3:**
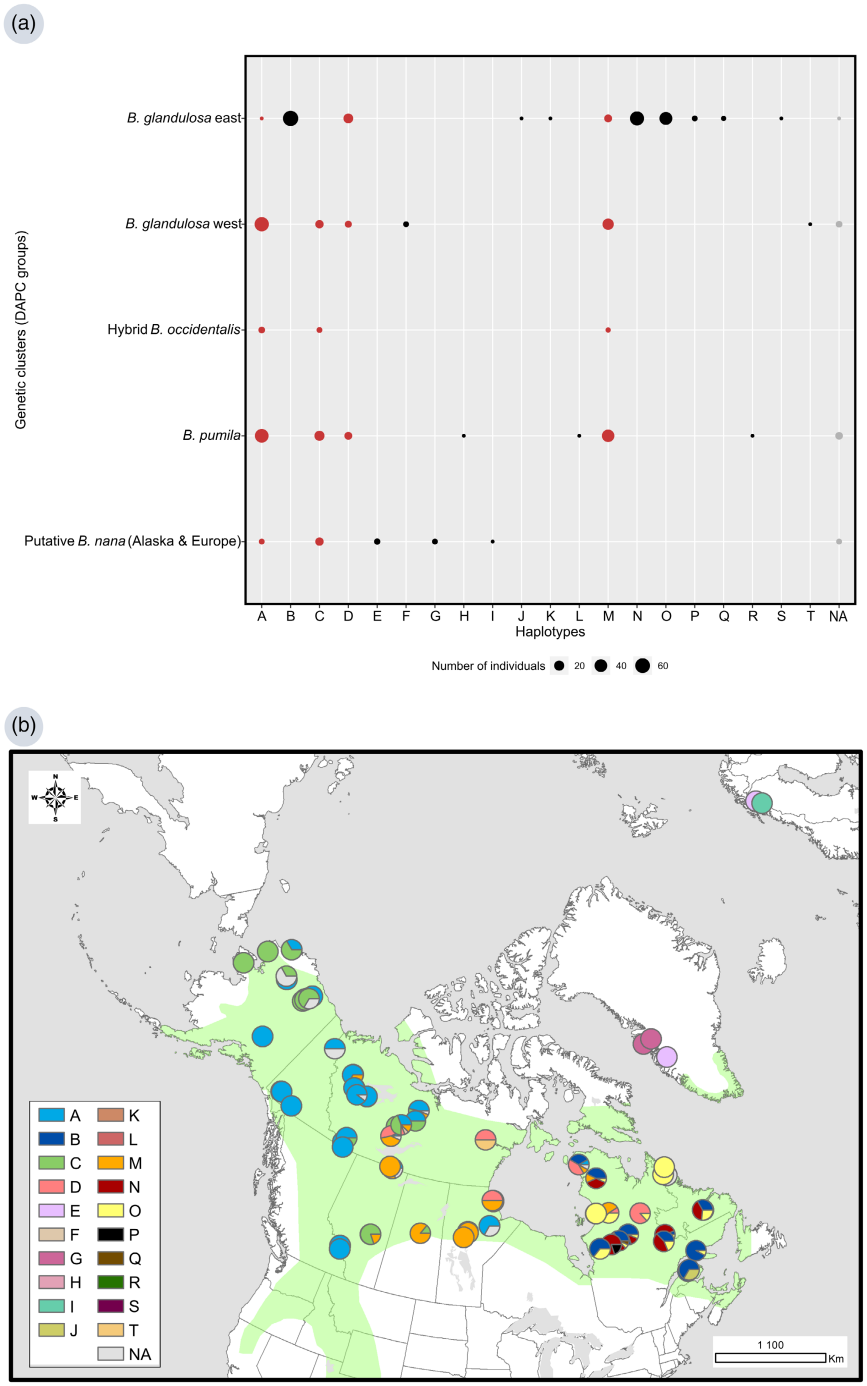
(a) Genetic cluster (DAPC groups) affiliations to each chlorotype. The size of the circle represents the abundance (in number of individuals) of each chlorotype. Red chlorotypes are shared between clusters while black chlorotypes are endemic to a single cluster. Individuals without chlorotype assignment are represented by NA. (b) Distribution of the chlorotype diversity among the sampled populations. Each haplotype has a different colour, and each pie chart corresponds to a population. The green area represents the distribution range of *Betula glandulosa* according to Furlow (1997).

At the cpDNA level, using 41 uniquely informative SNPs, we observed 20 different haplotypes across all genetic groups (Figure 3a,b). Some haplotypes were shared between genetic clusters and between the different birch species detected in our dataset. Haplotype A was shared among all North American clusters. In contrast to what was observed at the nuclear level, Alaskan and European individuals seem to be distinct at the cpDNA level and did not share haplotype M with the other lineages (Figure 3b). We observed a geographical distribution of haplotypes which did not fit the species boundaries. The highest haplotype diversity was observed in the eastern group of *B. glandulosa* where 11 different haplotypes were detected.

### 3.4 Morphology

A total of 309 leaf samples were analysed for taxonomic identification. Of these samples, 197 showed characteristics associated with *B. glandulosa* and 98 showed criteria typical of *B. pumila*. For most of the samples analysed, identification using leaf morphology criteria confirmed the results obtained from genomic data analyses (ploidy and population structure). In addition, 14 samples collected from the IZAAC population that presented a mixed genetic makeup according to STRUCTURE and DAPC results (see above) also showed intermediate morphological characters that were associated with *B. occidentalis* and *B. glandulosa* species (Figure S8). The most informative leaf morphology criteria were blade shape and teeth shape, while other criteria overlapped between species.

### 3.5 Sampling teams

**FIGURE 4:**
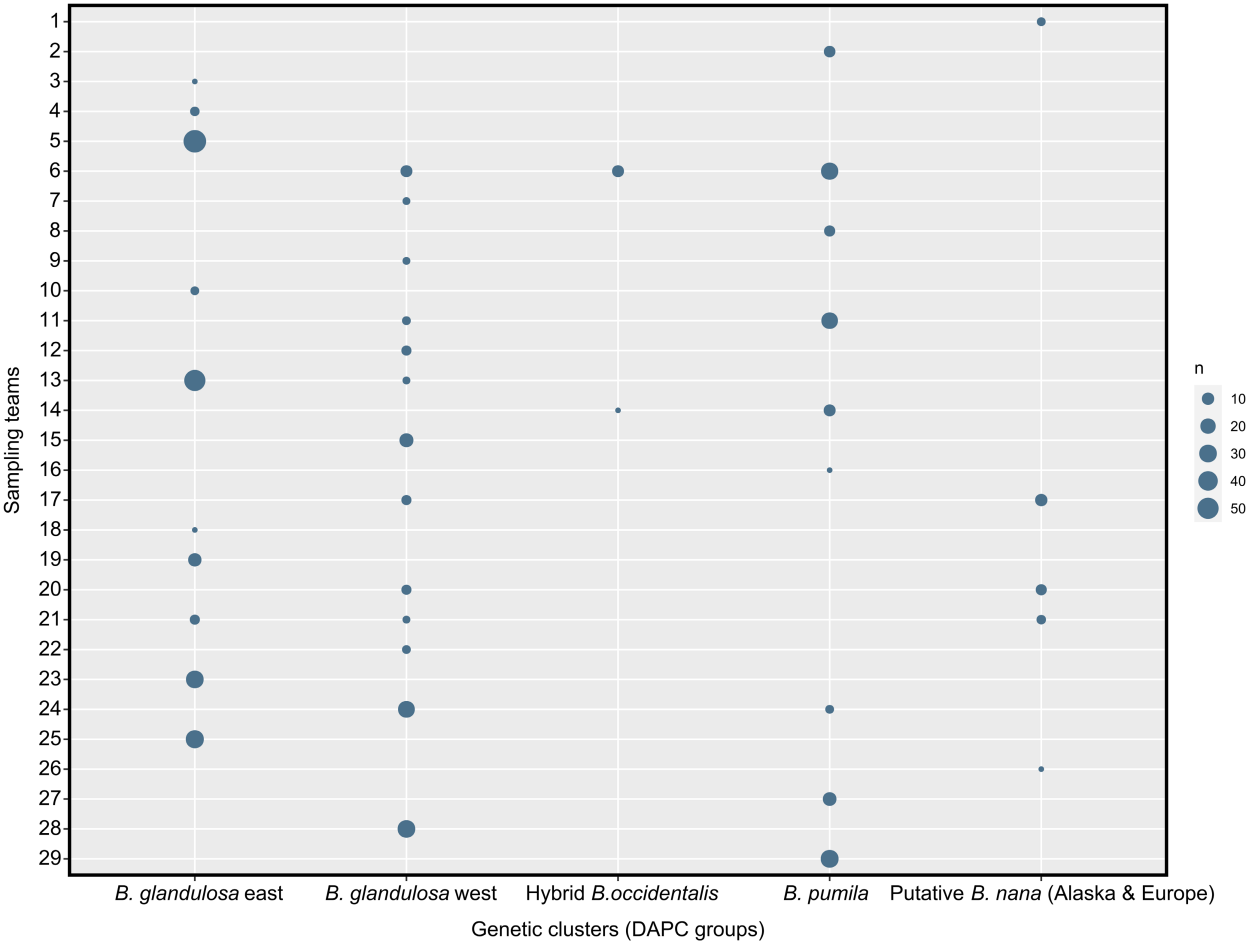
Affiliation of the sampling teams to the different Betula genetic clusters (DAPC groups) harvested. The size of each circle is proportional to the number of samples collected.

Looking at the affiliation of the sampling teams to the genetic clusters permitted us to observe that each genetic cluster (DAPC groups) was harvested by more than one team (Figure 4). The three most frequently sampled groups were the two clusters of *B. glandulosa* and the *B. pumila* cluster. Nearly one‐third (31%) of the teams (9/29 teams) collected at least one individual of *B. pumila*. Most of the teams collected only one genetic cluster, but sometimes different species than the one expected (Figure 4). However, some teams collected individuals from more than one group and, thus, different species. Nearly 50% (14/29) of the teams collected individuals that did not fall into the two *B. glandulosa* genetic groups.

## 4 DISCUSSION

Unlike other northern plant species that are experiencing range contractions in response to CC (Alsos et al., 2012; Parmesan, 2006), dwarf birch instead appears to be benefitting from rising temperatures by expanding its range. Our initial goal was to investigate the biogeography and genetic diversity of *B. glandulosa*, a North American low Arctic tundra dwarf birch. Taxonomic validation of dwarf birch is difficult as it is plastic and resembles other shrub birch species in sympatric zones. The use of a multicriteria approach reveals the presence of at least three different species and their hybrids among the 537 collected samples. Indeed, comparison of the nuclear and chloroplast SNP data highlights a long history of gene exchanges between the different taxa and lineages, which is in line with previous phylogeny studies of this genus (Wang et al., 2016, 2021). Together, our results underline that dwarf birch is possibly a component of a syngameon involving several other distinct related species of shrub birch.

### 4.1 Syngameon: A reproductive network of interconnected species that may facilitate adaptation under CC

Our results illustrate the complexity of assessing the genetic diversity of one species within a genus such as *Betula* that is characterized by a complex reticulate evolutionary history (Wang et al., 2021), and where extensive hybridization is likely present (Anamthawat‐Jónsson, 2019; Ashburner & McAllister, 2013; Bona et al., 2018; Ding et al., 2021; Tsuda et al., 2017; Zohren et al., 2016). Moreover, reliable morphological criteria are limited (Lepage, 1976; Payette, 2015) while the similarity of some traits, notably dwarfism, may be the product of convergent evolution (Li et al., 2005; Wang et al., 2021). Consequently, our sampling unintentionally included several species and hybrids, alongside the targeted species. This led to the uncovering of a possible birch syngameon through a multicriteria approach based on ploidy level, patterns of nuclear and chloroplast DNA variation, and, when possible, morphology (see Table 5). In Table 5, we summarize the discriminating criteria used to assign individuals to different birch species.

**TABLE 5:** Discriminatory potential (X) of each criterion used in the multicriteria approach to distinguish North American *Betula* spp. in the sample.

Discriminating criteria	Prima facie species			
	*B. glandulosa*	*B. pumila*	Hybrid *B. occidentalis*	Putative *B. nana* (Alaska–Europe)
Ploidy level	X	X	–	–
Clustering analyses on nuclear DNA diversity (DAPC and STRUCTURE)	X	X	X	X
*F*_ST_	X	X	–	X
Heterozygosity (Figure S13)	–	X	–	–
Chloroplast DNA haplotype diversity	–	–	–	–
Morphology	X	X	X	–

Hybridization and introgression phenomena have been recognized as evolutionary processes that facilitate and enhance the adaptation of plant species to changing environments (Abbott et al., 2013; Stebbins, 1959). Indeed, a syngameon is a reproductive network of three or more distinct related species connected by limited gene flow and interacting over large time and space scales, allowing constituent species to persist (Boecklen, 2017; Buck & Flores‐Rentería, 2022; Cannon & Petit, 2019). Several syngameons have been documented in plants (Buck & Flores‐Rentería, 2022), especially the oak syngameon (Cannon & Petit, 2019; Hipp et al., 2019). In the genus *Betula*, the first birch syngameon was discovered by Gunnarsson in 1925 in Scandinavia, consisting of six species: *B. verrucosa*, *B. coriacea*, *B. concinna*, *B. pubescens*, *B. tortuosa* and *B. nana* (Lotsy, 1925). Another birch syngameon was found following the discovery of *B. murrayana* in southeastern Michigan encompassing four species: *B. alleghaniensis*, *B. pumila*, *B*. x *purpusii* and *B. murrayana* (Barnes & Dancik, 1985). In our study, observed patterns of nuclear and chloroplast DNA variations suggest that *B. glandulosa* could be a core participant in a possible syngameon of North American shrub birch species. Without mentioning the word syngameon, Lepage (1976) evoked the idea of an interconnected network of species and hybrids in North American birches: ‘A continuous series of specimens, from *B. nana* to *B. papyrifera*, could easily be found, connecting by intermediates each species with its neighbor’. Population structure analysis revealed the presence of hybrids of *B. glandulosa* with at least two other birch species (*B. occidentalis* and putative *B. nana*) and introgression of the western cluster of *B. glandulosa* into the tetraploid *B. pumila* cluster. Finally, the observed chloroplast capture (chlorotypes shared by two species or more) is also evidence of gene exchange between species. Altogether, these results imply that the sampled species have shared some of their genetic makeup in the past and still exchange alleles through ongoing gene flow and hybridization, thereby pointing towards a syngameon. We propose that these species form a fraction of a more complex network of interconnected species, a more complex syngameon. Further evidence will be needed to describe the extent of this syngameon and its constituents as our sampling was limited and our study was not initially designed to investigate the syngameon. A study with the primary focus of understanding this birch syngameon will be able to determine the number of species forming this syngameon and the relationships between them. The next sections will highlight the supporting elements gathered in our study suggesting the existence of a North American birch syngameon.

#### 4.1.1 One or several species?

The multicriteria approach employed in this study revealed the presence of at least three distinct related species within the sampling area and allowed the detection of five genetic groups overall. The identification of *K* = 2 as the optimal number of clusters with STRUCTURE using the ∆*K* method suggests that there is a hierarchical structure in the dataset. This may be explained by the presence of more than one species in the dataset. The Δ*K* method has been commonly criticized for its tendency to predominantly recognize the highest level of genetic structure, especially when dealing with data that exhibit hierarchical structures. This aspect is well‐documented as a limitation of the method and has prompted recommendations to exercise caution and not place excessive reliance on it (Cullingham et al., 2020; Janes et al., 2017). Therefore, a combination of criteria (ploidy, morphology and geography) has also helped determine the most realistic number of *K* within the sampling, which is five groups. In addition to two intraspecific lineages of *B. glandulosa*, two other groups were particularly more divergent from the others and corresponded to other species: the *B. pumila* and the putative *B. nana* clusters. Finally, a genetic group of hybrid origin with *B. occidentalis* was identified.

Our results indicate the presence of *B. pumila* in the sampling. Because of their similar morphological characteristics (twigs and leaves), *B. pumila* can be easily confounded in the field with *B. glandulosa* (Ashburner & McAllister, 2013; Furlow, 1997; Lepage, 1976; Payette, 2015). Within the genus *Betula*, the ploidy level is known to vary among species from diploid to dodecaploid, and about 60% of birch species are polyploid (Wang et al., 2016). However, *B. pumila* is the only tetraploid birch species in North America (Ashburner & McAllister, 2013). Looking at the ploidy of each sampled individual revealed the occurrence of mainly diploid and tetraploid birches, leading to the conclusion that more than one species was collected, one of them being *B. pumila*. The group identified as *B. pumila* had a higher percentage of heterozygosity than the *B. glandulosa* groups, which could be expected since heterozygosity is generally higher within polyploid species (Soltis & Soltis, 2000). For a more comprehensive understanding of the syngameon and its components, exploring *B. glandulosa*'s potential role as a diploid progenitor of *B. pumila* would be worthwhile, considering that Wang et al. (2021) recently studied diploid progenitors contributing to polyploid *Betula* species genesis, but excluded *B. glandulosa* for technical reasons.

The results obtained for population structure analyses led to the identification of a second species in the sampling other than the focal species, the putative *B. nana*. This genetic cluster corresponds to individuals sampled in Europe and Alaska. The *B. nana* complex consists of two subspecies: *B. nana* ssp. *nana* and *B. nana* ssp. *exilis*. *B. nana* ssp. *nana* is a widespread circum‐Arctic shrub birch species distributed throughout Europe from Greenland to Siberia (Ashburner & McAllister, 2013; de Groot et al., 1997), while, according to Furlow (1997), *B. nana* ssp. *exilis* could be found in the northern regions of western North America and across Siberia and is morphologically very similar to *B. glandulosa*. The pairwise *F*_ST_ between the genetic clusters confirmed that Alaskan and European individuals form highly divergent lineages compared with other North American lineages. The extent of the *B. nana* complex could explain why Alaskan (potentially *B. nana* ssp. *exilis*) and European (potentially *B. nana* ssp. *nana*) individuals belong to the same STRUCTURE and DAPC cluster. However, this hypothesis goes against the proposition of Ashburner and McAllister (2013) in the most recent taxonomic revision of the genus *Betula*, who considered that *B. nana* ssp. *exilis* is a variety of *B. glandulosa*. This proposition was based only on morphological characteristics (Ashburner & McAllister, 2013). Later, and based on nuclear DNA markers, Eidesen et al. (2015) analysed the genetic structure of *Betula* samples, including both *B. nana* subspecies, *B. pubescens* and *B. glandulosa*. Their results did not corroborate the classification proposed by Ashburner and McAllister (2013). It is therefore plausible that *B. nana* ssp. *exilis* and *B. glandulosa* could form distinct genetic groups at the nuclear DNA level.

#### 4.1.2 Evidence of hybridization and introgression

Our results show that hybridization is relatively frequent among *Betula* shrub species in North America. In addition to the two lineages of *B. glandulosa* and the two congeneric species detected, a group of recent hybrids with *B. occidentalis* was identified. These individuals were collected in a population of the Northwest Territories. *B. occidentalis* is a diploid shrub birch, just like *B. glandulosa*, and is found in western North America (Ashburner & McAllister, 2013). Hybridization between *B. occidentalis* and *B. glandulosa* is possible where their ranges overlap (western North America; Ashburner & McAllister, 2013; Dugle, 1966; Furlow, 1997), which is coherent with the location of the sampled individuals. The individuals did not fall into any particular STRUCTURE group and appeared as trihybrids belonging to three different genetic clusters (eastern and western *B. glandulosa* clusters and a new cluster; Figure S9). Leaf morphology and pictures of individuals confirmed the presence of *B. occidentalis* hybrids that were not detected through the ploidy level assessment (Table 5, Figure S8). This hybrid population is indirect evidence of the presence of a fourth species within the possible shrub birch syngameon. In sympatric zones, the STRUCTURE analysis also revealed evidence of recent hybridization between the western *B. glandulosa* cluster and the putative *B. nana* cluster, as well as unidirectional introgression from the western *B. glandulosa* cluster within the *B. pumila* cluster (Figure S9). Gene exchange and unidirectional introgression from *B. glandulosa* into *B. pumila* were already reported in the literature (Brunsfeld & Johnson, 1986; de Groot et al., 1997; Dugle, 1966).

We also found evidence of frequent hybridization and gene exchange between related birch species through chloroplast capture. The analysis of cpDNA at the sampling scale showed that cpDNA diversity is geographically distributed and overcomes species boundaries. Indeed, many chlorotypes were shared by more than one species and genetic cluster. The chloroplast capture phenomenon was also observed within the genus *Betula* in birch trees of eastern North America (Thomson et al., 2015) and shrub birches and trees of Europe (Eidesen et al., 2015; Maliouchenko et al., 2007; Palmé et al., 2004; Thórsson et al., 2010). Thomson et al. (2015) studied the phylogeographical structure of temperate and boreal birch tree species in eastern North America. They concluded that haplotype sharing between *Betula* tree species would be better explained by punctual hybridization phenomena followed by introgression, rather than a common biogeographical history. This historical haplotype sharing may nevertheless explain why the reproductive barriers between species of the genus *Betula* remain permeable and blurred today, which is observed at the nuclear level with hybridization and introgression, and which supports the hypothesis of a syngameon composed notably of North American shrub birches. Chloroplast capture has been documented on several occasions in the scientific literature (Rieseberg & Soltis, 1991) and has been reported in other species and genera of angiosperms such as *Populus deltoides* (Godbout et al., 2019), *Quercus* spp. (Petit et al., 1993; Whittemore & Schaal, 1991) and *Salix* spp. (Percy et al., 2014; Twyford, 2014). Our results also illustrate that cpDNA was not a key discriminating factor in identifying congeneric *Betula* shrubs in the sampling (Table 5), raising an issue of species identification based on plastid DNA alone. This challenge has already been discussed by Percy et al. (2014) and Twyford (2014) for *Salix* species, a genus where interspecific hybridization also frequently occurs.

Disequilibrium and disturbances such as CC could favour plant hybridization (Anderson, 1948; Chunco, 2014; Rieseberg & Carney, 1998; Todesco et al., 2016) and the formation or reinforcement of syngameons (Buck & Flores‐Rentería, 2022). CC is already altering flowering phenology and growing season duration in cold regions, and therefore reproductive barriers (Cleland et al., 2007), as well as distribution ranges (Parmesan, 2006; Parmesan & Yohe, 2003). These temporal and spatial shifts together may create overlaps between related species and secondary contacts between allopatric species, leading to new hybridization opportunities (Chunco, 2014). Currently, dwarf birches appear to benefit from warming temperatures by expanding their range (Myers‐Smith et al., 2011). Shrub expansion by *B. glandulosa* could be a trigger for the birch syngameon by allowing connections between closely related species occupying similar environments. Therefore, new opportunities for hybridization between dwarf birch and other *Betula* species may arise, necessitating the consideration of congeneric birch species when sampling. Hybridization and connections established between species forming a syngameon can also be seen as mechanisms to maintain certain alleles of cold‐adapted arctic and subarctic species that could be facing extinction due to CC (Buck & Flores‐Rentería, 2022; Charles & Stehlik, 2021). Thus, these adaptive strategies of dwarf birch may help maintain some of its genetic makeup over time. More frequent hybridization and a larger reproductive network will represent an additional challenge to species identification for sample collectors who will be dealing with greater morphological variability (Floate et al., 2016).

**FIGURE 5:**
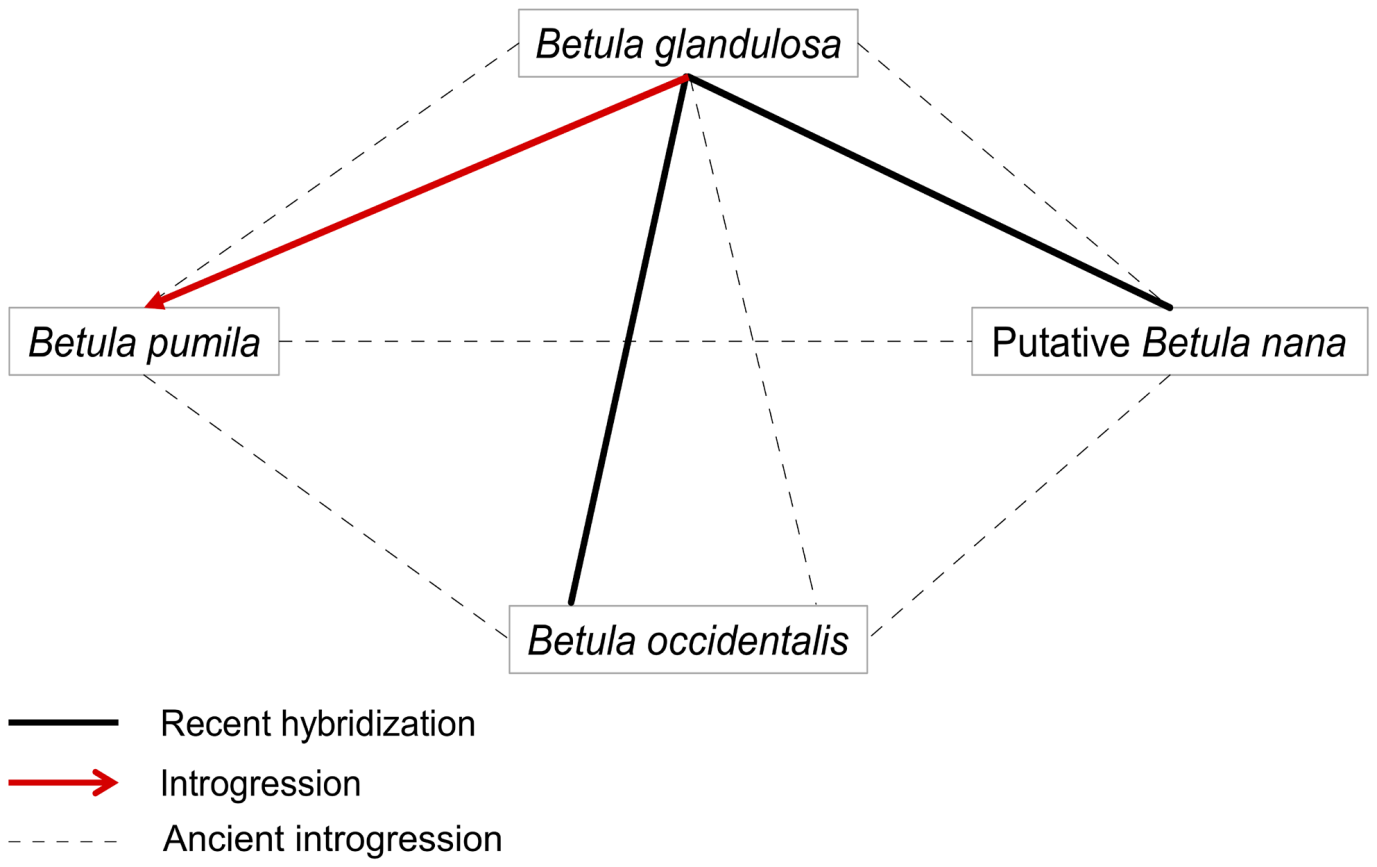
Representation of the North American shrub birch syngameon revealed through vast geographical sampling encompassing four species: *Betula glandulosa*, *B. pumila*, putative *B. nana* and *B. occidentalis*. Evidence of recent hybridization between species is represented by a full black line, introgression direction is illustrated by a red arrow while more ancient introgression is shown by a dotted black line.

Figure 5 is a graphical representation of the syngameon observed in this study based on the evidence we have accumulated, that is the detection of multiple species (*B. glandulosa*, *B. pumila*, putative *B. nana* and a hybrid with *B. occidentalis*) and evidence of recent and past hybridization between them.

### 4.2 *Betula glandulosa*: The core of our network

One of the main characteristics of a syngameon is the ability of its component species to remain distinct despite occasional gene exchanges (Cannon & Petit, 2019). Our study highlights that dwarf birch appears to maintain its own identity over time, even as part of a reproductive network of interconnected birch species. Within *B. glandulosa* species, we detected a geographical population structure in North America corresponding to two genetic clusters: an eastern cluster and a western cluster that likely correspond to two different glacial lineages. A study on plant macrofossils in eastern North America reported the presence of shrub birches and other birch trees at the southeast margin of the glacier from 18 kya ^14^C BP (Jackson et al., 1997). Pollen and plant macrofossils indicate that *B. glandulosa* was found along the edge of the Laurentide Ice Sheet in the eastern provinces of Canada at the end of the ice age 10 kya. It then took advantage of climatic variations to spread by moving northwards into the provinces of Quebec and Labrador following deglaciation (Gajewski et al., 1993; Levesque et al., 1994; Richard et al., 1992, 2020).

Plant macrofossils and high haplotype diversity observed in the eastern *B. glandulosa* group suggest that this genetic cluster probably originated from a glacial refugium in eastern North America at the southern margin of the glacier (Jackson et al., 1997; Jaramillo‐Correa et al., 2009), while the second *B. glandulosa* group probably came from a refugium located in western North America. However, it is not possible to determine from which glacial refugium these individuals originated, whether the Beringia refugium (Abbott & Brochmann, 2003) or another one, even if there was pollen evidence of the presence of dwarf shrubs of the *B. glandulosa*/*nana* complex in the Beringia and Alaska regions since the last glacial maximum (Anderson & Brubaker, 1994; Naito & Cairns, 2011). Nevertheless, the geographical distribution of haplotypes supports the idea that *B. glandulosa* individuals came from at least two glacial refugia. The low sampling coverage of *B. glandulosa* in the centre of Canada due to the disproportionate sampling of *B. pumila* in this region does not allow us to confirm the presence of a third possible refugium in this area.

The geographical distribution of the organelle haplotypes in this study is similar to those reported in studies on conifers (mtDNA and cpDNA) that have a wide distribution area across North America like *Pinus banksiana* (Godbout et al., 2005), *Picea mariana* (Gérardi et al., 2010), *Picea glauca* (de Lafontaine et al., 2010) and *Larix laricina* (Napier et al., 2020; Warren et al., 2016). As for *B. glandulosa*, higher haplotype diversity and rare haplotypes are found for black spruce in eastern provinces (Gérardi et al., 2010). A study on a circumboreal plant species, *Chamaedaphne calyculata*, also identified a greater number of haplotypes in eastern North America than in the west (Wróblewska, 2012). The high haplotype diversity of *B. glandulosa* in eastern North America and the presence of rare haplotypes suggest that population size must have been quite large in the east, and therefore, genetic drift randomly eliminated fewer haplotypes. The introgression of the eastern cluster of *B. glandulosa* in the western cluster and the low *F*_ST_ value between both *B. glandulosa* groups suggest that there is still gene exchange between populations from the two genetic clusters.

### 4.3 Challenges of studying northern species involved in a syngameon and implications for conservation

One of the main challenges of working with a transcontinental subarctic species is conducting range‐wide sampling, especially in remote areas where species' ranges overlap. The most time‐efficient way to collect samples is by using a multi‐team sampling strategy that, in our case, might have likely exacerbated the possibility of sampling species other than the intended one. In fact, nearly half of the teams (14/29) sampled individuals that did not fall within any *B. glandulosa* groups. Several factors may have affected sampling. First, multiple teams consisting of researchers, students or people working in governmental organizations such as parks or natural resources departments and who were not experts in *Betula* taxonomy, but not neophytes either, were involved in the collection of samples. Due to territory accessibility issues, the teams were multi‐tasked and had limited time to collect samples for various research projects. This was especially true for some of the northern sites that were only accessible by helicopter. As mentioned earlier, hybridization and introgression have been frequently observed within the genus *Betula* (Ashburner & McAllister, 2013), which can rapidly complicate the identification of samples with unreliable morphological criteria. When working with boreal and subarctic shrubs, there is no guarantee that only one species will be sampled even if a team of taxonomic experts is hired to sample a species. Indeed, in a study focusing on *Vaccinium angustifolium*, experts in *Vaccinium* taxonomy conducted the sampling and still collected two to three different species and their hybrids (Godbout et al., in prep.).

In the context of current changes, the arctic and subarctic regions are among the most affected, which is a reminder of the importance of studying species that occur in these ‘at risk’ areas (Colella et al., 2020). However, as the issues and studies are becoming broader in scope, some sampling constraints are immutable: taxonomic challenges complexifying species identification, the extent of the territory, and budgets to sample species in these remote areas. In the future, the only way to achieve large‐scale sampling and have access to a large number of samples for genomic studies will be through the participation of multiple sampling teams, or even through participatory and citizen science (Dickinson et al., 2010; Silvertown, 2009). The quality and accuracy of sampling done by teams of volunteers with different levels of expertise can be similar to that of professionals (Kosmala et al., 2016). However, verification and validation methods to ensure the quality of sample identification are required and can take several forms (Kosmala et al., 2016). In remote areas, it would be beneficial to ask local northern communities to participate in the sampling. Many of them are Indigenous communities. Involving them in the sampling and developing partnerships requires a long‐term commitment, an adequate source of funding, and above all, close and transparent collaboration that considers the needs, values and knowledge of these communities (Touchette et al., 2021).

Our experimental design was not initially developed to use a multicriteria approach. Therefore, when working with species with blurred boundaries and overlapping ranges, with similar morphological criteria and sharing common adaptive strategies, we suggest planning an integrative approach at the initial stage of the project. Indeed, increased sampling efforts are needed to achieve a better resolution at the species level. As a sampling strategy, one should consider including transects in contact zones when they are known to gain crucial information for distinguishing intraspecific variation from interspecific variation (Chambers et al., 2022), as newly adapted individuals may emerge from these zones. Most importantly, when assessing genetic diversity and adaptive capacity of a species like dwarf birch, congeneric species should not be overlooked as they may have contributed to its sustainability over time. Based on the results obtained, whether sampling is done by a single team or multiple teams, a multicriteria approach combining complementary methods should always be advocated when working with species with overlapping ranges and likely to hybridize. In this context, the approach retained in the present study aims at raising awareness among researchers or people working with ‘recent’ species and syngameons to adopt a more vigilant attitude when identifying species within a sample.

Finally, our results indicating the possible presence of a syngameon as an adaptive strategy for North American shrub birches may have implications for future conservation strategies of organisms that rely partly on this coping strategy. For hundreds to thousands of years, species within a syngameon have occasionally hybridized and introgressed to maintain themselves on the landscape in periods of stability and rapid change. As an adaptive advantage, the syngameon can provide evolutionary flexibility and resilience that exceeds the capacity of its constituent species taken separately (Cannon & Petit, 2019). Conservation practitioners as well as policy makers should therefore consider the potential of evolutionary plasticity conferred by syngameons and extensive hybridization in the selection and implementation of conservation strategies (vonHoldt et al., 2017). In this regard, we would like to foster reflection on taboo conservation strategies, such as adaptive introgression, as they might be key in the future to facilitate species adaptation under CC (Hamilton & Miller, 2016; Heuertz et al., 2023; Suarez‐Gonzalez et al., 2018; Tigano & Friesen, 2016).

## CONFLICT OF INTEREST STATEMENT

The authors have no conflicts of interest to declare.

## Supporting information


Appendix S1.

Data S1.

## Data Availability

Data for this study are available in supplementary material.
