# Supplementary material for: A cryptic syngameon within Betula shrubs revealed: Implications for conservation in changing subarctic environments
Source: Evol Appl. 2024 Apr 17;17(4):e13689. doi: 10.1111/eva.13689 (PMC11022622; doi:10.1111/eva.13689)
Supplement: Supplementary file 1 — Appendix S1. [file EVA-17-e13689-s001.docx]

## Supplementary material

### Supplementary figures


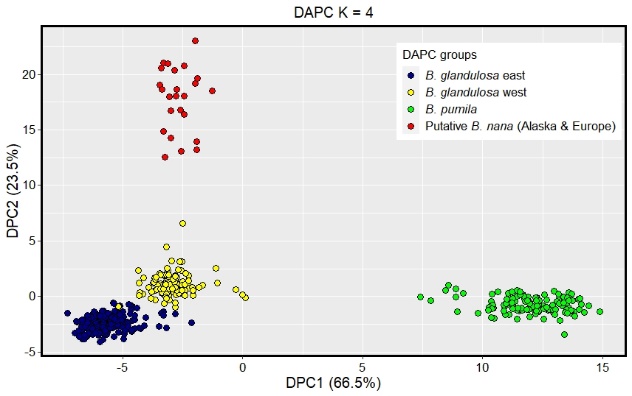

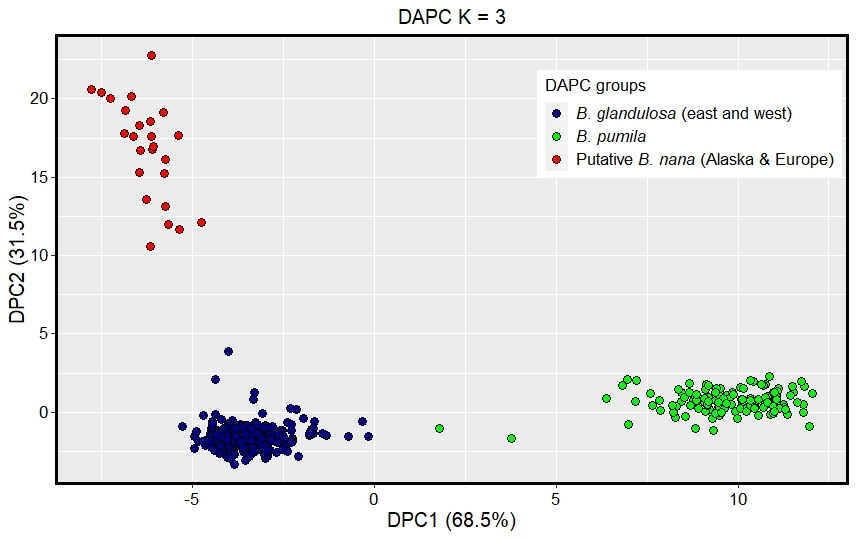

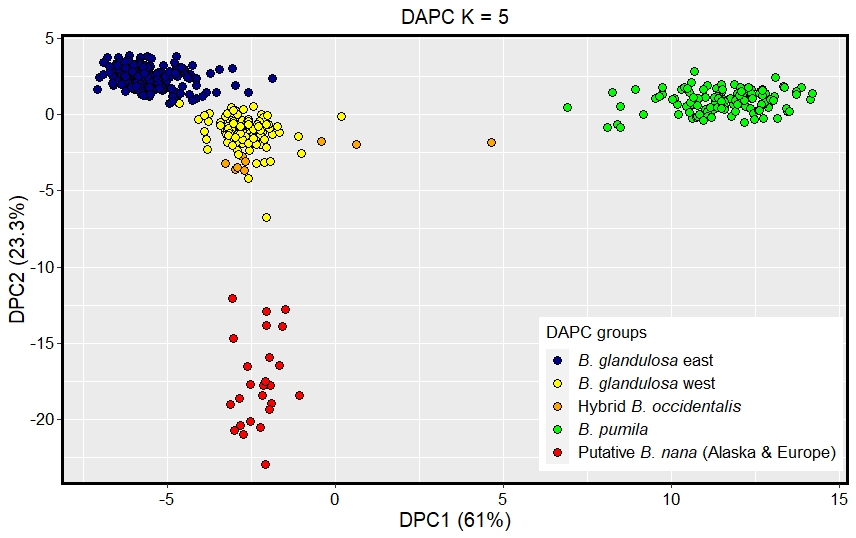

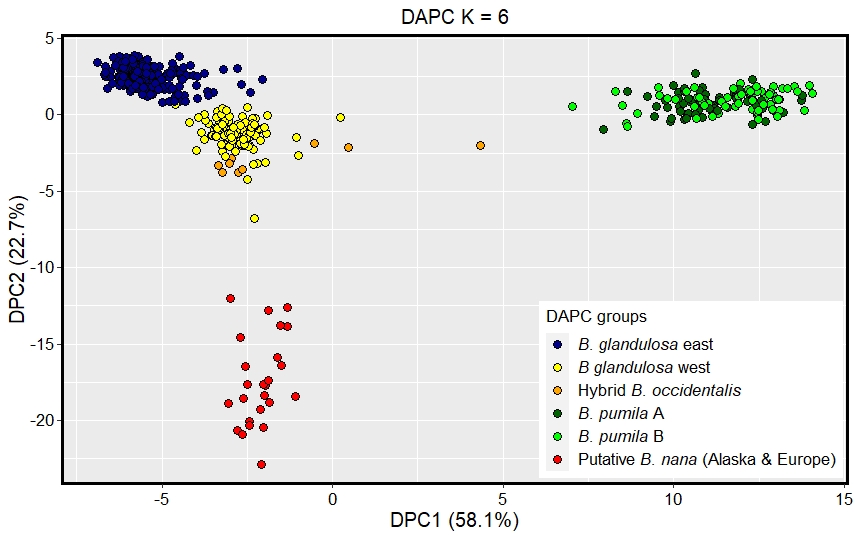

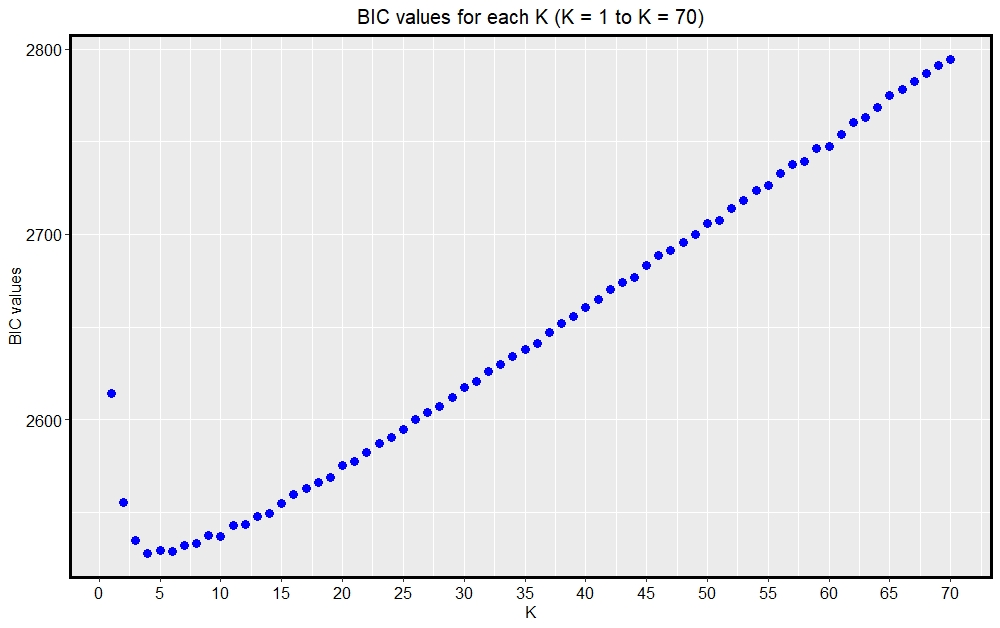

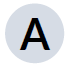

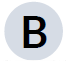


###### **Figure S1.** (a) Bayesian information criterion (BIC) values for determining the number of informative groups in the DAPC. (b) Discriminant analysis of principal components (DAPC) scatterplots for the initial diploid dataset for K = 3 to K = 6.


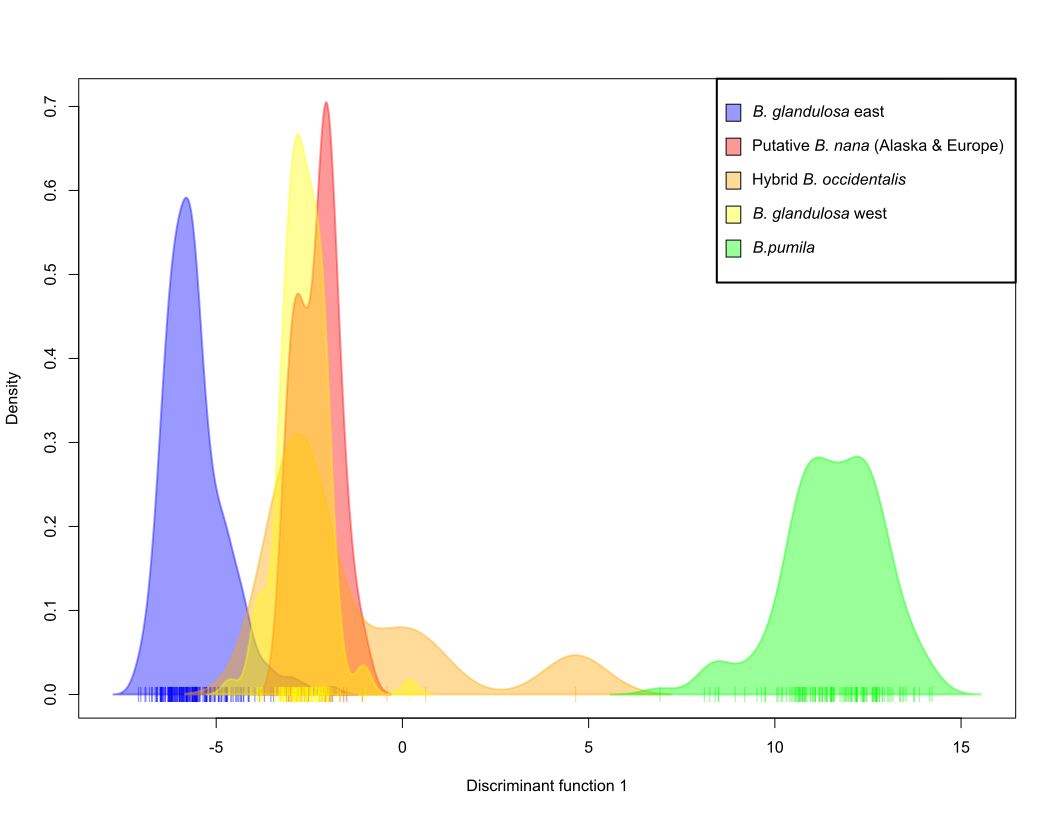


###### **Figure S2.** First axis of the DAPC performed on the initial diploid dataset of Betula spp.


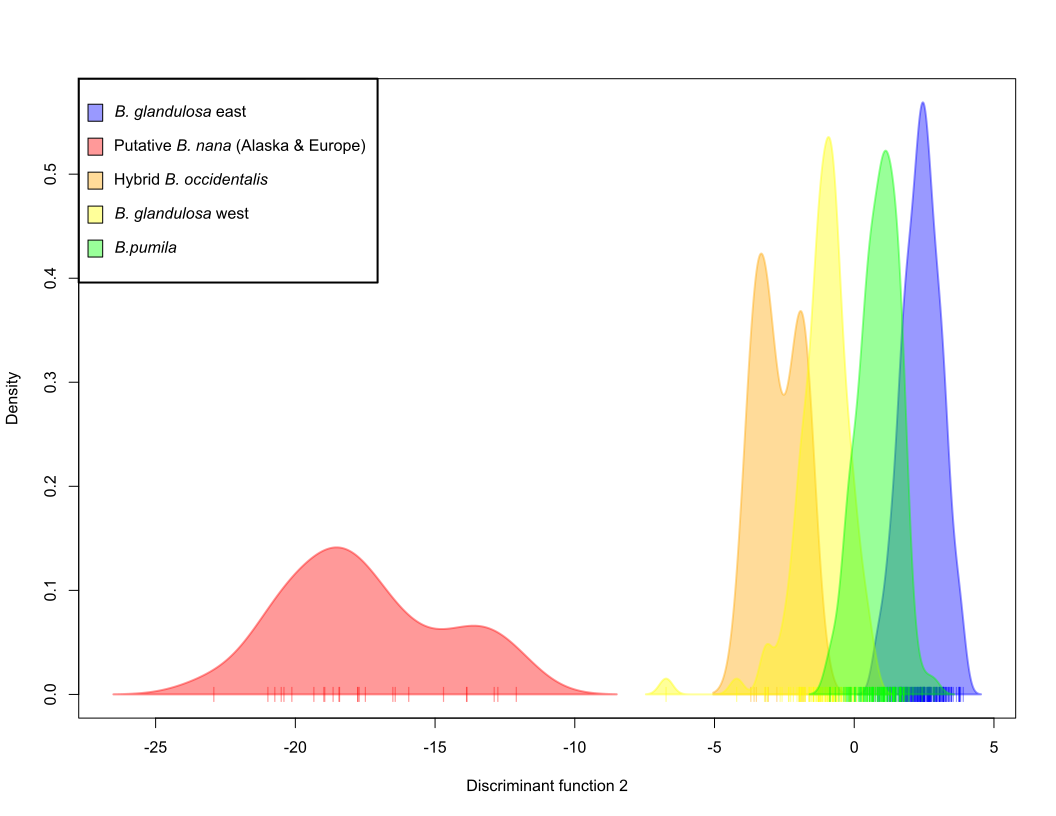


###### **Figure S3.** Second axis of the DAPC performed on the initial diploid dataset of Betula spp.


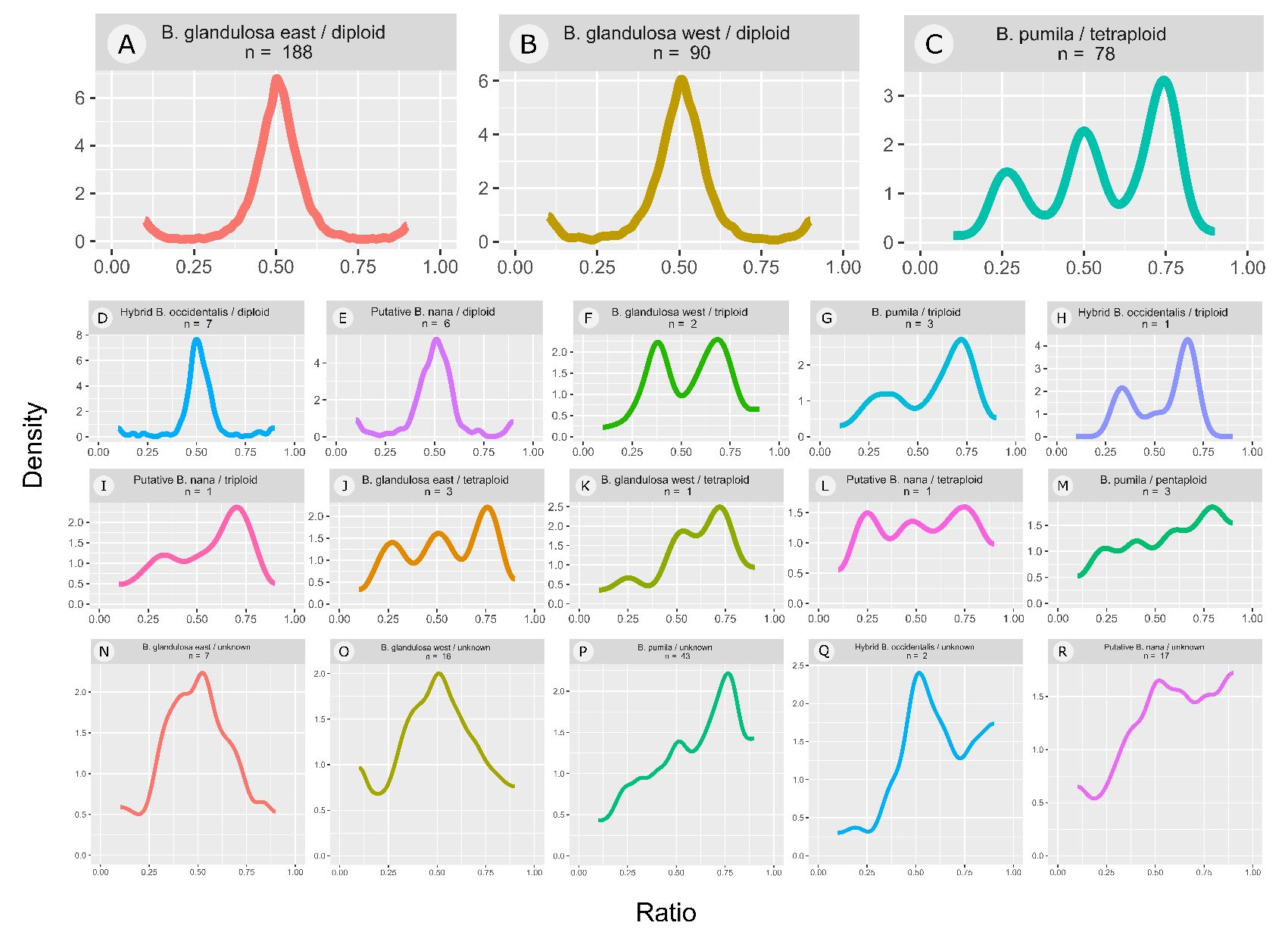


###### **Figure S4.** Determined ploidy level by species through allelic ratios density profiles. The profiles are the pooled result of n individuals with highly similar profiles. A, B and C are the most predominant groups: A and B are diploid B. glandulosa individuals and C represents tetraploid B. pumila individuals. D to M correspond to other less dominant ploidy profiles observed within the sampling. N to R correspond to the pooled allelic ratio density profiles per species for individuals with an unclear ploidy profile (unknown).


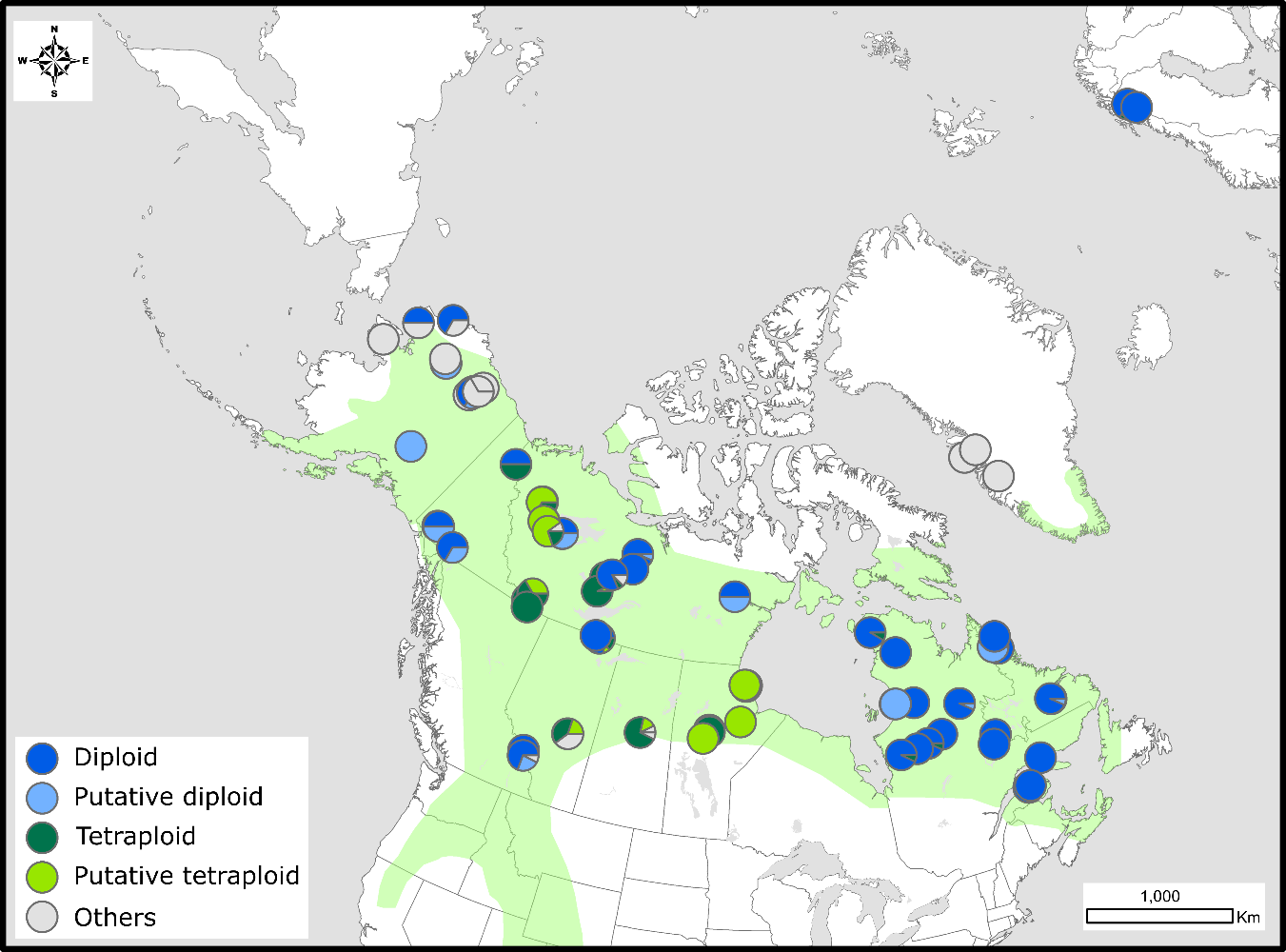


###### **Figure S5.** Geographical distribution of the ploidy level in the shrub birch population sample. Putative diploid and putative tetraploid individuals were identified based on allelic ratio density profiles pooled by species. The category “Others” refers to other ploidy level detected within the sampling and individuals with an unclear ploidy level. The green area represents the B. glandulosa distribution range according to Furlow (1997).

######
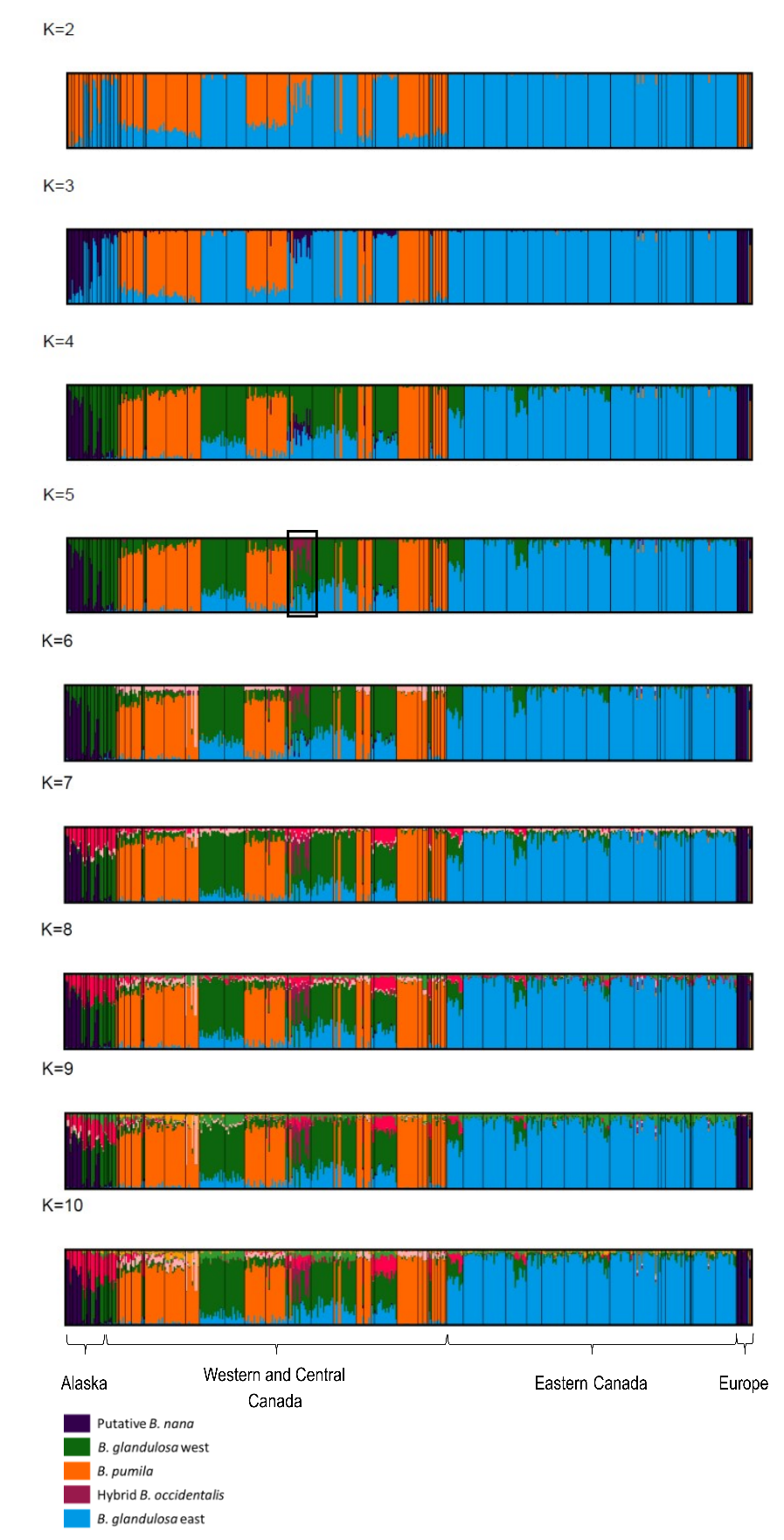
**Figure S6.** Structure barplots for the diploid-tetraploid dataset for K = 2 to K = 10. Individuals are represented following a longitudinal gradient: on the far left, individuals from Alaska and on the far right, individuals from Eastern Canada and Europe. The rectangle indicates a population of hybrid individuals detected from K = 5.


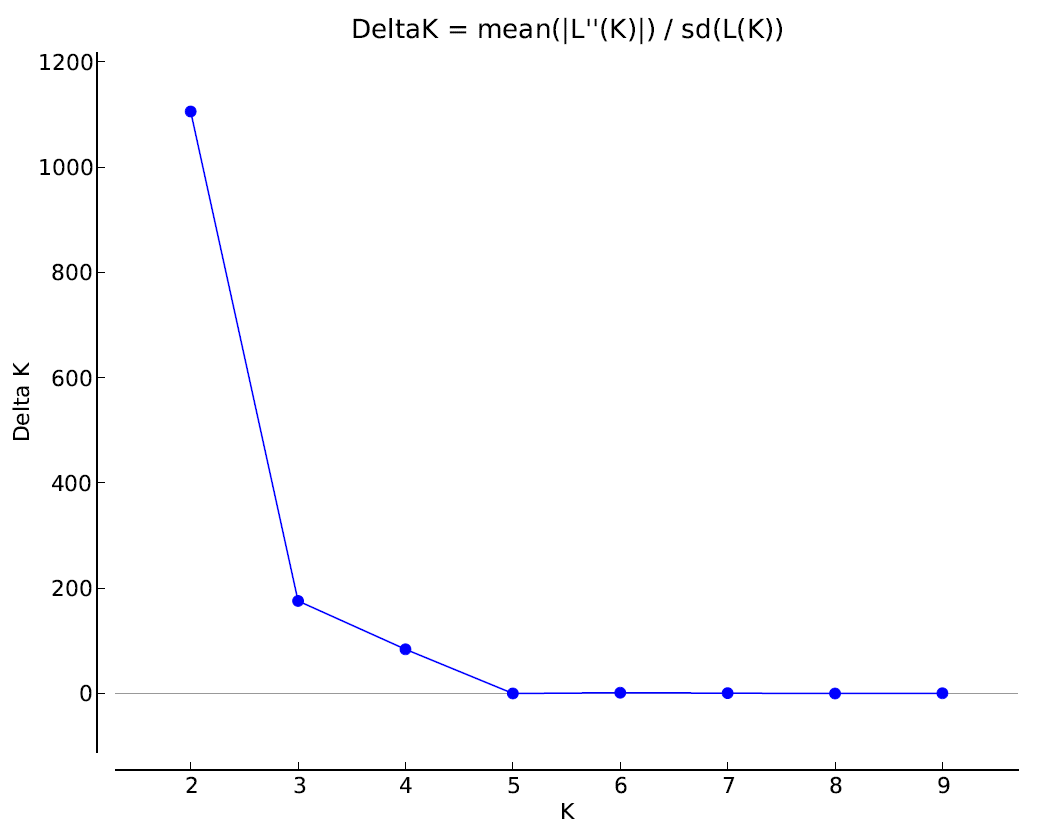


###### **Figure S7.** Delta K vs K (Evanno et al., 2005) for the STRUCTURE analysis using the diploid-tetraploid dataset. The plot was constructed using Structure Harvester (Earl and vonHoldt, 2012). Arrow indicates the inferred number of clusters.

*B. glandulosa*


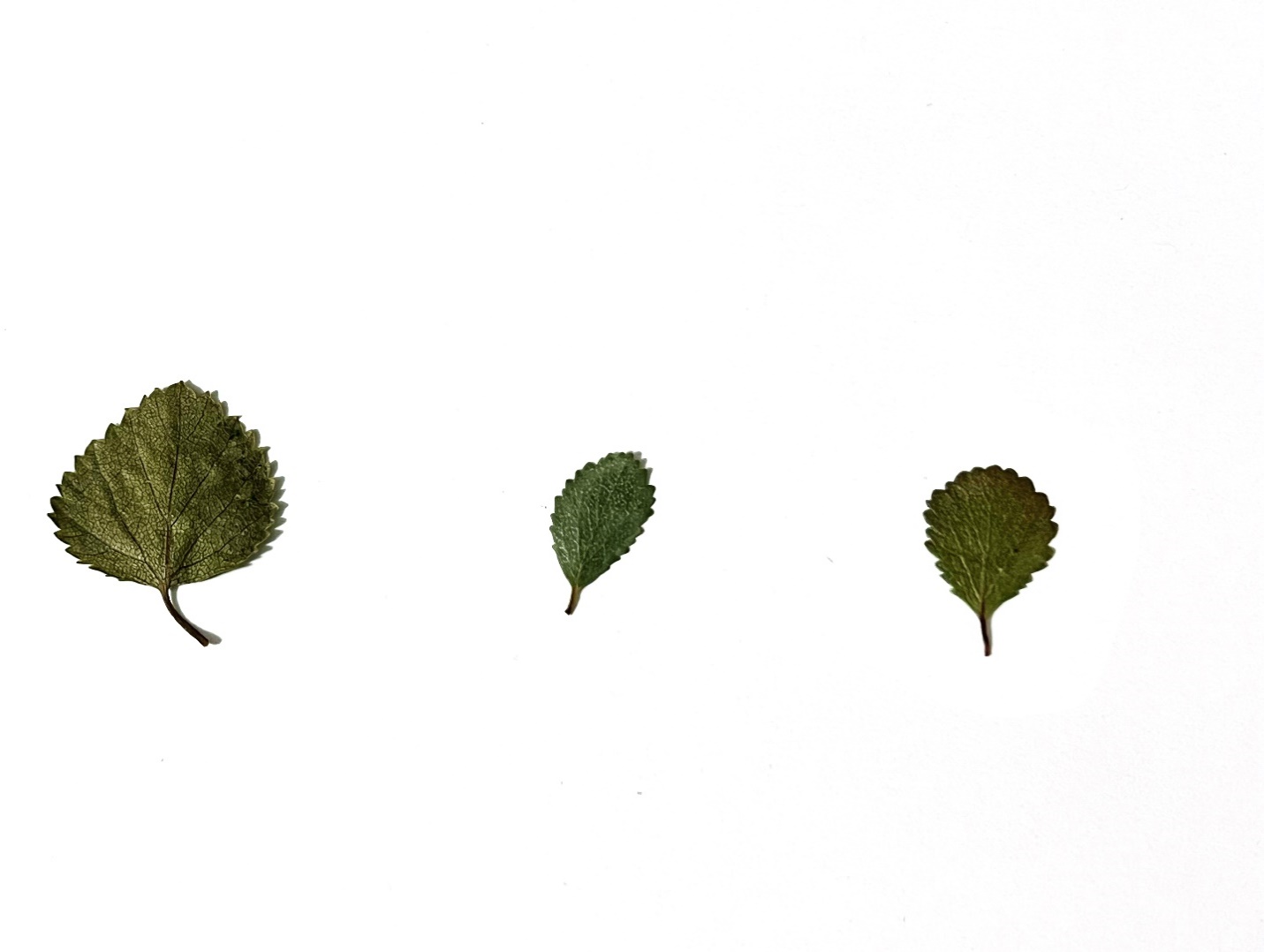

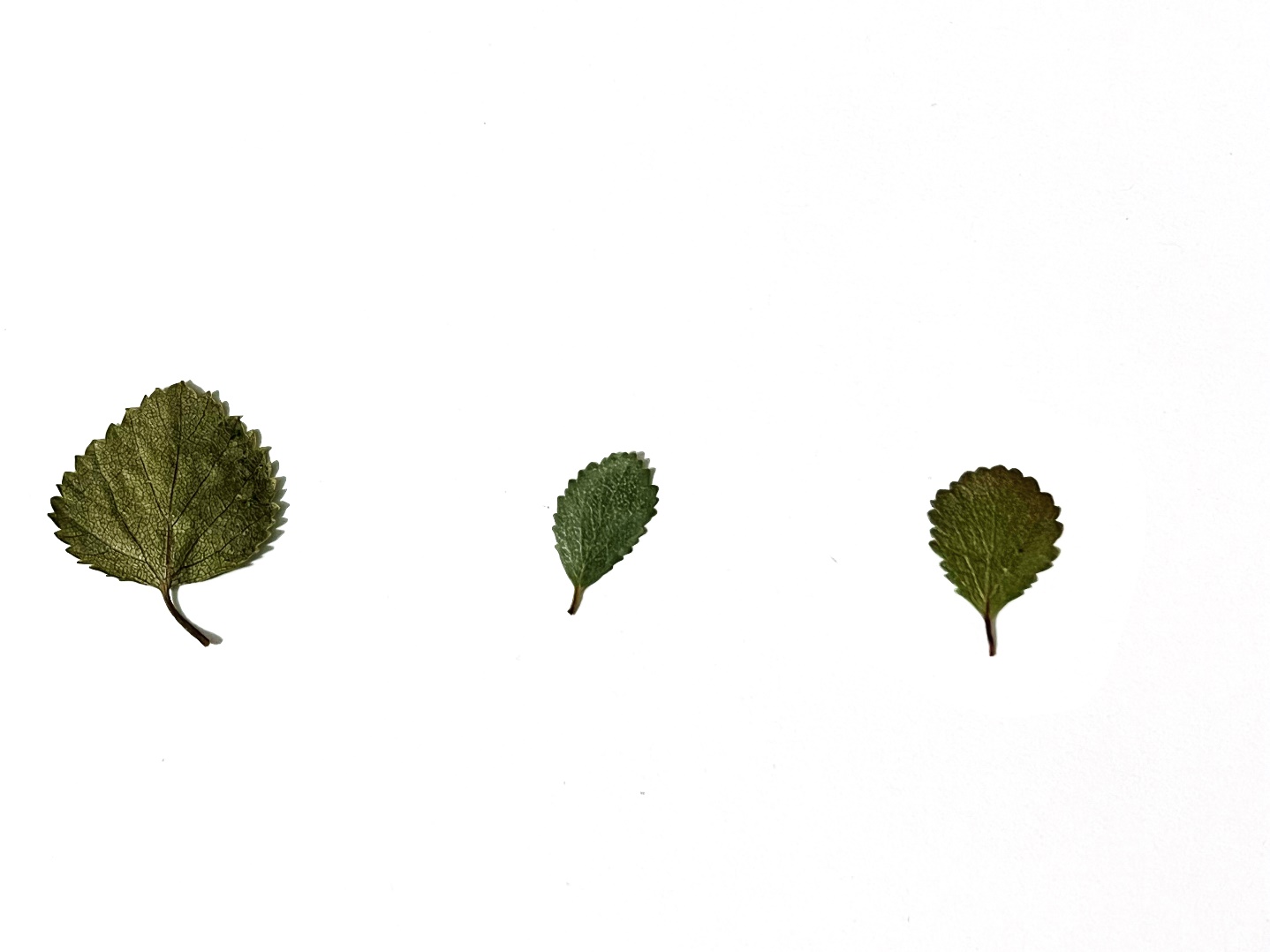


*B. pumila*


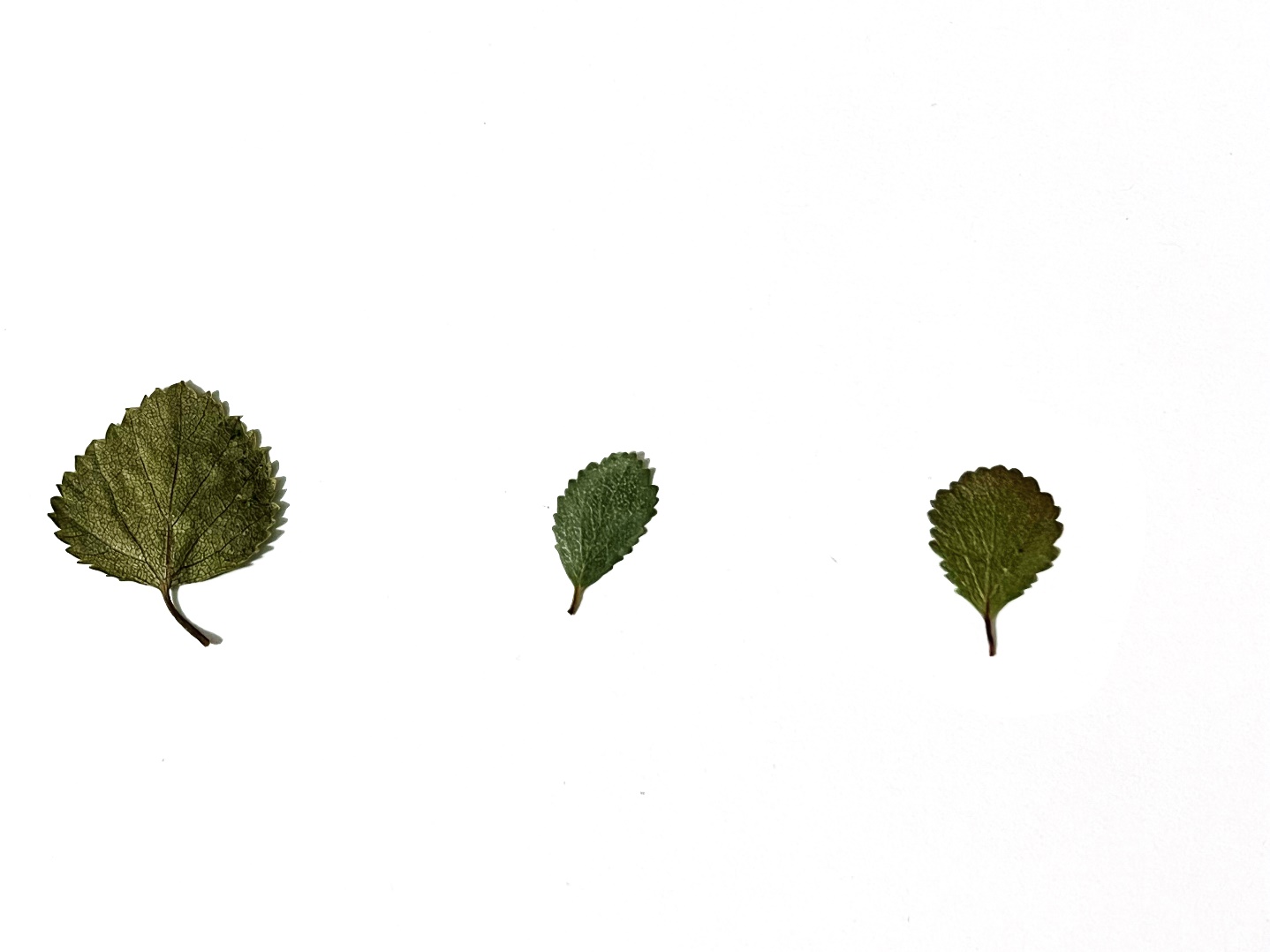


Hybrid *B. occidentalis*

1 cm

###### **Figure S8.** Representative leaf morphology of individuals within the population sample.

######
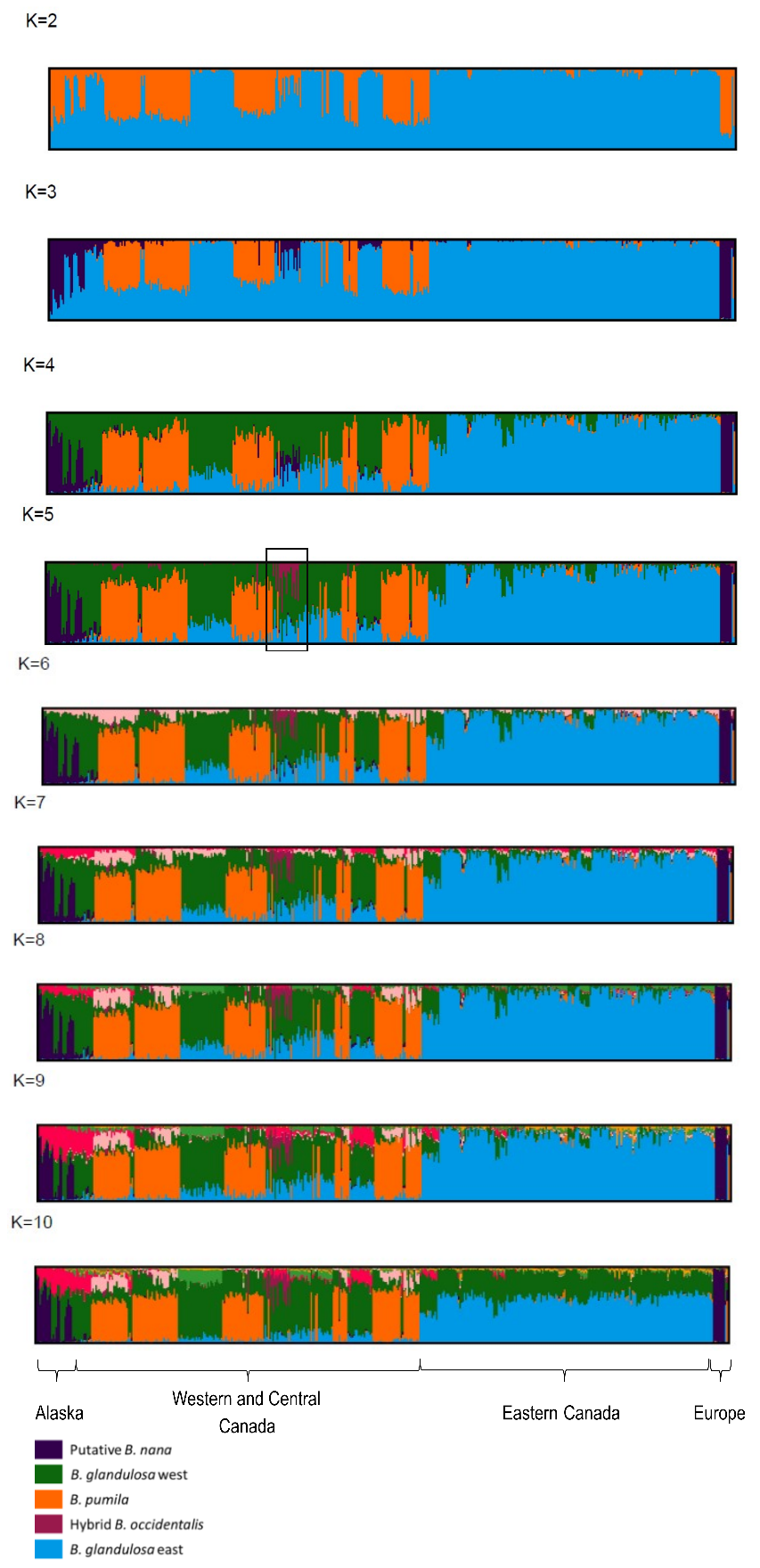
**Figure S9.** Structure barplots for the initial diploid dataset for K = 2 to K = 10. Individuals are represented following a longitudinal gradient: on the far left, individuals from Alaska and on the far right, individuals from Eastern Canada and Europe. The rectangle indicates a population of hybrid individuals detected from K = 5.


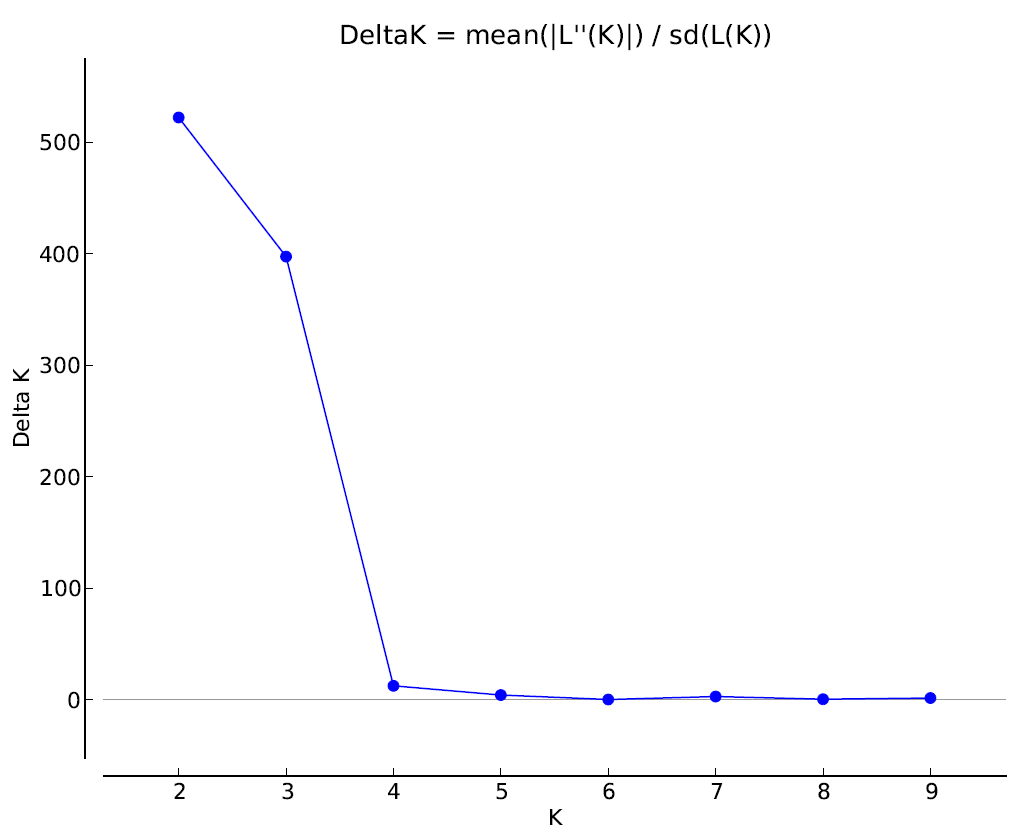


###### **Figure S10.** Delta K vs K (Evanno et al., 2005) for the STRUCTURE analysis using the initial diploid dataset. The plot was constructed using Structure Harvester (Earl and vonHoldt, 2012). Arrow indicates the inferred number of clusters.


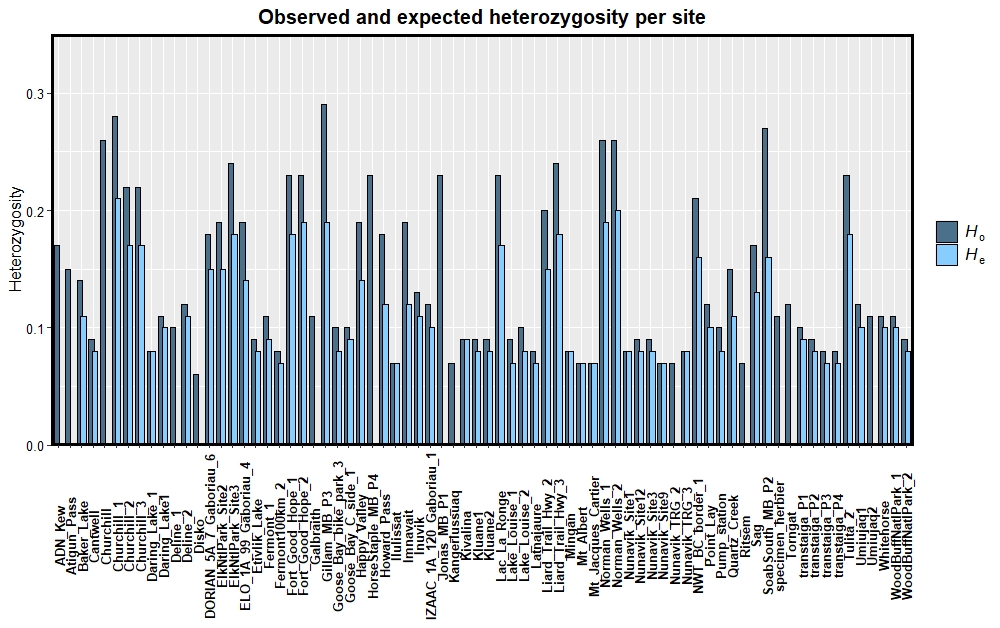


######
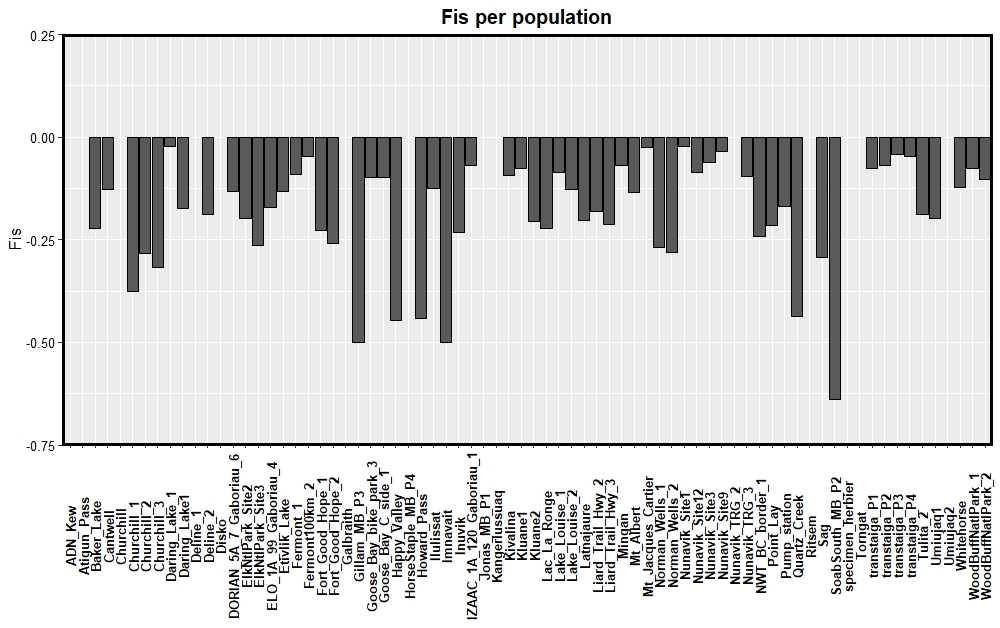
**Figure S11.** Observed (H_O_) and expected (H_E_) heterozygosity of all 74 sampled populations.

###### **Figure S12**. Inbreeding coefficient (F_IS_) of all 74 sampled populations.


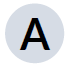

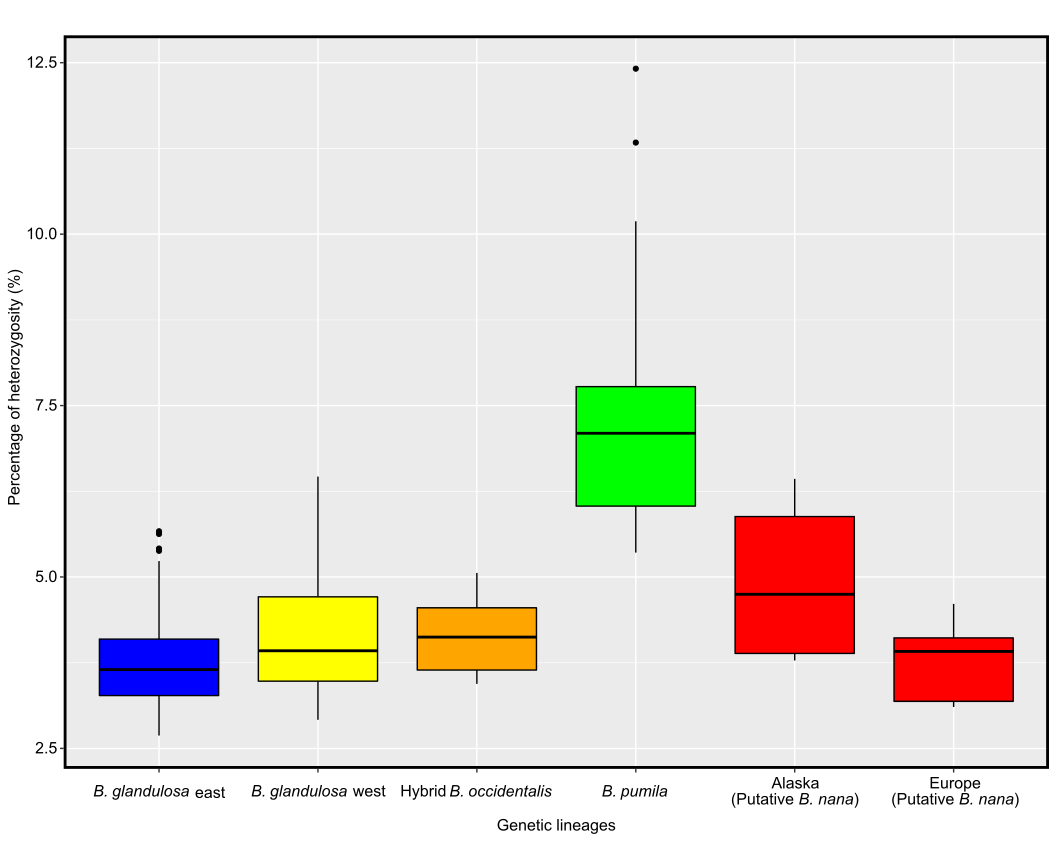

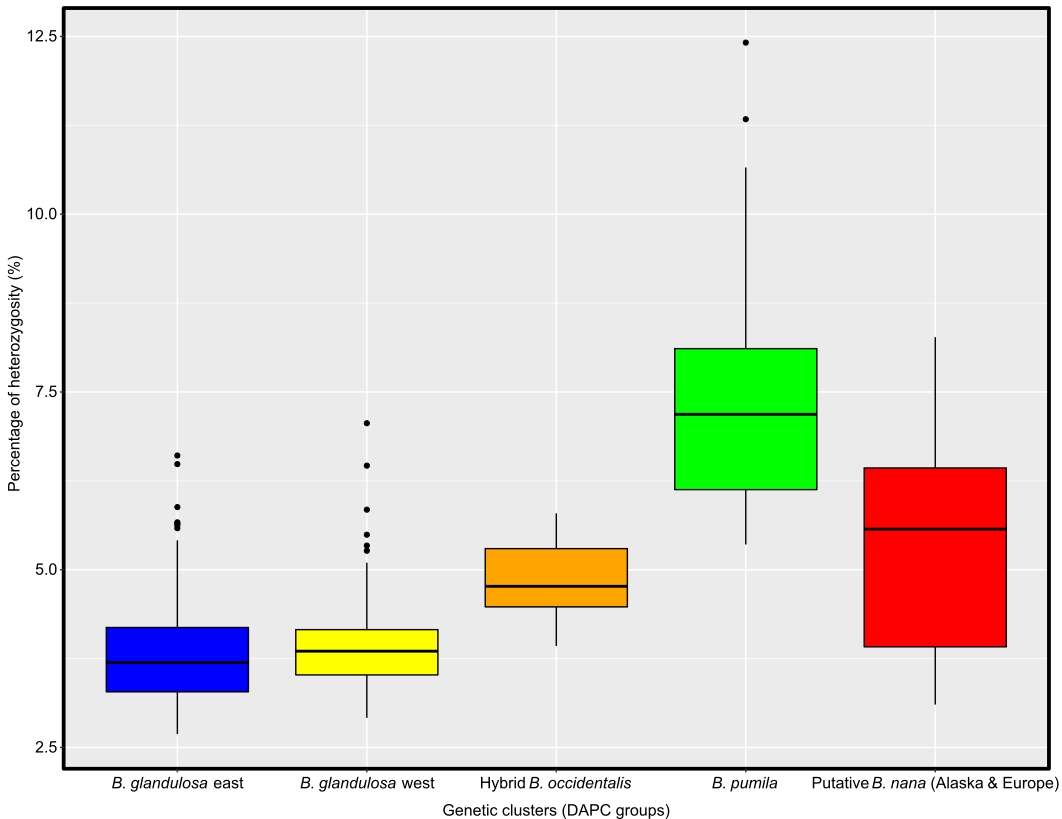

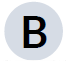


###### **Figure S13.** Percentage of heterozygosity identified with (a) DAPC (genetic clusters), (b) STRUCTURE (genetic lineages).


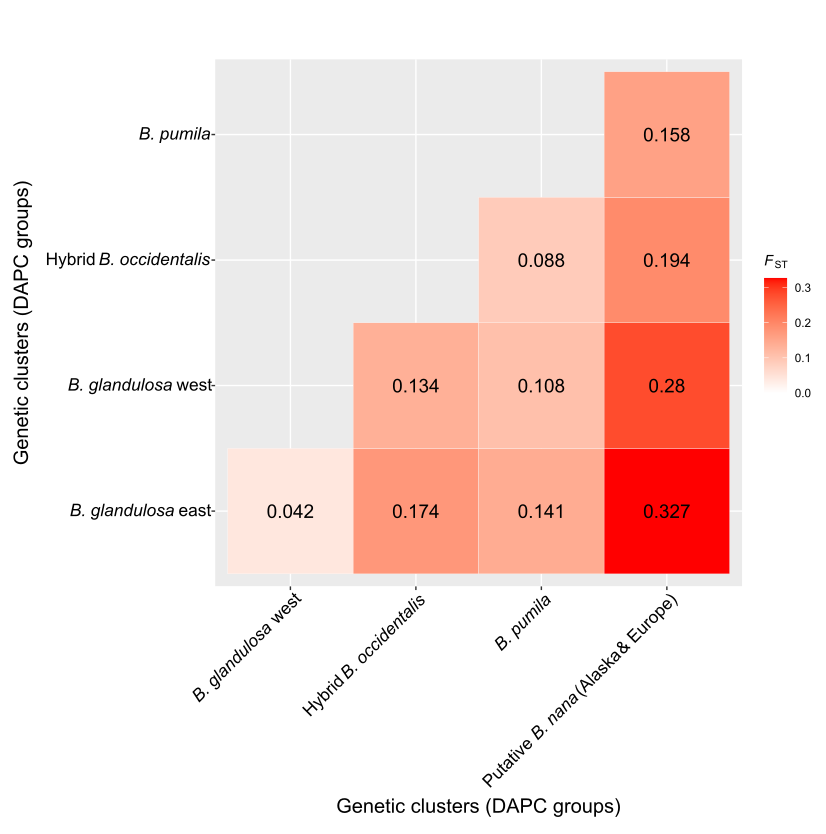


###### **Figure S14.** Pairwise F_ST_ estimated between genetic clusters (DAPC groups).

### Supplementary tables

##### **Table S1.** Selected genomic regions for the Fluidigm "Targeted DNA sequencing" method.

| **Genomic regions** | **Annotation** | **Type** |
| --- | --- | --- |
| rpl14 | rpl14 | chloroplast DNA |
| infA | infA | chloroplast DNA |
| trnK-UUU.matK.b | trnK-UUU.matK.b | chloroplast DNA |
| accD | accD | chloroplast DNA |
| NA.a | NA.a | chloroplast DNA |
| ndhF | ndhF | chloroplast DNA |
| ycf1.e | ycf1.e | chloroplast DNA |
| ycf3 | ycf3 | chloroplast DNA |
| rpl16.rps3 | rpl16_rps3 | chloroplast DNA |
| trnS-GGA.ycf3 | trnS-GGA.ycf3 | chloroplast DNA |
| trnK-UUU.matK.a | trnK-UUU.matK.a | chloroplast DNA |
| petD.rpoA | petD_rpoA | chloroplast DNA |
| ycf1.b | ycf1.b | chloroplast DNA |
| trnT-GGU.trnM-CAU | trnT-GGU.trnM-CAU | chloroplast DNA |
| rps15 | rps15 | chloroplast DNA |
| trnC-GCA | trnC-GCA | chloroplast DNA |
| rps11.rpl36 | rps11_rpl36 | chloroplast DNA |
| ccsA.ndhD | ccsA_ndhD | chloroplast DNA |
| psbE | psbE | chloroplast DNA |
| psbB.psbT | psbB_psbT | chloroplast DNA |
| ycf1.d | ycf1.d | chloroplast DNA |
| atpI.rps2 | atpI_rps2 | chloroplast DNA |
| ycf1.a | ycf1.a | chloroplast DNA |
| NA.b | NA | chloroplast DNA |
| ycf1.c | ycf1.c | chloroplast DNA |
| ndhH | ndhH | chloroplast DNA |
| Bpev01.c1534.g0006 | Chitinase_family_protein | nuclear DNA |
| Bpev01.c0640.g0015 | WRKY_transcription_factor | nuclear DNA |
| Bpev01.c1064.g0008 | ETHYLENE_INSENSITIvE | nuclear DNA |
| Bpev01.c0094.g0029 | Cellulose-synthase-like_C4_LENGTH_673 | nuclear DNA |
| Bpev01.c2498.g0001 | myb_domain_protein_52_LENGTH_249 | nuclear DNA |
| Bpev01.c0022.g0056 | sweep | nuclear DNA |
| Bpev01.c1024.g0009 | WRKY_transcription_factor | nuclear DNA |
| Bpev01.c1333.g0007 | TOPLESS-related_1_LENGTH_1120 | nuclear DNA |
| Bpev01.c0044.g0010 | Riboflavin_biosynthesis_protein_RibD | nuclear DNA |
| Bpev01.c0015.g0045 | WRKY_transcription_factor | nuclear DNA |
| Bpev01.c0480.g0086 | sweep | nuclear DNA |
| Bpev01.c0115.g0060 | Laccase | nuclear DNA |
| Bpev01.c0127.g0014 | KNOTTED1 | nuclear DNA |
| Bpev01.c0727.g0009 | sucrose_synthase_6_LENGTH_942 | nuclear DNA |
| Bpev01.c0455.g0001 | Basic_Chitinase | nuclear DNA |
| Bpev01.c1185.g0007 | Cinnamyl_alcohol_dehydrogenase | nuclear DNA |
| Bpev01.c0024.g0014 | FAR1-related_sequence_6_LENGTH_703 | nuclear DNA |
| Bpev01.c1130.g0008 | sweep | nuclear DNA |
| Bpev01.c0120.g0021 | sweep | nuclear DNA |
| Bpev01.c0004.g0010 | WRKY_transcription_factor | nuclear DNA |
| Bpev01.c0027.g0110 | Riboflavin_biosynthesis_protein_RibD | nuclear DNA |
| Bpev01.c0423.g0009 | Cellulose_synthase | nuclear DNA |
| Bpev01.c0647.g0003 | Myb-related_protein_B | nuclear DNA |
| Bpev01.c0936.g0004 | sweep | nuclear DNA |
| Bpev01.c2361.g0013 | CLAvATA | nuclear DNA |
| Bpev01.c0213.g0022 | myb_domain_protein_1_LENGTH_393 | nuclear DNA |
| Bpev01.c0053.g0026 | time_for_coffee_LENGTH_1555 | nuclear DNA |
| Bpev01.c0163.g0020 | myb_domain_protein_84_LENGTH_310 | nuclear DNA |
| Bpev01.c0442.g0038 | 4-coumarate-CoA_ligase | nuclear DNA |
| Bpev01.c1442.g0003 | Laccase | nuclear DNA |
| Bpev01.c0148.g0013 | KNOTTED1 | nuclear DNA |
| Bpev01.c0067.g0008 | laccase_11_LENGTH_557 | nuclear DNA |
| Bpev01.c2257.g0004 | Chitinase-3-like_protein_1 | nuclear DNA |
| Bpev01.c0281.g0085 | myb_domain_protein_16_LENGTH_326 | nuclear DNA |
| Bpev01.c0051.g0141 | Flavonoid_3-hydroxylase | nuclear DNA |
| Bpev01.c0931.g0020 | Laccase | nuclear DNA |
| Bpev01.c1756.g0002 | myb_domain_protein_101_LENGTH_490 | nuclear DNA |
| Bpev01.c0027.g0040 | KEEP_ON_GOING | nuclear DNA |
| Bpev01.c0357.g0007 | WRKY_transcription_factor | nuclear DNA |
| Bpev01.c0155.g0035 | terpene_synthase_04_LENGTH_877 | nuclear DNA |
| Bpev01.c0115.g0061 | Laccase | nuclear DNA |
| Bpev01.c0800.g0040 | laccase_11_LENGTH_557 | nuclear DNA |
| Bpev01.c0990.g0002 | Phenylalanine_ammonia_lyase | nuclear DNA |
| Bpev01.c0292.g0029 | CONSTANS-LIKE | nuclear DNA |
| Bpev01.c1270.g0001 | cellulose_synthase_like_G2_LENGTH_722 | nuclear DNA |
| Bpev01.c1525.g0004 | Thaumatin_like_protein | nuclear DNA |
| Bpev01.c0817.g0012 | Laccase | nuclear DNA |
| Bpev01.c1484.g0007 | FAR1-related_sequence_9_LENGTH_545 | nuclear DNA |
| Bpev01.c2048.g0002 | FAR1-related_sequence_5_LENGTH_788 | nuclear DNA |
| Bpev01.c1422.g0002 | FAR1-related_sequence_5_LENGTH_788 | nuclear DNA |
| Bpev01.c0245.g0013 | FAR1-related_sequence_7_LENGTH_764 | nuclear DNA |
| Bpev01.c0050.g0040 | WRKY_DNA-binding_protein_21_LENGTH_380 | nuclear DNA |
| Bpev01.c0088.g0111 | Coenzyme_Q-binding_protein_COQ10_homolog_mitochondrial | nuclear DNA |
| Bpev01.c0464.g0025 | wound-responsive_family_protein_LENGTH_684 | nuclear DNA |
| Bpev01.c1786.g0015 | Laccase | nuclear DNA |
| Bpev01.c1786.g0008 | Thaumatin | nuclear DNA |
| Bpev01.c0162.g0074 | Homeobox_protein_knotted-1-like_3 | nuclear DNA |
| Bpev01.c0557.g0025 | MADS-box_transcription_factor_family_protein_LENGTH_248 | nuclear DNA |
| Bpev01.c0000.g0005 | MYB_family_transcription_factor | nuclear DNA |
| Bpev01.c0480.g0059 | FAR1-related_sequence_5_LENGTH_788 | nuclear DNA |
| Bpev01.c1484.g0010 | cellulose_synthase_like_D4_LENGTH_1111 | nuclear DNA |
| Bpev01.c0887.g0005 | Chitinase_family_protein | nuclear DNA |
| Bpev01.c0468.g0003 | Dirigent_protein_17 | nuclear DNA |
| Bpev01.c1544.g0001 | Cornichon_family_protein_LENGTH_137 | nuclear DNA |
| Bpev01.c0762.g0006 | FAR1-related_sequence_5_LENGTH_788 | nuclear DNA |
| Bpev01.c0464.g0028 | 1-aminocyclopropane-1-carboxylate_oxidase | nuclear DNA |
| Bpev01.c0364.g0008 | Cellulose_synthase | nuclear DNA |
| Bpev01.c0762.g0003 | FAR1-related_sequence_5_LENGTH_788 | nuclear DNA |
| Bpev01.c0820.g0010 | sweep | nuclear DNA |
| Bpev01.c0762.g0004 | FAR1-related_sequence_5_LENGTH_788 | nuclear DNA |
| Bpev01.c0135.g0043 | FAR1-related_sequence_5_LENGTH_788 | nuclear DNA |
| Bpev01.c0574.g0037 | vernalization5/VIN3-like_LENGTH_702 | nuclear DNA |
| Bpev01.c1845.g0002 | FAR1-related_sequence_3_LENGTH_851 | nuclear DNA |
| Bpev01.c0036.g0013 | Dirigent_protein_23 | nuclear DNA |
| Bpev01.c0150.g0006 | Laccase | nuclear DNA |
| Bpev01.c0617.g0015 | sweep | nuclear DNA |
| Bpev01.c1147.g0003 | NPR1-LIKE_PROTEIN | nuclear DNA |
| Bpev01.c1200.g0009 | MADS-box_transcription_factor_55 | nuclear DNA |
| Bpev01.c0243.g0039 | CONSTANS-LIKE | nuclear DNA |
| Bpev01.c0374.g0018 | cellulose_synthase_family_protein_LENGTH_985 | nuclear DNA |
| Bpev01.c1439.g0014 | Cycloartenol_synthase | nuclear DNA |
| Bpev01.c0051.g0089 | Phenylalanine_ammonia_lyase | nuclear DNA |
| Bpev01.c0219.g0011.a | Cycloartenol_synthase | nuclear DNA |
| Bpev01.c1520.g0009 | Chitinase_domain-containing_protein_1 | nuclear DNA |
| Bpev01.c1063.g0002 | WRKY_transcription_factor | nuclear DNA |
| Bpev01.c0154.g0083 | Riboflavin_synthase | nuclear DNA |
| Bpev01.c0036.g0059 | AGAMOUS-LIKE | nuclear DNA |
| Bpev01.c0473.g0052 | sweep | nuclear DNA |
| Bpev01.c0129.g0100 | sweep | nuclear DNA |
| Bpev01.c0000.g0174 | MYB_transcription_factor | nuclear DNA |
| Bpev01.c0480.g0087 | cellulose_synthase_6_LENGTH_1084 | nuclear DNA |
| Bpev01.c0978.g0005 | pr-2d | nuclear DNA |
| Bpev01.c1798.g0001 | FAR1-related_sequence_1_LENGTH_774 | nuclear DNA |
| Bpev01.c0127.g0039 | WRKY_transcription_factor | nuclear DNA |
| Bpev01.c0241.g0023 | Riboflavin_kinase | nuclear DNA |
| Bpev01.c1557.g0009 | AGAMOUS-LIKE | nuclear DNA |
| Bpev01.c0051.g0050 | CYTOKININ_OXIDASE | nuclear DNA |
| Bpev01.c0015.g0062 | Cinnamyl_alcohol_dehydrogenase | nuclear DNA |
| Bpev01.c0024.g0013 | FAR1-related_sequence_6_LENGTH_703 | nuclear DNA |
| Bpev01.c0281.g0095 | sweep | nuclear DNA |
| Bpev01.c0415.g0031 | AGAMOUS-LIKE | nuclear DNA |
| Bpev01.c0774.g0007 | sweep | nuclear DNA |
| Bpev01.c0568.g0013 | sucrose_synthase_6_LENGTH_942 | nuclear DNA |
| Bpev01.c0080.g0014 | sweep | nuclear DNA |
| Bpev01.c0240.g0028 | phytochrome_E_LENGTH_1112 | nuclear DNA |
| Bpev01.c0415.g0001 | terpene_synthase_03_LENGTH_565 | nuclear DNA |
| Bpev01.c0919.g0007 | MADS | nuclear DNA |
| Bpev01.c1484.g0008 | FAR1-related_sequence_5_LENGTH_788 | nuclear DNA |
| Bpev01.c0915.g0014 | WRKY_DNA-binding_protein_32_LENGTH_466 | nuclear DNA |
| Bpev01.c0396.g0011 | Riboflavin_biosynthesis_protein_RibBA | nuclear DNA |
| Bpev01.c0000.g0231 | laccase_11_LENGTH_557 | nuclear DNA |
| Bpev01.c0278.g0042 | myb_domain_protein_3r-4_LENGTH_961 | nuclear DNA |
| Bpev01.c0027.g0129 | myb_domain_protein_17_LENGTH_299 | nuclear DNA |
| Bpev01.c0329.g0005 | time_for_coffee_LENGTH_1555 | nuclear DNA |
| Bpev01.c0404.g0002.a | Cinnamoyl_CoA_reductase | nuclear DNA |
| Bpev01.c0281.g0058 | myb_domain_protein_30_LENGTH_323 | nuclear DNA |
| Bpev01.c0022.g0003 | sweep | nuclear DNA |
| Bpev01.c0029.g0102 | sweep | nuclear DNA |
| Bpev01.c0515.g0003 | Cellulose-synthase-like_C5_LENGTH_692 | nuclear DNA |
| Bpev01.c1602.g0015 | myb_domain_protein_65_LENGTH_553 | nuclear DNA |
| Bpev01.c0283.g0010 | terpene_synthase_02_LENGTH_539 | nuclear DNA |
| Bpev01.c1229.g0010 | sweep | nuclear DNA |
| Bpev01.c0106.g0005 | myb_domain_protein_103_LENGTH_320 | nuclear DNA |
| Bpev01.c1150.g0011 | Ferrochelatase | nuclear DNA |
| Bpev01.c0441.g0002 | myb_domain_protein_105_LENGTH_330 | nuclear DNA |
| Bpev01.c0018.g0093 | Cellulose-synthase-like_C5_LENGTH_692 | nuclear DNA |
| Bpev01.c0223.g0007 | Putative_Mg_chelatase | nuclear DNA |
| Bpev01.c1000.g0013 | cellulose_synthase-like_B3_LENGTH_755 | nuclear DNA |
| Bpev01.c0166.g0023 | MADS | nuclear DNA |
| Bpev01.c0713.g0003 | ferrochelatase_2_LENGTH_512 | nuclear DNA |
| Bpev01.c1912.g0004 | terpene_synthase_21_LENGTH_545 | nuclear DNA |
| Bpev01.c0639.g0012 | sweep | nuclear DNA |
| Bpev01.c2141.g0001 | Late_embryogenesis_abundant_protein_group_2_LENGTH_325 | nuclear DNA |
| Bpev01.c0063.g0022 | FAR1-related_sequence_4_LENGTH_730 | nuclear DNA |
| Bpev01.c0441.g0004 | WUSCHEL_RELATED_HOMEOBOX | nuclear DNA |
| Bpev01.c0505.g0019 | WRKY_transcription_factor | nuclear DNA |
| Bpev01.c0162.g0053 | Chitinase-3-like_protein_1 | nuclear DNA |
| Bpev01.c1159.g0008 | vernalization5/VIN3-like_LENGTH_685 | nuclear DNA |
| Bpev01.c0038.g0050 | KNOTTED1 | nuclear DNA |
| Bpev01.c2035.g0009 | Dirigent_protein_11 | nuclear DNA |
| Bpev01.c0042.g0040 | Phenylalanine_ammonia_lyase | nuclear DNA |
| Bpev01.c1211.g0012 | sweep | nuclear DNA |
| Bpev01.c0817.g0011 | MYB_family_transcription_factor | nuclear DNA |
| Bpev01.c0626.g0004 | sweep | nuclear DNA |
| Bpev01.c0817.g0028 | myb_domain_protein_109_LENGTH_399 | nuclear DNA |
| Bpev01.c2080.g0001 | myb_domain_protein_r1_LENGTH_305 | nuclear DNA |
| Bpev01.c1167.g0003 | Late_embryogenesis_abundant_(LEA)_hydroxyproline-rich_glycoprotein_family_LENGTH_243 | nuclear DNA |
| Bpev01.c0746.g0008 | FAR1-related_sequence_5_LENGTH_788 | nuclear DNA |
| Bpev01.c1378.g0027 | Laccase | nuclear DNA |
| Bpev01.c0265.g0013 | myb_domain_protein_93_LENGTH_365 | nuclear DNA |
| Bpev01.c2502.g0003 | Chitinase-like_protein_4 | nuclear DNA |
| Bpev01.c0135.g0066 | WRKY_DNA-binding_protein | nuclear DNA |
| Bpev01.c0684.g0005 | myb_domain_protein_68_LENGTH_374 | nuclear DNA |
| Bpev01.c0171.g0036 | CONSTANS-LIKE | nuclear DNA |
| Bpev01.c0129.g0039 | Cinnamyl_alcohol_dehydrogenase | nuclear DNA |
| Bpev01.c0457.g0007 | Late_embryogenesis_abundant_(LEA)_hydroxyproline-rich_glycoprotein_family_LENGTH_208 | nuclear DNA |
| Bpev01.c0036.g0022 | dehydration-induced_protein_(ERD15)_LENGTH_163 | nuclear DNA |
| Bpev01.c0080.g0082 | 4-coumarate:CoA_ligase_2_LENGTH_556 | nuclear DNA |
| Bpev01.c2048.g0001 | FAR1-related_sequence_5_LENGTH_788 | nuclear DNA |
| Bpev01.c0130.g0004 | Pathogenesis-related_protein_Bet_v_I_family | nuclear DNA |
| Bpev01.c0726.g0006 | FAR1-related_sequence_11_LENGTH_680 | nuclear DNA |
| Bpev01.c0386.g0013 | myb_domain_protein_101_LENGTH_478 | nuclear DNA |
| Bpev01.c1040.g0045 | sweep | nuclear DNA |
| Bpev01.c0299.g0032 | 1-aminocyclopropane-1-carboxylate_oxidase | nuclear DNA |
| Bpev01.c1649.g0007 | WRKY_DNA-binding_protein_2_LENGTH_687 | nuclear DNA |
| Bpev01.c0545.g0014 | FAR1-related_sequence_6_LENGTH_703 | nuclear DNA |
| Bpev01.c0205.g0006 | cellulose_synthase_1_LENGTH_1081 | nuclear DNA |
| Bpev01.c0024.g0012 | FAR1-related_sequence_8_LENGTH_725 | nuclear DNA |
| Bpev01.c0425.g0045 | sweep | nuclear DNA |
| Bpev01.c0344.g0029 | sweep | nuclear DNA |
| Bpev01.c0241.g0007 | myb_domain_protein_74_LENGTH_324 | nuclear DNA |
| Bpev01.c1000.g0001 | chitin_elicitor_receptor_kinase | nuclear DNA |
| Bpev01.c0389.g0047 | myb_domain_protein_3r-4_LENGTH_961 | nuclear DNA |
| Bpev01.c0161.g0100 | AGAMOUS-LIKE | nuclear DNA |
| Bpev01.c1456.g0007 | AGAMOUS-LIKE | nuclear DNA |
| Bpev01.c0523.g0001 | Ethylene-insensitive_ | nuclear DNA |
| Bpev01.c0404.g0002.b | Cinnamoyl_CoA_reductase | nuclear DNA |
| Bpev01.c2137.g0001 | laccase_14_LENGTH_569 | nuclear DNA |
| Bpev01.c0130.g0014 | WUSCHEL_RELATED_HOMEOBOX | nuclear DNA |
| Bpev01.c0169.g0024 | cellulose_synthase-like_A3_LENGTH_556 | nuclear DNA |
| Bpev01.c2220.g0003 | AGAMOUS-LIKE | nuclear DNA |
| Bpev01.c0537.g0004 | myb_domain_protein_3r-4_LENGTH_961 | nuclear DNA |
| Bpev01.c0356.g0013 | Chitinase-like_protein_3 | nuclear DNA |
| Bpev01.c0264.g0039 | sweep | nuclear DNA |
| Bpev01.c0777.g0019 | myb_domain_protein_83_LENGTH_343 | nuclear DNA |
| Bpev01.c1632.g0004 | sweep | nuclear DNA |
| Bpev01.c1106.g0003 | sweep | nuclear DNA |
| Bpev01.c0803.g0018 | 4-coumarate-CoA_ligase | nuclear DNA |
| Bpev01.c0170.g0040 | YODA | nuclear DNA |
| Bpev01.c0088.g0068 | CONSTANS-LIKE | nuclear DNA |
| Bpev01.c1193.g0012 | cellulose_synthase-like_B3_LENGTH_755 | nuclear DNA |
| Bpev01.c1739.g0001 | cellulose_synthase_like_G3_LENGTH_751 | nuclear DNA |
| Bpev01.c1581.g0003 | terpene_synthase_14_LENGTH_569 | nuclear DNA |
| Bpev01.c0425.g0010.b | WRKY_transcription_factor | nuclear DNA |
| Bpev01.c0820.g0004 | FAR1-related_sequence_11_LENGTH_680 | nuclear DNA |
| Bpev01.c1393.g0005 | FAR1-related_sequence_4_LENGTH_730 | nuclear DNA |
| Bpev01.c0603.g0003 | Cellulose_synthase_family_protein_LENGTH_1026 | nuclear DNA |
| Bpev01.c0000.g0232 | laccase_11_LENGTH_557 | nuclear DNA |
| Bpev01.c0718.g0008 | sweep | nuclear DNA |
| Bpev01.c1393.g0003 | FAR1-related_sequence_2_LENGTH_807 | nuclear DNA |
| Bpev01.c0170.g0017 | 1-aminocyclopropane-1-carboxylate_synthase | nuclear DNA |
| Bpev01.c0867.g0003 | MADS-box_transcription_factor_7 | nuclear DNA |
| Bpev01.c0449.g0043 | sweep | nuclear DNA |
| Bpev01.c0402.g0034 | Cellulose_synthase | nuclear DNA |
| Bpev01.c1104.g0030 | myb_domain_protein_108_LENGTH_323 | nuclear DNA |
| Bpev01.c0990.g0003 | ETHYLENE_INSENSITIvE | nuclear DNA |
| Bpev01.c0219.g0011.b | Cycloartenol_synthase | nuclear DNA |
| Bpev01.c0736.g0009 | laccase_1_LENGTH_581 | nuclear DNA |
| Bpev01.c0063.g0021 | FAR1-related_sequence_4_LENGTH_732 | nuclear DNA |
| Bpev01.c0995.g0003 | cellulose_synthase_like_G3_LENGTH_751 | nuclear DNA |
| Bpev01.c1165.g0003 | Phenylalanine_ammonia_lyase | nuclear DNA |
| Bpev01.c2556.g0001 | FAR1-related_sequence_3_LENGTH_706 | nuclear DNA |
| Bpev01.c0772.g0008 | ETHYLENE_INSENSITIvE | nuclear DNA |
| Bpev01.c0126.g0042 | MYB_transcription_factor | nuclear DNA |
| Bpev01.c1673.g0006 | myb_domain_protein_31_LENGTH_330 | nuclear DNA |
| Bpev01.c0023.g0013 | Ferulate_5-hydroxylase | nuclear DNA |
| Bpev01.c0546.g0007 | MADS_BOX_PROTEIN | nuclear DNA |
| Bpev01.c0022.g0148 | WUSCHEL_RELATED_HOMEOBOX | nuclear DNA |
| Bpev01.c2315.g0007 | myb_domain_protein_113_LENGTH_246 | nuclear DNA |
| Bpev01.c0574.g0027 | 4-coumarate-CoA_ligase | nuclear DNA |
| Bpev01.c0357.g0074 | AGAMOUS-LIKE | nuclear DNA |
| Bpev01.c1786.g0017 | Laccase | nuclear DNA |
| Bpev01.c0000.g0006 | Cellulose_synthase | nuclear DNA |
| Bpev01.c0022.g0179 | FAR1-related_sequence_5_LENGTH_788 | nuclear DNA |
| Bpev01.c0134.g0134 | AP2/B3-like_transcriptional_factor_family_protein_LENGTH_341 | nuclear DNA |
| Bpev01.c1235.g0005 | 4-hydroquinone_methyltransferase | nuclear DNA |
| Bpev01.c0777.g0012 | Cellulose_synthase_family_protein_LENGTH_1065 | nuclear DNA |
| Bpev01.c0155.g0008 | KNOTTED1 | nuclear DNA |
| Bpev01.c0762.g0005 | FAR1-related_sequence_5_LENGTH_788 | nuclear DNA |
| Bpev01.c0604.g0005 | CONSTANS-LIKE | nuclear DNA |
| Bpev01.c0066.g0042 | WRKY_transcription_factor | nuclear DNA |
| Bpev01.c0566.g0030 | MADS-box_transcription_factor_family_protein_LENGTH_224 | nuclear DNA |
| Bpev01.c1083.g0003 | KNOX_Arabidopsis_thaliana_meinox_LENGTH_138 | nuclear DNA |
| Bpev01.c0196.g0006 | cellulose_synthase_1_LENGTH_1081 | nuclear DNA |
| Bpev01.c0000.g0018 | sweep | nuclear DNA |
| Bpev01.c0522.g0051 | Caffeic_acid_3-O-methyltransferase | nuclear DNA |
| Bpev01.c0485.g0035.a | KEEP_ON_GOING | nuclear DNA |
| Bpev01.c0425.g0010.a | WRKY_transcription_factor | nuclear DNA |
| Bpev01.c0485.g0035.b | KEEP_ON_GOING | nuclear DNA |
| Bpev01.c0294.g0013 | sucrose_synthase_3_LENGTH_809 | nuclear DNA |
| Bpev01.c0357.g0062 | myb_domain_protein_106_LENGTH_388 | nuclear DNA |
| Bpev01.c0514.g0012 | sweep | nuclear DNA |
| Bpev01.c1800.g0004 | sweep | nuclear DNA |
| Bpev01.c0051.g0185 | sucrose_synthase_4_LENGTH_808 | nuclear DNA |
| Bpev01.c0932.g0014 | WRKY_transcription_factor | nuclear DNA |
| Bpev01.c0333.g0021 | Late_embryogenesis_abundant_(LEA)_hydroxyproline-rich_glycoprotein_family_LENGTH_264 | nuclear DNA |
| Bpev01.c0949.g0008 | cellulose_synthase-like_D3_LENGTH_1145 | nuclear DNA |
| Bpev01.c1259.g0022 | terpene_synthase_04_LENGTH_877 | nuclear DNA |
| Bpev01.c0902.g0015 | cellulose_synthase-like_A3_LENGTH_556 | nuclear DNA |
| Bpev01.c0022.g0024 | WUSCHEL | nuclear DNA |
| Bpev01.c0016.g0055 | cellulose_synthase-like_D1_LENGTH_1036 | nuclear DNA |
| Bpev01.c1520.g0006 | 4-coumarate:CoA_ligase_2_LENGTH_556 | nuclear DNA |
| Bpev01.c1126.g0010 | 4-coumarate-CoA_ligase | nuclear DNA |
| Bpev01.c3352.g0002 | Late_embryogenesis_abundant_(LEA)_hydroxyproline-rich_glycoprotein_family_LENGTH_219 | nuclear DNA |
| Bpev01.c0531.g0003 | FAR1-related_sequence_5_LENGTH_788 | nuclear DNA |
| Bpev01.c0511.g0003 | WRKY_transcription_factor | nuclear DNA |
| Bpev01.c0920.g0001 | expansin_B2_LENGTH_273 | nuclear DNA |
| Bpev01.c0560.g0004 | Cinnamyl_alcohol_dehydrogenase | nuclear DNA |
| Bpev01.c0958.g0004 | ABI3-INTERACTING_PROTEIN | nuclear DNA |
| Bpev01.c0080.g0068 | CONSTANS-LIKE | nuclear DNA |
| Bpev01.c0051.g0051 | Cytokinin_dehydrogenase_3 | nuclear DNA |
| Bpev01.c1188.g0006 | Late_embryogenesis_abundant_(LEA)_protein-related_LENGTH_338 | nuclear DNA |
| Bpev01.c0365.g0033 | expansin_A4_LENGTH_257 | nuclear DNA |
| Bpev01.c0862.g0018 | sweep | nuclear DNA |
| Bpev01.c0148.g0016 | myb_domain_protein_36_LENGTH_333 | nuclear DNA |
| Bpev01.c0058.g0002 | Cellulose-synthase-like_C6_LENGTH_682 | nuclear DNA |
| Bpev01.c0245.g0074 | Cinnamoyl_CoA_reductase | nuclear DNA |
| Bpev01.c0036.g0029 | CYTOKININ_OXIDASE | nuclear DNA |
| Bpev01.c0518.g0052 | Homeobox_protein_knotted-1-like_3 | nuclear DNA |
| Bpev01.c0051.g0139 | Flavonoid_3-hydroxylase | nuclear DNA |
| Bpev01.c0254.g0005 | Dirigent_protein_19 | nuclear DNA |
| Bpev01.c0283.g0015 | MADS-box_transcription_factor_family_protein_LENGTH_224 | nuclear DNA |
| Bpev01.c0719.g0009 | Chitinase_family_protein | nuclear DNA |
| Bpev01.c0717.g0012 | Homeobox_protein_knotted-1-like_7 | nuclear DNA |
| Bpev01.c0383.g0002 | MYB_family_transcription_factor | nuclear DNA |
| Bpev01.c0233.g0001 | Endochitinase | nuclear DNA |
| Bpev01.c0356.g0015 | Chitinase-3-like_protein_2 | nuclear DNA |
| Bpev01.c0356.g0026 | sweep | nuclear DNA |
| Bpev01.c0155.g0034 | terpene_synthase_04_LENGTH_877 | nuclear DNA |
| Bpev01.c1085.g0027 | Pathogenesis-related_protein_Bet_v_I_family | nuclear DNA |
| Bpev01.c1525.g0003 | WUSCHEL_RELATED_HOMEOBOX | nuclear DNA |
| Bpev01.c0135.g0086 | sweep | nuclear DNA |
| Bpev01.c0467.g0012 | WRKY_DNA-binding_protein_4_LENGTH_514 | nuclear DNA |
| Bpev01.c1499.g0002 | myb_domain_protein_3r-3_LENGTH_510 | nuclear DNA |
| Bpev01.c0517.g0009 | AGAMOUS-LIKE | nuclear DNA |
| Bpev01.c1104.g0019 | Craniofacial_development_protein_1 | nuclear DNA |
| Bpev01.c0029.g0157 | CYTOKININ_OXIDASE | nuclear DNA |
| Bpev01.c1089.g0003 | myb_domain_protein_33_LENGTH_520 | nuclear DNA |
| Bpev01.c2000.g0006 | sweep | nuclear DNA |
| Bpev01.c0091.g0046 | FAR1-related_sequence_10_LENGTH_685 | nuclear DNA |
| Bpev01.c0772.g0015 | FAR1-related_sequence_3_LENGTH_851 | nuclear DNA |
| Bpev01.c1167.g0002 | Late_embryogenesis_abundant_(LEA)_hydroxyproline-rich_glycoprotein_family_LENGTH_208 | nuclear DNA |
| Bpev01.c0667.g0006 | myb_domain_protein_103_LENGTH_320 | nuclear DNA |
| Bpev01.c1910.g0004 | laccase_14_LENGTH_569 | nuclear DNA |
| Bpev01.c0147.g0009 | sweep | nuclear DNA |
| Bpev01.c1688.g0009 | sweep | nuclear DNA |
| Bpev01.c0283.g0011 | terpene_synthase_03_LENGTH_565 | nuclear DNA |
| Bpev01.c1930.g0001 | Laccase | nuclear DNA |
| Bpev01.c1040.g0043 | WRKY_transcription_factor | nuclear DNA |
| Bpev01.c0357.g0035 | WRKY_transcription_factor | nuclear DNA |
| Bpev01.c0931.g0003 | WRKY_transcription_factor | nuclear DNA |
| Bpev01.c2315.g0006 | myb_domain_protein_113_LENGTH_246 | nuclear DNA |
| Bpev01.c0349.g0009 | sweep | nuclear DNA |
| Bpe.Chr1.3434554.3435722 | NA | nuclear DNA |
| Bpe.Chr9.25934779.25935899 | NA | nuclear DNA |
| Bpe.Chr4.1471651.1472943 | NA | nuclear DNA |
| Bpe.Chr13.9843158.9844309 | NA | nuclear DNA |
| Bpe.Chr1.40955829.40957225 | NA | nuclear DNA |
| Bpe.Chr13.1587827.1589271 | NA | nuclear DNA |
| Bpe.Chr11.31963431.31964633 | NA | nuclear DNA |
| Bpe.Chr13.6989261.6990640 | NA | nuclear DNA |
| Bpe.Chr14.12174532.12175903 | NA | nuclear DNA |
| Bpe.Chr7.4303948.4305249 | NA | nuclear DNA |
| Bpe.Chr13.5142019.5143457 | NA | nuclear DNA |
| Bpe.Chr8.3539137.3540239 | NA | nuclear DNA |
| Bpe.Chr1.4288028.4289242 | NA | nuclear DNA |
| Bpe.Chr9.1528045.1529437 | NA | nuclear DNA |
| Bpe.Chr2.22984392.22985553 | NA | nuclear DNA |
| Bpe.Chr8.19859415.19860579 | NA | nuclear DNA |
| Bpe.Chr12.23288752.23290001 | NA | nuclear DNA |
| Bpe.Chr4.6155454.6156597 | NA | nuclear DNA |
| Bpe.Chr2.32760937.32762170 | NA | nuclear DNA |
| Bpe.Chr14.3931403.3932516 | NA | nuclear DNA |
| Bpe.Chr7.6039931.6041299 | NA | nuclear DNA |
| Bpe.Chr12.1137276.1138397 | NA | nuclear DNA |
| Bpe.Chr5.3173922.3175044 | NA | nuclear DNA |
| Bpe.Chr12.25617005.25618106 | NA | nuclear DNA |
| Bpe.Chr4.4661221.4662464 | NA | nuclear DNA |
| Bpe.Chr5.20518519.20519713 | NA | nuclear DNA |
| Bpe.Chr12.19619860.19621189 | NA | nuclear DNA |
| Bpe.Chr8.4318630.4319744 | NA | nuclear DNA |
| Bpe.Chr8.22932293.22933783 | NA | nuclear DNA |
| Bpe.Chr5.26112020.26113303 | NA | nuclear DNA |
| Bpe.Chr11.11495185.11496637 | NA | nuclear DNA |
| Bpe.Chr4.7405605.7406825 | NA | nuclear DNA |
| Bpe.Chr2.1527728.1529186 | NA | nuclear DNA |
| Bpe.Chr12.21516154.21517255 | NA | nuclear DNA |
| Bpe.Chr7.30307205.30308310 | NA | nuclear DNA |
| Bpe.Chr1.31921635.31923004 | NA | nuclear DNA |
| Bpe.Chr2.2918943.2920116 | NA | nuclear DNA |
| Bpe.Chr12.5449257.5450476 | NA | nuclear DNA |
| Bpe.Chr1.36087104.36088373 | NA | nuclear DNA |
| Bpe.Chr4.3934287.3935717 | NA | nuclear DNA |
| Bpe.Chr1.11119457.11120658 | NA | nuclear DNA |
| Bpe.Chr4.22046544.22047651 | NA | nuclear DNA |
| Bpe.Chr9.21838846.21839996 | NA | nuclear DNA |
| Bpe.Chr1.44140915.44142272 | NA | nuclear DNA |
| Bpe.Chr3.18446759.18448052 | NA | nuclear DNA |
| Bpe.Chr10.10691698.10692975 | NA | nuclear DNA |
| Bpe.Chr4.23492145.23493354 | NA | nuclear DNA |
| Bpe.Chr7.20307570.20308997 | NA | nuclear DNA |
| Bpe.Chr9.25034067.25035561 | NA | nuclear DNA |
| Bpe.Chr5.24341328.24342513 | NA | nuclear DNA |
| Bpe.Chr2.4978161.4979295 | NA | nuclear DNA |
| Bpe.Chr1.33439514.33440966 | NA | nuclear DNA |
| Bpe.Chr3.246252.247423 | NA | nuclear DNA |
| Bpe.Chr1.37382422.37383559 | NA | nuclear DNA |
| Bpe.Chr14.13650693.13651918 | NA | nuclear DNA |
| Bpe.Chr5.20255231.20256591 | NA | nuclear DNA |
| Bpe.Chr5.24642204.24643314 | NA | nuclear DNA |
| Bpe.Chr12.5427752.5429185 | NA | nuclear DNA |
| Bpe.Chr5.25621555.25622678 | NA | nuclear DNA |
| Bpe.Chr4.15597195.15598528 | NA | nuclear DNA |
| Bpe.Chr2.28180161.28181325 | NA | nuclear DNA |
| Bpe.Chr1.2795368.2796627 | NA | nuclear DNA |
| Bpe.Chr4.19181578.19182987 | NA | nuclear DNA |
| Bpe.Chr9.22271512.22272846 | NA | nuclear DNA |
| Bpe.Chr11.29467695.29469112 | NA | nuclear DNA |
| Bpe.Chr2.15074743.15075898 | NA | nuclear DNA |
| Bpe.Chr11.24094254.24095698 | NA | nuclear DNA |
| Bpe.Chr12.22775418.22776688 | NA | nuclear DNA |
| Bpe.Chr12.3996816.3998050 | NA | nuclear DNA |
| Bpe.Chr9.25442436.25443658 | NA | nuclear DNA |
| Bpe.Chr2.5123548.5124795 | NA | nuclear DNA |
| Bpe.Chr12.18211288.18212414 | NA | nuclear DNA |
| Bpe.Chr9.13421062.13422423 | NA | nuclear DNA |
| Bpe.Chr9.4360646.4361791 | NA | nuclear DNA |
| Bpe.Chr1.7226322.7227440 | NA | nuclear DNA |
| Bpe.Chr9.2477031.2478159 | NA | nuclear DNA |
| Bpe.Chr7.21582588.21583768 | NA | nuclear DNA |
| Bpe.Chr7.29742747.29743927 | NA | nuclear DNA |
| Bpe.Chr8.22944122.22945241 | NA | nuclear DNA |
| Bpe.Chr10.9094399.9095667 | NA | nuclear DNA |
| Bpe.Chr14.4111394.4112531 | NA | nuclear DNA |
| Bpe.Chr4.14221405.14222516 | NA | nuclear DNA |
| Bpe.Chr13.1657330.1658717 | NA | nuclear DNA |
| Bpe.Chr3.3483736.3484856 | NA | nuclear DNA |
| Bpe.Chr5.684053.685190 | NA | nuclear DNA |
| Bpe.Chr14.12114964.12116206 | NA | nuclear DNA |
| Bpe.Chr1.44522730.44523979 | NA | nuclear DNA |
| Bpe.Chr6.32939613.32940834 | NA | nuclear DNA |
| Bpe.Chr7.5565337.5566556 | NA | nuclear DNA |
| Bpe.Chr5.1220750.1221863 | NA | nuclear DNA |
| Bpe.Chr3.3789312.3790569 | NA | nuclear DNA |
| Bpe.Chr7.6744132.6745284 | NA | nuclear DNA |
| Bpe.Chr1.7656734.7657938 | NA | nuclear DNA |
| Bpe.Chr5.4439871.4440999 | NA | nuclear DNA |
| Bpe.Chr5.4673331.4674529 | NA | nuclear DNA |
| Bpe.Chr8.17186354.17187836 | NA | nuclear DNA |
| Bpe.Chr13.16733048.16734522 | NA | nuclear DNA |
| Bpe.Chr10.16075117.16076219 | NA | nuclear DNA |
| Bpe.Chr9.3893488.3894886 | NA | nuclear DNA |
| Bpe.Chr14.4115385.4116666 | NA | nuclear DNA |
| Bpe.Chr8.8571697.8573060 | NA | nuclear DNA |
| Bpe.Chr14.5473819.5474967 | NA | nuclear DNA |
| Bpe.Chr6.23809922.23811068 | NA | nuclear DNA |
| Bpe.Chr8.11585.12692 | NA | nuclear DNA |
| Bpe.Chr11.26324250.26325352 | NA | nuclear DNA |
| Bpe.Chr5.1729184.1730504 | NA | nuclear DNA |
| Bpe.Chr9.3062809.3063948 | NA | nuclear DNA |
| Bpe.Chr4.18689707.18690858 | NA | nuclear DNA |
| Bpe.Chr11.23937857.23939336 | NA | nuclear DNA |
| Bpe.Chr3.22227893.22229264 | NA | nuclear DNA |
| Bpe.Chr1.10079457.10080834 | NA | nuclear DNA |
| Bpe.Chr3.20658220.20659405 | NA | nuclear DNA |
| Bpe.Chr6.26566646.26568041 | NA | nuclear DNA |
| Bpe.Chr2.23425996.23427286 | NA | nuclear DNA |
| Bpe.Chr1.7410989.7412172 | NA | nuclear DNA |
| Bpe.Chr1.36294925.36296215 | NA | nuclear DNA |
| Bpe.Chr12.775262.776700 | NA | nuclear DNA |
| Bpe.Chr4.11360593.11361745 | NA | nuclear DNA |
| Bpe.Chr9.1476739.1478047 | NA | nuclear DNA |
| Bpe.Chr13.10873235.10874551 | NA | nuclear DNA |
| Bpe.Chr10.5580372.5581605 | NA | nuclear DNA |
| Bpe.Chr10.17310952.17312342 | NA | nuclear DNA |
| Bpe.Chr9.2830376.2831493 | NA | nuclear DNA |
| Bpe.Chr1.30650090.30651569 | NA | nuclear DNA |
| Bpe.Chr4.11604649.11605855 | NA | nuclear DNA |
| Bpe.Chr9.1364816.1365972 | NA | nuclear DNA |
| Bpe.Chr13.15513539.15514672 | NA | nuclear DNA |
| Bpe.Chr13.15020319.15021514 | NA | nuclear DNA |
| Bpe.Chr6.31131274.31132616 | NA | nuclear DNA |
| Bpe.Chr1.32342765.32344216 | NA | nuclear DNA |
| Bpe.Chr2.16668558.16669696 | NA | nuclear DNA |
| Bpe.Chr7.18790599.18791803 | NA | nuclear DNA |
| Bpe.Chr5.22298563.22299798 | NA | nuclear DNA |
| Bpe.Chr8.4188775.4189942 | NA | nuclear DNA |
| Bpe.Chr5.23427664.23428944 | NA | nuclear DNA |
| Bpe.Chr14.13570871.13572185 | NA | nuclear DNA |
| Bpe.Chr1.9600737.9601987 | NA | nuclear DNA |
| Bpe.Chr6.7646155.7647303 | NA | nuclear DNA |
| Bpe.Chr5.5050499.5051646 | NA | nuclear DNA |
| Bpe.Chr12.20502214.20503367 | NA | nuclear DNA |
| Bpe.Chr12.12861118.12862375 | NA | nuclear DNA |
| Bpe.Chr5.17325374.17326645 | NA | nuclear DNA |
| Bpe.Chr4.21291136.21292258 | NA | nuclear DNA |
| ITS2 | NA | nuclear DNA |
| Bpe.Chr13.18173385.18174486 | NA | nuclear DNA |
| Bpe.Chr3.17183812.17185205 | NA | nuclear DNA |
| Bpe.Chr1.39167754.39168867 | NA | nuclear DNA |
| Bpe.Chr11.29185337.29186511 | NA | nuclear DNA |
| Bpe.Chr12.22883612.22884718 | NA | nuclear DNA |
| Bpe.Chr3.11140773.11142181 | NA | nuclear DNA |

##### **Table S2.** Populations of individuals retained in the initial diploid dataset.

| **Population** | **Province / State^a^** | **Latitude** | **Longitude** | **Plant material** | **Number of individuals** |
| --- | --- | --- | --- | --- | --- |
| Kivalina | AK | 67.80911 | -164.33003 | leaf powder | 2 |
| Quartz_Creek | AK | 65.34428 | -164.31474 | leaf powder | 2 |
| Point_Lay | AK | 69.86405 | -162.0901 | leaf powder | 3 |
| Howard_Pass | AK | 68.24509 | -156.79773 | leaf powder | 3 |
| Etivlik_Lake | AK | 68.11991 | -156.082949 | leaf powder | 2 |
| Galbraith | AK | 68.45230667 | -149.4867233 | leaf powder | 1 |
| Atigun_Pass | AK | 68.17179333 | -149.4418233 | leaf powder | 1 |
| Imnavait | AK | 68.62545667 | -149.3239567 | leaf powder | 2 |
| Pump_station | AK | 68.40272667 | -149.3228533 | leaf powder | 3 |
| Sag | AK | 68.76465 | -148.89743 | leaf powder | 3 |
| Happy_Valley | AK | 69.14795333 | -148.8442933 | leaf powder | 2 |
| Cantwell | AK | 63.23815 | -148.794067 | leaf powder | 3 |
| Kluane1 | YT | 60.97617222 | -138.4106722 | leaf powder | 3 |
| Kluane2 | YT | 60.9974 | -138.2634778 | leaf powder | 2 |
| Inuvik | NT | 67.205972 | -135.643241 | leaf powder | 2 |
| Whitehorse | YT | 60.561074 | -135.1402777 | leaf powder | 3 |
| Fort_Good_Hope_2 | NT | 66.24055556 | -128.6275 | buds, leaves, cambium | 3 |
| Fort_Good_Hope_1 | NT | 66.28694444 | -128.6188889 | buds, leaves, cambium | 4 |
| Norman_Wells_2 | NT | 65.28944444 | -126.8477778 | buds, leaves, cambium | 4 |
| Norman_Wells_1 | NT | 65.28 | -126.7552778 | buds, leaves, cambium | 4 |
| Tulita_2 | NT | 64.86987778 | -125.5183861 | buds, leaves, cambium | 10 |
| Deline_2 | NT | 65.2031 | -123.4396861 | buds, leaves, cambium | 2 |
| Deline_1 | NT | 65.18524167 | -123.4065222 | buds, leaves, cambium | 1 |
| Liard_Trail_Hwy_2 | NT | 60.44325 | -123.35957 | buds, leaves, cambium | 13 |
| Liard_Trail_Hwy_3 | NT | 60.93013 | -123.1063 | buds, leaves, cambium | 12 |
| NWT_BC_border_1 | NT | 60.00447 | -122.93957 | buds, leaves, cambium | 6 |
| Lake_Louise_1 | AB | 51.718331 | -116.4917516 | buds | 17 |
| Lake_Louise_2 | BC | 51.412691 | -116.344841 | buds | 13 |
| ELO_1A_99_Gaboriau_4 | NT | 62.7855 | -115.35924 | buds, leaves, cambium | 15 |
| DORIAN_5A_7_Gaboriau_6 | NT | 63.80732 | -114.97659 | buds, leaves, cambium | 15 |
| IZAAC_1A_120_Gaboriau_1 | NT | 64.09857 | -114.17657 | buds, leaves, cambium | 16 |
| WoodBuffNatlPark_2 | NT | 60.123378 | -113.464494 | buds | 14 |
| WoodBuffNatlPark_1 | NT | 60.02388 | -112.94384 | buds | 15 |
| ElkNtlPark_Site2 | AB | 53.64408 | -112.86197 | buds | 5 |
| ElkNtlPark_Site3 | AB | 53.6566 | -112.80289 | buds | 5 |
| Daring_Lake1 | NT | 64.871 | -111.5510556 | leaf powder | 2 |
| Daring_Lake_1 | NT | 65.86666667 | -111.5333333 | buds, leaves, cambium | 15 |
| Lac_La_Ronge | SK | 54.95315 | -105.3545167 | buds | 14 |
| SoabSouth_MB_P2 | MB | 55.21230556 | -98.43012222 | buds, leaves, cambium | 3 |
| HorseStaple_MB_P4 | MB | 55.76578889 | -97.83752222 | buds, leaves, cambium | 1 |
| Jonas_MB_P1 | MB | 55.66028333 | -97.78945 | buds, leaves, cambium | 1 |
| Baker_Lake | NU | 64.33769 | -95.91664667 | leaf powder | 2 |
| Gillam_MB_P3 | MB | 56.41714722 | -94.36820833 | buds, leaves, cambium | 3 |
| Churchill_3 | MB | 58.76016333 | -94.01399333 | leaves, leaf powder | 2 |
| Churchill_1 | MB | 58.75519 | -93.84704333 | leaves | 2 |
| Churchill_2 | MB | 58.67504667 | -93.84060667 | leaves, leaf powder | 3 |
| Churchill | MB | 58.73366 | -93.8075 | leaf powder | 1 |
| Nunavik_Site3 | QC | 61.333482 | -77.469065 | leaves | 12 |
| transtaiga_P4 | QC | 53.34786389 | -77.18883333 | buds, leaves, cambium | 14 |
| Umiujaq1 | QC | 56.55735333 | -76.55125278 | leaf powder | 2 |
| Umiujaq2 | QC | 56.56037831 | -76.47943889 | leaf powder | 1 |
| transtaiga_P3 | QC | 53.47278056 | -75.36796667 | buds, leaves, cambium | 15 |
| Nunavik_Site1 | QC | 59.70998 | -74.94111 | leaves | 14 |
| Nunavik_Site9 | QC | 56.33333 | -74.45 | leaves | 11 |
| transtaiga_P2 | QC | 53.62531944 | -74.10755278 | buds, leaves, cambium | 15 |
| transtaiga_P1 | QC | 53.92425556 | -72.45769722 | buds, leaves, cambium | 15 |
| Nunavik_Site12 | QC | 55.411666 | -69.57743 | leaves | 15 |
| Fermont100km_2 | QC | 52.251872 | -67.69945 | buds, leaves, cambium | 16 |
| Fermont_1 | QC | 52.774452 | -67.181315 | buds, leaves, cambium | 16 |
| Mt_Albert | QC | 48.93429722 | -66.17456944 | leaf powder | 2 |
| Mt_Jacques_Cartier | QC | 49.00030833 | -65.94340556 | leaf powder | 3 |
| Mingan | QC | 50.31279 | -63.98697 | buds | 13 |
| Nunavik_TRG_3 | NL | 57.88543 | -63.5219 | leaves | 3 |
| Nunavik_TRG_2 | NL | 57.66328 | -63.02761 | leaves | 1 |
| Torngat | QC | 58.45 | -62.79921333 | leaf powder | 1 |
| Goose_Bay_C_side_1 | NL | 53.334899 | -60.428878 | buds, leaves, cambium | 15 |
| Goose_Bay_bike_park_3 | NL | 53.305936 | -60.345706 | buds, leaves, cambium | 15 |
| Disko | Greenland | 69.26583333 | -53.46563889 | leaf powder | 1 |
| Ilulissat | Greenland | 69.24539815 | -51.11336111 | leaf powder | 2 |
| Kangerlussuaq | Greenland | 67.02813889 | -50.66458333 | leaf powder | 1 |
| Ritsem | Sweden | 67.82368519 | 17.71385185 | leaf powder | 1 |
| Latnajaure | Sweden | 68.35662037 | 18.49308333 | leaf powder | 3 |
| specimen_herbier | QC | NA | NA | leaves herbarium | 1 |
| ADN_Kew | NA | NA | NA | ADN Kew | 1 |

a: AB = Alberta, AK = Alaska, BC = British Columbia, MB = Manitoba, NA = missing information, NL = Newfoundland and Labrador, NT = Northwest Territories, NU = Nunavut, QC = Quebec, SK = Saskatchewan, YT = Yukon.

##### **Table S3.** Assignment of ploidy level, genetic cluster (DAPC group), genetic lineage and chlorotype memberships to each individual within the initial diploid dataset.

| **Individuals** | **Ploidy^a^** | **DAPC groups** | **Lineage** | **Haplotype^b^** |
| --- | --- | --- | --- | --- |
| BG81 | D | Putative B. nana | Alaska | C |
| BG82 | U | Putative B. nana | Alaska | C |
| BG83 | U | Putative B. nana | Alaska | C |
| BG85 | U | Putative B. nana | excluded | C |
| BG77 | D | Putative B. nana | Alaska | A |
| BG78 | U | Putative B. nana | Alaska | C |
| BG79 | D | Putative B. nana | Alaska | C |
| BG86 | U | Putative B. nana | Alaska | C |
| BG87 | U | Putative B. nana | excluded | NA |
| BG88 | U | Putative B. nana | Alaska | NA |
| BG89 | D | B. glandulosa west | B. glandulosa west | A |
| BG90 | PD | B. glandulosa west | B. glandulosa west | A |
| BG97 | PD | B. glandulosa west | B. glandulosa west | C |
| BG93 | TR | B. glandulosa west | B. glandulosa west | C |
| BG101 | U | Putative B. nana | excluded | C |
| BG103 | U | Putative B. nana | excluded | C |
| BG104 | D | B. glandulosa west | B. glandulosa west | C |
| BG105 | PD | B. glandulosa west | B. glandulosa west | A |
| BG106 | D | B. glandulosa west | B. glandulosa west | C |
| BG107 | U | Putative B. nana | excluded | NA |
| BG108 | TR | Putative B. nana | excluded | C |
| BG109 | U | Putative B. nana | excluded | C |
| BG99 | U | Putative B. nana | excluded | A |
| BG100 | U | Putative B. nana | excluded | A |
| BG74 | PD | B. glandulosa west | B. glandulosa west | A |
| BG75 | PD | B. glandulosa west | B. glandulosa west | A |
| BG76 | PD | B. glandulosa west | B. glandulosa west | A |
| BG146 | D | B. glandulosa west | B. glandulosa west | A |
| BG147 | D | B. glandulosa west | B. glandulosa west | A |
| BG148 | PD | B. glandulosa west | excluded | A |
| BG150 | D | B. glandulosa west | excluded | A |
| BG151 | PD | B. glandulosa west | B. glandulosa west | A |
| BG50 | D | B. glandulosa west | B. glandulosa west | NA |
| BG51 | T | B. glandulosa west | B. glandulosa west | A |
| BG19 | D | B. glandulosa west | B. glandulosa west | A |
| BG20 | PD | B. glandulosa west | B. glandulosa west | A |
| BG21 | D | B. glandulosa west | B. glandulosa west | A |
| FortGoodHope_1 | T | B. pumila | excluded | A |
| FortGoodHope_2 | PT | B. pumila | B. pumila | M |
| FortGoodHope_3 | PT | B. pumila | excluded | A |
| FortGoodHope_11 | PT | B. pumila | excluded | A |
| FortGoodHope_12 | PT | B. pumila | excluded | L |
| FortGoodHope_14 | PT | B. pumila | excluded | M |
| FortGoodHope_15 | T | B. pumila | excluded | M |
| Normanwells_P3_6 | PT | B. pumila | B. pumila | A |
| Normanwells_P3_7 | PT | B. pumila | excluded | NA |
| Normanwells_P3_8 | PT | B. pumila | B. pumila | A |
| Normanwells_P3_10 | PT | B. pumila | excluded | A |
| Normanwells_P1_6 | P | B. pumila | B. pumila | A |
| Normanwells_P1_7 | PT | B. pumila | B. pumila | A |
| Normanwells_P1_8 | PT | B. pumila | B. pumila | A |
| Normanwells_P1_10 | PT | B. pumila | B. pumila | C |
| Tulita_P2_1 | PT | B. pumila | B. pumila | A |
| Tulita_P2_2 | T | B. pumila | B. pumila | A |
| Tulita_P2_3 | PT | B. pumila | B. pumila | A |
| Tulita_P2_4 | PT | B. pumila | B. pumila | A |
| Tulita_P2_5 | P | B. pumila | B. pumila | A |
| Tulita_P2_6 | PT | B. pumila | B. pumila | A |
| Tulita_P2_7 | PT | B. pumila | B. pumila | A |
| Tulita_P2_8 | PT | B. pumila | excluded | A |
| Tulita_P2_9 | PT | B. pumila | excluded | A |
| Tulita_P2_10 | T | B. pumila | excluded | NA |
| Decline_12 | D | B. glandulosa west | excluded | A |
| Decline_14 | PD | B. glandulosa west | B. glandulosa west | A |
| Decline_1 | D | B. glandulosa west | B. glandulosa west | D |
| LiardTrails_Hwy2_P2_1 | T | B. pumila | B. pumila | A |
| LiardTrails_Hwy2_P2_2 | T | B. pumila | B. pumila | A |
| LiardTrails_Hwy2_P2_3 | T | B. pumila | B. pumila | A |
| LiardTrails_Hwy2_P2_4 | T | B. pumila | B. pumila | M |
| LiardTrails_Hwy2_P2_6 | T | B. pumila | B. pumila | A |
| LiardTrails_Hwy2_P2_7 | T | B. pumila | B. pumila | A |
| LiardTrails_Hwy2_P2_8 | T | B. pumila | B. pumila | A |
| LiardTrails_Hwy2_P2_9 | T | B. pumila | B. pumila | R |
| LiardTrails_Hwy2_P2_10 | T | B. pumila | B. pumila | A |
| LiardTrails_Hwy2_P2_11 | T | B. pumila | B. pumila | A |
| LiardTrails_Hwy2_P2_12 | T | B. pumila | B. pumila | A |
| LiardTrails_Hwy2_P2_14 | T | B. pumila | B. pumila | A |
| LiardTrails_Hwy2_P2_15 | T | B. pumila | B. pumila | A |
| Liardtrail_P3_1 | T | B. pumila | B. pumila | C |
| Liardtrail_P3_2 | T | B. pumila | B. pumila | A |
| Liardtrail_P3_3 | T | B. pumila | B. pumila | A |
| Liardtrail_P3_4 | PT | B. pumila | B. pumila | A |
| Liardtrail_P3_6 | T | B. pumila | B. pumila | A |
| Liardtrail_P3_7 | T | B. pumila | B. pumila | A |
| Liardtrail_P3_8 | T | B. pumila | B. pumila | A |
| Liardtrail_P3_9 | PT | B. pumila | B. pumila | A |
| Liardtrail_P3_10 | T | B. pumila | B. pumila | A |
| Liardtrail_P3_12 | T | B. pumila | B. pumila | C |
| Liardtrail_P3_13 | PT | B. pumila | B. pumila | A |
| Liardtrail_P3_15 | PT | B. pumila | B. pumila | A |
| NWTBC_border_P1_1 | T | B. pumila | B. pumila | A |
| NWTBC_border_P1_2 | T | B. pumila | B. pumila | A |
| NWTBC_border_P1_3 | T | B. pumila | B. pumila | A |
| NWTBC_border_P1_5 | T | B. pumila | B. pumila | A |
| NWTBC_border_P1_7 | T | B. pumila | B. pumila | A |
| NWTBC_border_P1_12 | T | B. pumila | B. pumila | A |
| BG_LakeLouise1_1 | D | B. glandulosa west | excluded | A |
| BG_LakeLouise1_2 | D | B. glandulosa west | excluded | M |
| BG_LakeLouise1_3 | D | B. glandulosa west | excluded | A |
| BG_LakeLouise1_4 | D | B. glandulosa west | B. glandulosa west | M |
| BG_LakeLouise1_5 | D | B. glandulosa west | excluded | A |
| BG_LakeLouise1_6 | D | B. glandulosa west | B. glandulosa west | M |
| BG_LakeLouise1_7 | D | B. glandulosa west | excluded | A |
| BG_LakeLouise1_8 | D | B. glandulosa west | excluded | A |
| BG_LakeLouise1_9 | D | B. glandulosa west | excluded | A |
| BG_LakeLouise1_10 | D | B. glandulosa west | B. glandulosa west | A |
| BG_LakeLouise1_11 | D | B. glandulosa west | B. glandulosa west | A |
| BG_LakeLouise1_12 | PD | B. glandulosa west | B. glandulosa west | A |
| BG_LakeLouise1_13 | D | B. glandulosa west | B. glandulosa west | A |
| BG_LakeLouise1_14 | D | B. glandulosa west | B. glandulosa west | A |
| BG_LakeLouise1_15 | D | B. glandulosa west | excluded | M |
| BG_LakeLouise1_16 | D | B. glandulosa west | excluded | A |
| BG_LakeLouise1_17 | D | B. glandulosa west | B. glandulosa west | A |
| BG_LLouise2_b2 | D | B. glandulosa west | excluded | A |
| BG_LLouise2_b3 | D | B. glandulosa west | B. glandulosa west | A |
| BG_LLouise2_b6 | D | B. glandulosa west | B. glandulosa west | A |
| BG_LLouise2_b8 | D | B. glandulosa west | B. glandulosa west | A |
| BG_LLouise2_b10 | D | B. glandulosa west | B. glandulosa west | A |
| BG_LLouise2_b11 | D | B. glandulosa west | excluded | A |
| BG_LakeLouise2_12 | TR | B. glandulosa west | B. glandulosa west | A |
| BG_LakeLouise2_13 | PD | B. glandulosa west | excluded | A |
| BG_LakeLouise2_14 | PD | B. glandulosa west | B. glandulosa west | A |
| BG_LakeLouise2_15 | PD | B. glandulosa west | B. glandulosa west | A |
| BG_LakeLouise2_16 | D | B. glandulosa west | B. glandulosa west | A |
| BG_LakeLouise2_17 | D | B. glandulosa west | B. glandulosa west | A |
| BG_LakeLouise2_18 | D | B. glandulosa west | excluded | A |
| Gaboriau_P4_1 | T | B. pumila | B. pumila | A |
| Gaboriau_P4_2 | T | B. pumila | B. pumila | M |
| Gaboriau_P4_3 | T | B. pumila | B. pumila | D |
| Gaboriau_P4_4 | T | B. pumila | B. pumila | D |
| Gaboriau_P4_5 | T | B. pumila | B. pumila | M |
| Gaboriau_P4_6 | T | B. pumila | B. pumila | M |
| Gaboriau_P4_7 | T | B. pumila | B. pumila | M |
| Gaboriau_P4_8 | T | B. pumila | B. pumila | D |
| Gaboriau_P4_9 | T | B. pumila | B. pumila | D |
| Gaboriau_P4_10 | T | B. pumila | B. pumila | M |
| Gaboriau_P4_11 | T | B. pumila | B. pumila | D |
| Gaboriau_P4_12 | T | B. pumila | B. pumila | D |
| Gaboriau_P4_13 | PT | B. pumila | excluded | NA |
| Gaboriau_P4_14 | T | B. pumila | B. pumila | M |
| Gaboriau_P4_15 | T | B. pumila | B. pumila | D |
| Gaboriau_P6_1 | T | B. pumila | B. pumila | C |
| Gaboriau_P6_2 | TR | Hybrid B. occidentalis | excluded | A |
| Gaboriau_P6_3 | T | B. pumila | B. pumila | C |
| Gaboriau_P6_4 | T | B. pumila | B. pumila | C |
| Gaboriau_P6_5 | PT | B. pumila | B. pumila | H |
| Gaboriau_P6_6 | T | B. pumila | B. pumila | C |
| Gaboriau_P6_7 | T | B. pumila | excluded | C |
| Gaboriau_P6_8 | D | B. glandulosa west | excluded | C |
| Gaboriau_P6_9 | T | B. pumila | B. pumila | C |
| Gaboriau_P6_10 | T | B. pumila | excluded | C |
| Gaboriau_P6_11 | D | B. glandulosa west | excluded | A |
| Gaboriau_P6_12 | T | B. pumila | B. pumila | C |
| Gaboriau_P6_13 | T | B. pumila | B. pumila | C |
| Gaboriau_P6_14 | T | B. pumila | B. pumila | C |
| Gaboriau_P6_15 | T | B. pumila | B. pumila | C |
| IZAAC_P1_1 | D | B. glandulosa west | excluded | C |
| IZAAC_P1_2 | U | Hybrid B. occidentalis | Hybrid B. occidentalis | M |
| IZAAC_P1_3 | T | B. pumila | B. pumila | M |
| IZAAC_P1_4 | D | B. glandulosa west | Hybrid B. occidentalis | C |
| IZAAC_P1_5 | D | Hybrid B. occidentalis | Hybrid B. occidentalis | C |
| IZAAC_P1_6 | U | Hybrid B. occidentalis | Hybrid B. occidentalis | A |
| IZAAC_P1_7 | D | B. glandulosa west | Hybrid B. occidentalis | A |
| IZAAC_P1_8 | D | Hybrid B. occidentalis | Hybrid B. occidentalis | A |
| IZAAC_P1_9 | D | B. glandulosa west | Hybrid B. occidentalis | F |
| IZAAC_P1_10 | D | Hybrid B. occidentalis | Hybrid B. occidentalis | C |
| IZAAC_P1_11 | D | B. glandulosa west | Hybrid B. occidentalis | D |
| IZAAC_P1_12 | D | Hybrid B. occidentalis | Hybrid B. occidentalis | A |
| IZAAC_P1_12b | D | Hybrid B. occidentalis | excluded | A |
| IZAAC_P1_13 | D | Hybrid B. occidentalis | Hybrid B. occidentalis | C |
| IZAAC_P1_13b | D | B. glandulosa west | Hybrid B. occidentalis | C |
| IZAAC_P1_13c | D | B. glandulosa west | Hybrid B. occidentalis | C |
| BG_WoodBuffalo_2_1 | D | B. glandulosa west | excluded | M |
| BG_WoodBuffalo_2_2 | D | B. glandulosa west | excluded | M |
| BG_WoodBuffalo_2_3 | D | B. glandulosa west | excluded | M |
| BG_WoodBuffalo_2_4 | D | B. glandulosa west | excluded | M |
| BG_WoodBuffalo_2_5 | D | B. glandulosa west | excluded | M |
| BG_WoodBuffalo_2_7 | D | B. glandulosa west | excluded | M |
| BG_WoodBuffalo_2_8 | D | B. glandulosa west | excluded | M |
| BG_WoodBuffalo_2_9 | D | B. glandulosa west | excluded | M |
| BG_WoodBuffalo_2_10 | D | B. glandulosa west | excluded | M |
| BG_WoodBuffalo_2_11 | D | B. glandulosa west | excluded | M |
| BG_WoodBuffalo_2_12 | D | B. glandulosa west | excluded | M |
| BG_WoodBuffalo_2_13 | D | B. glandulosa west | excluded | M |
| BG_WoodBuffalo_2_14 | D | B. glandulosa west | excluded | M |
| BG_WoodBuffalo_2_15 | D | B. glandulosa west | excluded | M |
| BG_WoodBuffalo_1_1 | D | B. glandulosa west | excluded | A |
| BG_WoodBuffalo_1_2 | D | B. glandulosa west | excluded | M |
| BG_WoodBuffalo_1_3 | D | B. glandulosa west | excluded | M |
| BG_WoodBuffalo_1_4 | D | B. glandulosa west | excluded | M |
| BG_WoodBuffalo_1_5 | D | B. glandulosa west | excluded | NA |
| BG_WoodBuffalo_1_6 | D | B. glandulosa west | excluded | NA |
| BG_WoodBuffalo_1_7 | D | B. glandulosa west | excluded | M |
| BG_WoodBuffalo_1_8 | PT | B. pumila | B. pumila | NA |
| BG_WoodBuffalo_1_9 | D | B. glandulosa west | excluded | M |
| BG_WoodBuffalo_1_10 | T | B. pumila | B. pumila | M |
| BG_WoodBuffalo_1_11 | D | B. glandulosa west | excluded | A |
| BG_WoodBuffalo_1_12 | D | B. glandulosa west | excluded | M |
| BG_WoodBuffalo_1_13 | D | B. glandulosa west | excluded | M |
| BG_WoodBuffalo_1_14 | D | B. glandulosa west | excluded | M |
| BG_WoodBuffalo_1_15 | T | B. pumila | B. pumila | M |
| BG_Elk2_b1 | PT | B. pumila | B. pumila | M |
| BG_Elk2_b2 | T | B. pumila | B. pumila | M |
| BG_Elk2_b3 | T | B. pumila | B. pumila | M |
| BG_Elk2_b4 | T | B. pumila | B. pumila | M |
| BG_Elk2_b5 | D | Hybrid B. occidentalis | excluded | M |
| BG_Elk3_b1 | T | B. pumila | B. pumila | M |
| BG_Elk3_b2 | TR | B. pumila | B. pumila | C |
| BG_Elk3_b3 | T | B. pumila | B. pumila | C |
| BG_Elk3_b4 | PT | B. pumila | B. pumila | C |
| BG_Elk3_b5 | TR | B. pumila | B. pumila | C |
| BG28 | D | B. glandulosa west | excluded | A |
| BG30 | D | B. glandulosa west | B. glandulosa west | C |
| DaringLake_P1_1b | D | B. glandulosa west | excluded | F |
| DaringLake_P1_2 | D | B. glandulosa west | B. glandulosa west | A |
| DaringLake_P1_3 | D | B. glandulosa west | excluded | A |
| DaringLake_P1_4 | D | B. glandulosa west | excluded | A |
| DaringLake_P1_5 | D | B. glandulosa west | B. glandulosa west | F |
| DaringLake_P1_6 | D | B. glandulosa west | excluded | A |
| DaringLake_P1_7 | D | B. glandulosa west | excluded | C |
| DaringLake_P1_8 | D | B. glandulosa west | B. glandulosa west | A |
| DaringLake_P1_9 | D | B. glandulosa west | excluded | M |
| DaringLake_P1_10b | D | B. glandulosa west | excluded | D |
| DaringLake_P1_11 | PD | B. glandulosa west | B. glandulosa west | NA |
| DaringLake_P1_12 | D | B. glandulosa west | excluded | A |
| DaringLake_P1_13 | D | B. glandulosa west | excluded | D |
| DaringLake_P1_14 | D | B. glandulosa west | excluded | A |
| DaringLake_P1_15 | D | B. glandulosa west | excluded | M |
| BG_LaRonge_1 | T | B. pumila | B. pumila | M |
| BG_LaRonge_2 | T | B. pumila | B. pumila | M |
| BG_LaRonge_3 | P | B. pumila | B. pumila | M |
| BG_LaRonge_4 | T | B. pumila | B. pumila | M |
| BG_LaRonge_5 | PT | B. pumila | B. pumila | M |
| BG_LaRonge_6 | T | B. pumila | B. pumila | C |
| BG_LaRonge_7 | T | B. pumila | B. pumila | M |
| BG_LaRonge_8 | T | B. pumila | B. pumila | M |
| BG_LaRonge_9 | T | B. pumila | B. pumila | M |
| BG_LaRonge_11 | T | B. pumila | B. pumila | M |
| BG_LaRonge_12 | PT | B. pumila | B. pumila | M |
| BG_LaRonge_13 | T | B. pumila | B. pumila | M |
| BG_LaRonge_14 | TR | B. pumila | B. pumila | M |
| BG_LaRonge_15 | T | B. pumila | B. pumila | C |
| SoabSouth_MB_P2_1 | PT | B. pumila | B. pumila | M |
| SoabSouth_MB_P2_2 | PT | B. pumila | B. pumila | M |
| SoabSouth_MB_P2_3 | PT | B. pumila | B. pumila | M |
| HorseStable_MB_P1_3 | T | B. pumila | B. pumila | M |
| Jonas_MB_P4_15 | T | B. pumila | excluded | M |
| BG1 | D | B. glandulosa west | B. glandulosa west | D |
| BG3 | PD | B. glandulosa west | B. glandulosa west | T |
| Gillam_MB_P3_13 | PT | B. pumila | B. pumila | A |
| Gillam_MB_P3_14 | PT | B. pumila | B. pumila | A |
| Gillam_MB_P3_15 | PT | B. pumila | excluded | NA |
| BG116 | PT | B. pumila | B. pumila | M |
| BG117_2_Churchill_site_3_feuille | PT | B. pumila | B. pumila | D |
| BG111_2_Churchill_site_1_feuille | PT | B. pumila | B. pumila | M |
| BG112_3_Churchill_site_1_feuille | PT | B. pumila | B. pumila | NA |
| BG113 | PT | B. pumila | B. pumila | D |
| BG114_2_Churchill_site_2_feuille | T | B. pumila | B. pumila | M |
| BG115_3_Churchill_site_2_feuille | T | B. pumila | B. pumila | NA |
| BG22 | PT | B. pumila | B. pumila | NA |
| BG_Taillon3_f1 | D | B. glandulosa east | excluded | M |
| BG_Taillon3_f2 | D | B. glandulosa east | excluded | B |
| BG_Taillon3_f3 | D | B. glandulosa west | excluded | NA |
| BG_Taillon3_f4 | D | B. glandulosa east | excluded | B |
| BG_Taillon3_f6 | D | B. glandulosa west | excluded | D |
| BG_Taillon3_f7 | D | B. glandulosa east | excluded | D |
| BG_Taillon3_f8 | D | B. glandulosa east | excluded | D |
| BG_Taillon3_f9 | D | B. glandulosa east | excluded | A |
| BG_Taillon3_f10 | D | B. glandulosa east | excluded | B |
| BG_Taillon3_f11 | D | B. glandulosa east | excluded | D |
| BG_Taillon3_f14 | T | B. glandulosa east | excluded | B |
| BG_Taillon3_f15 | D | B. glandulosa east | excluded | D |
| Transtaiga_P4_2 | D | B. glandulosa east | B. glandulosa east | B |
| Transtaiga_P4_3 | D | B. glandulosa east | B. glandulosa east | B |
| Transtaiga_P4_4 | D | B. glandulosa east | excluded | B |
| Transtaiga_P4_5 | T | B. glandulosa east | B. glandulosa east | O |
| Transtaiga_P4_6 | D | B. glandulosa east | B. glandulosa east | B |
| Transtaiga_P4_7 | D | B. glandulosa east | B. glandulosa east | B |
| Transtaiga_P4_8 | D | B. glandulosa east | B. glandulosa east | O |
| Transtaiga_P4_9 | D | B. glandulosa east | B. glandulosa east | O |
| Transtaiga_P4_10 | D | B. glandulosa east | B. glandulosa east | B |
| Transtaiga_P4_11 | D | B. glandulosa east | B. glandulosa east | O |
| Transtaiga_P4_12 | D | B. glandulosa east | B. glandulosa east | O |
| Transtaiga_P4_13 | D | B. glandulosa east | B. glandulosa east | B |
| Transtaiga_P4_14 | D | B. glandulosa east | B. glandulosa east | B |
| Transtaiga_P4_15 | D | B. glandulosa east | B. glandulosa east | B |
| BG68 | D | B. glandulosa east | excluded | O |
| BG69 | PD | B. glandulosa east | excluded | O |
| BG71 | PD | B. glandulosa east | excluded | O |
| Transtaiga_P3_1 | D | B. glandulosa east | B. glandulosa east | N |
| Transtaiga_P3_2 | D | B. glandulosa east | B. glandulosa east | N |
| Transtaiga_P3_3 | D | B. glandulosa east | B. glandulosa east | N |
| Transtaiga_P3_4 | D | B. glandulosa east | B. glandulosa east | N |
| Transtaiga_P3_5 | D | B. glandulosa east | B. glandulosa east | N |
| Transtaiga_P3_6 | D | B. glandulosa east | B. glandulosa east | N |
| Transtaiga_P3_7 | D | B. glandulosa east | B. glandulosa east | N |
| Transtaiga_P3_8 | D | B. glandulosa east | B. glandulosa east | N |
| Transtaiga_P3_9 | D | B. glandulosa east | B. glandulosa east | P |
| Transtaiga_P3_10 | D | B. glandulosa east | B. glandulosa east | P |
| Transtaiga_P3_11 | D | B. glandulosa east | B. glandulosa east | P |
| Transtaiga_P3_12 | D | B. glandulosa east | B. glandulosa east | N |
| Transtaiga_P3_13 | D | B. glandulosa east | B. glandulosa east | N |
| Transtaiga_P3_14 | D | B. glandulosa east | B. glandulosa east | N |
| Transtaiga_P3_15 | D | B. glandulosa east | B. glandulosa east | N |
| BG_Taillon1_f1 | D | B. glandulosa east | excluded | B |
| BG_Taillon1_f2 | D | B. glandulosa east | excluded | M |
| BG_Taillon1_f3 | D | B. glandulosa east | excluded | B |
| BG_Taillon1_f4 | D | B. glandulosa east | B. glandulosa east | B |
| BG_Taillon1_f5 | D | B. glandulosa east | excluded | B |
| BG_Taillon1_f7 | D | B. glandulosa east | excluded | M |
| BG_Taillon1_f8 | D | B. glandulosa east | B. glandulosa east | O |
| BG_Taillon1_f9 | D | B. glandulosa east | excluded | B |
| BG_Taillon1_f10 | D | B. glandulosa east | B. glandulosa east | B |
| BG_Taillon1_f11 | D | B. glandulosa east | B. glandulosa east | N |
| BG_Taillon1_f12 | D | B. glandulosa east | B. glandulosa east | N |
| BG_Taillon1_f13 | D | B. glandulosa east | B. glandulosa east | N |
| BG_Taillon1_f14 | D | B. glandulosa east | B. glandulosa east | N |
| BG_Taillon1_f15 | D | B. glandulosa east | B. glandulosa east | N |
| BG_Taillon9_f1 | D | B. glandulosa east | excluded | M |
| BG_Taillon9_f2 | D | B. glandulosa east | B. glandulosa east | M |
| BG_Taillon9_f3 | D | B. glandulosa east | B. glandulosa east | O |
| BG_Taillon9_f4 | D | B. glandulosa east | B. glandulosa east | D |
| BG_Taillon9_f5 | D | B. glandulosa east | B. glandulosa east | O |
| BG_Taillon9_f6 | D | B. glandulosa east | B. glandulosa east | M |
| BG_Taillon9_f7 | D | B. glandulosa east | B. glandulosa east | M |
| BG_Taillon9_f8 | D | B. glandulosa east | B. glandulosa east | O |
| BG_Taillon9_f9 | D | B. glandulosa east | B. glandulosa east | M |
| BG_Taillon9_f11 | D | B. glandulosa east | B. glandulosa east | O |
| BG_Taillon9_f13 | D | B. glandulosa east | B. glandulosa east | M |
| Transtaiga_P2_1 | T | B. glandulosa east | B. glandulosa east | B |
| Transtaiga_P2_2 | D | B. glandulosa east | B. glandulosa east | N |
| Transtaiga_P2_3 | D | B. glandulosa east | B. glandulosa east | B |
| Transtaiga_P2_4 | D | B. glandulosa east | B. glandulosa east | N |
| Transtaiga_P2_5 | D | B. glandulosa east | B. glandulosa east | O |
| Transtaiga_P2_6 | D | B. glandulosa east | B. glandulosa east | N |
| Transtaiga_P2_7 | D | B. glandulosa east | B. glandulosa east | N |
| Transtaiga_P2_8 | D | B. glandulosa east | B. glandulosa east | B |
| Transtaiga_P2_9 | D | B. glandulosa east | B. glandulosa east | N |
| Transtaiga_P2_10 | D | B. glandulosa east | B. glandulosa east | K |
| Transtaiga_P2_11 | D | B. glandulosa east | B. glandulosa east | B |
| Transtaiga_P2_12 | D | B. glandulosa east | B. glandulosa east | N |
| Transtaiga_P2_13 | D | B. glandulosa east | B. glandulosa east | N |
| Transtaiga_P2_14 | D | B. glandulosa east | B. glandulosa east | B |
| Transtaiga_P2_15 | D | B. glandulosa east | B. glandulosa east | Q |
| Transtaiga_P1_1 | D | B. glandulosa east | B. glandulosa east | B |
| Transtaiga_P1_2 | D | B. glandulosa east | B. glandulosa east | B |
| Transtaiga_P1_3 | D | B. glandulosa east | B. glandulosa east | B |
| Transtaiga_P1_4 | D | B. glandulosa east | B. glandulosa east | B |
| Transtaiga_P1_5 | D | B. glandulosa east | B. glandulosa east | B |
| Transtaiga_P1_6 | D | B. glandulosa east | B. glandulosa east | B |
| Transtaiga_P1_7 | D | B. glandulosa east | B. glandulosa east | B |
| Transtaiga_P1_8 | D | B. glandulosa east | B. glandulosa east | B |
| Transtaiga_P1_9 | D | B. glandulosa east | B. glandulosa east | B |
| Transtaiga_P1_10 | D | B. glandulosa east | B. glandulosa east | N |
| Transtaiga_P1_11 | D | B. glandulosa east | B. glandulosa east | B |
| Transtaiga_P1_12 | D | B. glandulosa east | B. glandulosa east | N |
| Transtaiga_P1_13 | D | B. glandulosa east | B. glandulosa east | B |
| Transtaiga_P1_14 | D | B. glandulosa east | B. glandulosa east | O |
| Transtaiga_P1_15 | D | B. glandulosa east | B. glandulosa east | B |
| BG_Taillon_12_f2 | D | B. glandulosa east | B. glandulosa east | D |
| BG_Taillon_12_f4 | PD | B. glandulosa east | B. glandulosa east | D |
| BG_Taillon_12_f8 | D | B. glandulosa east | B. glandulosa east | D |
| BG_Taillon_12_f9 | D | B. glandulosa east | B. glandulosa east | O |
| BG_Taillon12_f1 | D | B. glandulosa east | B. glandulosa east | D |
| BG_Taillon12_f3 | D | B. glandulosa east | B. glandulosa east | D |
| BG_Taillon12_f5 | D | B. glandulosa east | B. glandulosa east | D |
| BG_Taillon12_f6 | D | B. glandulosa east | B. glandulosa east | D |
| BG_Taillon12_f7 | D | B. glandulosa east | B. glandulosa east | D |
| BG_Taillon_12_f10 | D | B. glandulosa east | B. glandulosa east | D |
| BG_Taillon_12_f11 | D | B. glandulosa east | B. glandulosa east | D |
| BG_Taillon_12_f12 | D | B. glandulosa east | B. glandulosa east | O |
| BG_Taillon_12_f13 | D | B. glandulosa east | B. glandulosa east | D |
| BG_Taillon_12_f14 | D | B. glandulosa east | B. glandulosa east | D |
| BG_Taillon_12_f15 | D | B. glandulosa east | B. glandulosa east | D |
| Fermont488_1 | D | B. glandulosa east | B. glandulosa east | B |
| Fermont488_2 | D | B. glandulosa east | B. glandulosa east | N |
| Fermont488_3 | D | B. glandulosa east | B. glandulosa east | B |
| Fermont488_4 | D | B. glandulosa east | B. glandulosa east | B |
| Fermont488_5 | D | B. glandulosa east | B. glandulosa east | N |
| Fermont488_6 | D | B. glandulosa east | B. glandulosa east | N |
| Fermont488_7 | D | B. glandulosa east | B. glandulosa east | B |
| Fermont488_8 | D | B. glandulosa east | B. glandulosa east | O |
| Fermont488_9 | D | B. glandulosa east | B. glandulosa east | N |
| Fermont488_10 | D | B. glandulosa east | B. glandulosa east | O |
| Fermont488_11 | D | B. glandulosa east | B. glandulosa east | B |
| Fermont488_12 | D | B. glandulosa east | B. glandulosa east | B |
| Fermont488_13 | D | B. glandulosa east | B. glandulosa east | N |
| Fermont488_14 | D | B. glandulosa east | B. glandulosa east | O |
| Fermont488_15 | D | B. glandulosa east | B. glandulosa east | N |
| Fermont488_16 | D | B. glandulosa east | B. glandulosa east | N |
| Fermont390_1 | D | B. glandulosa east | B. glandulosa east | O |
| Fermont390_2 | D | B. glandulosa east | B. glandulosa east | N |
| Fermont390_3 | D | B. glandulosa east | excluded | O |
| Fermont390_4 | D | B. glandulosa east | B. glandulosa east | N |
| Fermont390_5 | D | B. glandulosa east | B. glandulosa east | O |
| Fermont390_6 | D | B. glandulosa east | B. glandulosa east | O |
| Fermont390_7 | D | B. glandulosa east | B. glandulosa east | O |
| Fermont390_8 | D | B. glandulosa east | B. glandulosa east | N |
| Fermont390_10 | D | B. glandulosa east | B. glandulosa east | N |
| Fermont390_11 | D | B. glandulosa east | B. glandulosa east | O |
| Fermont390_12 | D | B. glandulosa east | B. glandulosa east | N |
| Fermont390_13 | D | B. glandulosa east | B. glandulosa east | N |
| Fermont390_14 | D | B. glandulosa east | B. glandulosa east | B |
| Fermont390_15 | D | B. glandulosa east | B. glandulosa east | O |
| Fermont390_16 | D | B. glandulosa east | B. glandulosa east | O |
| Fermont390_17 | D | B. glandulosa east | B. glandulosa east | N |
| BG152 | D | B. glandulosa east | B. glandulosa east | B |
| BG153 | D | B. glandulosa east | B. glandulosa east | B |
| BG155 | D | B. glandulosa east | B. glandulosa east | B |
| BG156 | D | B. glandulosa east | B. glandulosa east | J |
| BG157 | D | B. glandulosa east | B. glandulosa east | B |
| BG_Mingan_1 | D | B. glandulosa east | B. glandulosa east | B |
| BG_Mingan_2 | D | B. glandulosa east | B. glandulosa east | B |
| BG_Mingan_3 | D | B. glandulosa east | B. glandulosa east | O |
| BG_Mingan_4 | D | B. glandulosa east | B. glandulosa east | B |
| BG_Mingan_5 | D | B. glandulosa east | B. glandulosa east | B |
| BG_Mingan_6 | D | B. glandulosa east | B. glandulosa east | B |
| BG_Mingan_8 | D | B. glandulosa east | B. glandulosa east | B |
| BG_Mingan_9 | D | B. glandulosa east | B. glandulosa east | B |
| BG_Mingan_11 | D | B. glandulosa east | B. glandulosa east | B |
| BG_Mingan_12 | D | B. glandulosa east | B. glandulosa east | B |
| BG_Mingan_13 | D | B. glandulosa east | B. glandulosa east | B |
| BG_Mingan_14 | D | B. glandulosa east | B. glandulosa east | B |
| BG_Mingan_15 | D | B. glandulosa east | B. glandulosa east | B |
| BG_TRG3_f2 | PD | B. glandulosa east | B. glandulosa east | O |
| BG_TRG3_f6 | D | B. glandulosa east | B. glandulosa east | O |
| BG_TRG3_f8 | PD | B. glandulosa east | B. glandulosa east | O |
| BG_TRG2_f15 | D | B. glandulosa east | B. glandulosa east | NA |
| BG54 | D | B. glandulosa east | B. glandulosa east | O |
| GooseBay_Cside_1 | D | B. glandulosa east | B. glandulosa east | B |
| GooseBay_Cside_2 | D | B. glandulosa east | B. glandulosa east | B |
| GooseBay_Cside_3 | D | B. glandulosa east | B. glandulosa east | O |
| GooseBay_Cside_4 | D | B. glandulosa east | B. glandulosa east | N |
| GooseBay_Cside_4a | D | B. glandulosa east | B. glandulosa east | O |
| GooseBay_Cside_5 | D | B. glandulosa east | B. glandulosa east | B |
| GooseBay_Cside_6 | D | B. glandulosa east | B. glandulosa east | N |
| GooseBay_Cside_8 | D | B. glandulosa east | B. glandulosa east | N |
| GooseBay_Cside_9 | D | B. glandulosa east | B. glandulosa east | N |
| GooseBay_Cside_10 | D | B. glandulosa east | B. glandulosa east | O |
| GooseBay_Cside_11 | D | B. glandulosa east | B. glandulosa east | O |
| GooseBay_Cside_12 | D | B. glandulosa east | B. glandulosa east | B |
| GooseBay_Cside_13 | D | B. glandulosa east | B. glandulosa east | B |
| GooseBay_Cside_14 | D | B. glandulosa east | B. glandulosa east | N |
| GooseBay_Cside_15 | PD | B. glandulosa east | B. glandulosa east | N |
| GooseBay_Bike_1 | D | B. glandulosa east | B. glandulosa east | N |
| GooseBay_Bike_2 | D | B. glandulosa east | B. glandulosa east | N |
| GooseBay_Bike_3 | D | B. glandulosa east | B. glandulosa east | B |
| GooseBay_Bike_4 | D | B. glandulosa east | B. glandulosa east | N |
| GooseBay_Bike_5 | D | B. glandulosa east | B. glandulosa east | N |
| GooseBay_Bike_6 | D | B. glandulosa east | B. glandulosa east | O |
| GooseBay_Bike_7 | D | B. glandulosa east | B. glandulosa east | Q |
| GooseBay_Bike_8 | D | B. glandulosa east | B. glandulosa east | O |
| GooseBay_Bike_9 | D | B. glandulosa east | B. glandulosa east | S |
| GooseBay_Bike_10 | D | B. glandulosa east | B. glandulosa east | O |
| GooseBay_Bike_11 | D | B. glandulosa east | B. glandulosa east | O |
| GooseBay_Bike_12 | D | B. glandulosa east | excluded | N |
| GooseBay_Bike_13 | D | B. glandulosa east | B. glandulosa east | N |
| GooseBay_Bike_14 | D | B. glandulosa east | B. glandulosa east | N |
| GooseBay_Bike_15 | D | B. glandulosa east | B. glandulosa east | B |
| BG127 | U | Putative B. nana | Europe | G |
| BG131 | U | Putative B. nana | Europe | G |
| BG133 | U | Putative B. nana | Europe | G |
| BG130 | U | Putative B. nana | excluded | E |
| BG121 | D | Putative B. nana | Europe | I |
| BG122 | D | Putative B. nana | Europe | E |
| Bg123 | T | Putative B. nana | Europe | E |
| BG124 | D | Putative B. nana | Europe | E |
| Betula_glandulosa_HLM_QFA364259 | PD | B. glandulosa east | B. glandulosa east | O |
| Kew_glandulosa_19950 | U | B. pumila | excluded | A |

a: D = diploid, P = pentaploid, PD = putative diploid, PT = putative tetraploid, T = tetraploid, TR = triploid, U = unknown.

b: NA = Individuals without chlorotype assignment

##### **Table S4.** Genetic diversity indices obtained across genetic clusters (DAPC groups) and across all populations, respectively.

|  | ***H_O_*** | ***H_S_*** | ***F_IS_*** | ***F_ST_*** |
| --- | --- | --- | --- | --- |
| DAPC groups (n = 5) | 0.1341 | 0.1129 | -0.1874 | 0.1352 |
| All populations (n = 74) | 0.1414 | 0.1132 | -0.2498 | 0.1681 |
